# Machine learning risk prediction of mortality for patients undergoing surgery with perioperative SARS-CoV-2: the COVIDSurg mortality score

**DOI:** 10.1093/bjs/znab183

**Published:** 2021-07-06

**Authors:** COVIDSurg Collaborative, I Dajti, J I Valenzuela, L A Boccalatte, N A Gemelli, D E Smith, N N Dudi-Venkata, H M Kroon, T Sammour, M Roberts, D Mitchell, K Lah, A Pearce, A Morton, A C Dawson, A Drane, C Sharpin, R M Nataraja, M Pacilli, D R A Cox, V Muralidharan, G E Riddiough, E M Clarke, W Jamel, K R Qin, P Pockney, D Cope, N Egoroff, N Lott, S Putnis, S De Robles, Z Ang, M Mitteregger, S Uranitsch, M Stiegler, G Seitinger, F Aigner, D B Lumenta, P S Nischwitz, E Richtig, M Pau, P Srekl-Filzmaier, N Eibinger, B Michelitsch, M Fediuk, A Papinutti, G Seidel, J Kahn, T U Cohnert, D Messner, F Öfner, J Presl, M Varga, M Weitzendorfer, K Emmanuel, A D Binder, M Zimmermann, S Holawe, E Nkenke, C Grimm, M Kranawetter, A Rahman Mitul, N Islam, S Karim, N Komen, S Putnis, S De Robles, E Ang, H De Praetere, T Tollens, G Schols, C Smets, L Haenen, J Quintens, K Van Belle, G H Van Ramshorst, P Pattyn, L Desender, T Martens, D Van de Putte, P Lerut, A Grimonprez, M Janssen, G De Smul, P Wallaert, J Van den Eynde, W Oosterlinck, R Van den Eynde, A Sermon, A Boeckxstaens, A Cordonnier, J De Coster, J Jaekers, C Politis, M Miserez, N Duchateau, C De Gheldere, N Flamey, P Pattyn, A Christiano, B Guidi, A L Minussi, S Castro, W Okoba, F H R Maldonado, P Oliveira, T Baldasso, L Santos, G M A Gomes, I L Buarque, L Pol-Fachin, T S Bezerra, A V Barros, A M R da Silva, A L S Leite, D W A Silvestre, C C Ferro, M S Araujo, L M Lopes, P D Damasceno, D H S Araujo, G Laporte, M C Salem, M A C Guimaraes-Filho, L Nacif, R L G Flumignan, L C U Nakano, D A B Kuramoto, A L S Aidar, M R Pereda, R M Correia, B C Santos, A A Carvalho, J E Amorim, H J Guedes Neto, L L Areias, A F Sousa, C D Q Flumignan, W G Lustre, D H Moreno, N Barros, J C C Baptista-Silva, L L Matos, L P Kowaski, M A V Kulcsar, K S Nunes, M F Teixeira, R L Nunes, T R Ijichi, N J Kim, A Marreiro, B Muller, J Barakat Awada, G Baiocchi, L P Kowalski, J G Vartanian, F B Makdissi, S Aguiar, N Marques, G B Carvalho, T M D M Marques, E A Abdallah, C E Zurstrassen, J L Gross, S C Zequi, B T Gonçalves, S S Santos, J P Duprat, F J F Coimbra, R Cicco, F Takeda, I Cecconello, U Ribeiro, A Gatti, R Oliva, C Nardi, M Slavchev, B Atanasov, N Belev, A Dell, D Bigam, K Dajani, S Al Riyami, J Martin, D Cheng, H Yang, A Fayad, F M Carrier, E Amzallag, J Desroches, M Ruel, N G Caminsky, M Boutros, J Moon, E G Wong, T Vanounou, J Pelletier, S Wong, E Girsowicz, J Bayne, D Obrand, H Gill, O Steinmetz, K MacKenzie, M Lukaszewski, G Jamjoum, P Richebé, O Verdonck, S Discepola, N Godin, M Idrissi, V Lecluyse, G Côté, S Demyttenaere, R Garfinkle, M Boutros, A Kouyoumdjian, M Boutros, S Dumitra, J Moon, E G Wong, K Khwaja, L Luo, M Lukaszewski, H Gill, G Berry, A S Liberman, E Girsowicz, J Bayne, D Obrand, O Steinmetz, K MacKenzie, S Schmid, J Spicer, M Al Farsi, J Abou-Khalil, E Couture, S Mohammadi, H Tremblay, N Gagné, A Bergeron, A F Turgeon, O Costerousse, D Bellemare, C Babin, C Blier, M L Wood, A Persad, G Groot, H Pham, F D'Aragon, E Carbonneau, M Bouchard, M Masse, F Pesant, J Héroux, P Karanicolas, J Hallet, A Nadler, A Nathens, M Ko, A Brar, K Mayson, B Kidane, S Srinathan, M I Escudero, J T Reyes, M M Modolo, P Ramirez Nieto, R Sepulveda, A Bolbarán, A Molero, I Ruiz, G P Reyes, R Salas, C Suazo, R Muñoz, E Grasset, M Inzunza, N Besser, M J Irarrázaval, C Jarry, F Bellolio, C A Romero Manqui, M Ruiz Esquide, T Fuentes, J Campos, C J Perez Rivera, P A Cabrera, R E Pinilla, O Guevara, L J Jimenez Ramirez, B G Velasquez Cuasquen, D R Herrera Mora, A Bonilla, S Diaz, E Manrique, H Facundo, J L Velez Bernal, J ángel, M García, L Guzmán, C Lehmann, S Cervera, L M Trujillo Sanchez, R Guevara, D Valbuena, L Suarez, G Jimenez, A Velandia, J Vargas, J Espinosa, S Rey, J Mendoza Quevedo, J A Calvache, C M Orozco-Chamorro, T A Sánchez-Gómez, D A Rojas-Tejada, J Mihanovic, B Bakmaz, I Rakvin, N Sulen, T Andabaka, I Luksic, M Mamic, L Martinek, M Skrovina, J Žatecký, M Peteja, H Ø Kristensen, M Mekhael, P Christensen, L Westh, H Smith, A F Haugstvedt, M L JÖnsson, A Crespo, S Batista, J Rodriguez-Abreu, N Tactuk, P J Diaz-Delgado, R Rivas, J A Sarmiento-Bobadilla, F Ashoush, A Samir Abdelaal, M S Qatora, M E Elsayed Hewalla, M Metwalli, R Atta, A Abdelmajeed, N E Abosamak, A Sabry, S Shehata, I Sallam, G Amira, M Sherief, A Sherif, H Salem, R Hamdy, H Aboulkassem, G Ghaly, G Sherif, A Morsi, A Abdelrahman, A Omnia, A Tawheed, M El Kassas, W Omar, A Abdelsamed, A Seleim, H Salem, A Y Azzam, M ElFiky, A Nabil, M Ibraheem, G Ghaly, M ElDeeb, M Fawzy, H Hamed, S Emile, A Elfallal, H Elfeki, M Shalaby, A Sakr, M Alrahawy, H Atif, A Sakr, H Soltan, A K Sayed, A Salah, A Atiya, K Wassim, E Esmail, M Khalaf, A Eldaly, A Ghoneim, A Hawila, H Badr, I Elhalaby, M Abdel-bari, M Elbahnasawy, M K Hamada, M S Morsy, M Hammad, M Hammad, M Essa, M T Fayed, M Elzoghby, M Rady, O Hamad, S Salman, S Sarsik, S Abd-elsalam, S Gamal Badr, Y El-Masry, M M H Moahmmed, S Hailu, A Wolde, M Mengesha, S Nida, M Workneh, M Y Ahmed, T Fisseha, D Kassa, H Zeleke, A Admasu, T Laeke, A Tirsit, M Gessesse, A Addissie, K Bekele, J H Kauppila, E Sarjanoja, S Testelin, S Dakpé, B Devauchelle, J Bettoni, N Lavagen, F Schmitt, J M Lemée, S Boucher, R Breheret, J D Kün-Darbois, A Kahn, A Gueutier, P Bigot, B Borraccino, Z Lakkis, A Doussot, B Heyd, S Manfredelli, P Mathieu, B Paquette, C Turco, A Barrabe, A Louvrier, D Moszkowicz, D Giovinazzo, F Bretagnol, A Police, L Charre, E Volpin, H Braham, N El Arbi, V Villefranque, L Bendjemar, E Girard, J Abba, B Trilling, A Chebaro, K Lecolle, S Truant, M El Amrani, P Zerbib, F R Pruvot, D Mathieu, E Surmei, L Mattei, H Marin, N Christou, Q Ballouhey, P Ferrero, P Coste Mazeau, J Tricard, B Barrat, A Taibi, J Usseglio, J Laloze, H Salle, L Fourcade, E Duchalais, N Regenet, J Rigaud, D Waast, W Denis, O Malard, K Buffenoir, F Espitalier, C Ferron, Y Varenne, V Crenn, S De Vergie, J Cristini, E Samarut, S Tzedakis, P A Bouche, S Gaujoux, E Kantor, D Gossot, A Seguin-Givelet, D Fuks, M Grigoroiu, R Sanchez Salas, X Cathelineau, P Macek, Y Barbé, F Rozet, E Barret, A Mombet, N Cathala, E Brian, F Zadegan, C Conso, T Blanc, A Broch, S Sarnacki, L Ali, A Bonnard, M Peycelon, E Hervieux, P Clermidi, E Maisonneuve, E Aubry, A Thomin, T Langlais, G Passot, O Glehen, E Cotte, J C Lifante, B De Simone, E Chouillard, A P Arnaud, P Violas, D Bergeat, A Merdrignac, A P Arnaud, A Scalabre, L O Perotto, B Le Roy, E Haddad, S Vermersch, A C Ezanno, O Barbier, F Vigouroux, B Malgras, A Aime, B Seeliger, D Mutter, G Philouze, P Pessaux, A Germain, H Chanty, A Ayav, R Kassir, P Von Theobald, F Sauvat, J O'Connor, M Mayombo Idiata, Z O'Connor, S Tchoba, A Modabber, P Winnand, F HÖlzle, B Sommer, E Shiban, S Wolf, M Anthuber, F Sommer, D Kaemmerer, T Schreiber, C Kamphues, J C Lauscher, C Schineis, F N Loch, K Beyer, S Nasser, J Sehouli, P HÖhn, C Braumann, F Reinkemeier, W Uhl, J Weitz, U Bork, T Welsch, C Praetorius, S Korn, M Distler, G Fluegen, W T Knoefel, C Vay, H Golcher, R Grützmann, J Binder, P Meister, A Gallinat, A Paul, A A Schnitzbauer, P Thoenissen, H El Youzouri, T Schreckenbach, T A Nguyen, H Eberbach, J Bayer, B Erdle, R Sandkamp, C Nitschke, J Izbicki, F G Uzunoglu, D Koenig, M Gosau, A BÖttcher, A Heuer, T O Klatte, M Priemel, C S Betz, S Burg, N MÖckelmann, C J Busch, J Bewarder, N Zeller, R Smeets, S Thole, T Vollkommer, U Speth, M Stangenberg, I Hakami, C Boeker, J Mall, H M Schardey, T von Ahnen, M von Ahnen, U Brunner, C Tapking, U Kneser, C Hirche, M Jung, K F Kowalewski, P Kienle, C Reissfelder, S Seyfried, F Herrle, J Hardt, C Galata, E Birgin, N Rahbari, N Vassos, M G Stoleriu, R Hatz, M Albertsmeier, N BÖrner, C Lampert, J Werner, B Kuehlmann, L Prantl, S M Brunner, H J Schlitt, F Brennfleck, K Pfister, K Oikonomou, T Reinhard, K Nowak, U Ronellenfitsch, J Kleeff, K S Delank, C W Michalski, G Szabo, R Widyaningsih, G A Stavrou, R Bschorer, J Mielke, T Peschel, A KÖnigsrainer, M Quante, M W LÖffler, C Yurttas, J Doerner, R Seiberth, K Bouchagier, S Klimopoulos, D Paspaliari, G Stylianidis, A Syllaios, E Baili, D Schizas, T Liakakos, A Charalabopoulos, C Zografos, E Spartalis, D K Manatakis, N Tasis, M I Antonopoulou, S Xenaki, E Xynos, E Chrysos, E Athanasakis, J Tsiaousis, E Lostoridis, P Tourountzi, G Tzovaras, K Tepetes, D Zacharoulis, I Baloyiannis, K Perivoliotis, J Hajiioannou, C Korais, E Gkrinia, C E Skoulakis, A Saratziotis, O Koukoura, D Symeonidis, A Diamantis, G Tsoulfas, C D Christou, A Tooulias, V Papadopoulos, C Anthoulakis, G Grimbizis, D Zouzoulas, D Tsolakidis Tatsis, P Christidis, L Loutzidou, O Ioannidis, I Astreidis, A Antoniou, K Antoniadis, K Vachtsevanos, K Paraskevopoulos, I Kalaitsidou, V Alexoudi, A Stavroglou, A Mantevas, D Michailidou, T Grivas, D Deligiannidis, S Politis, A Barrios Duarte, A L Portilla, M J Lowey, G Recinos, I Lopez Muralles, M A Siguantay, E E Estrada, M L Aguilera-Arévalo, J M Cojulun, G Echeverría-Dávila, C Marín, G C Icaza de Marín, Z Novak, T Echim, N Suszták, B Banky, G Kembuan, H Pajan, A A Islam, F Rahim, H Safari, M Mozafari, P Brouki Milan, A Tizmaghz, M Rezaei Tavirani, R Hussein, C Fleming, S O'Brien, M Y Kayyal, A Daly, S Killeen, M Corrigan, J De Marchi, A Hill, T Farrell, N F Davis, D Kearney, T Nelson, P J Maguire, C Barry, R Farrell, L A Smith, H M Mohan, B J Mehigan, P McCormick, J O Larkin, B A Fahey, A Rogers, N Donlon, H O'Sullivan, T Nugent, J V Reynolds, C Donohue, P Shokuhi, N Ravi, C Fitzgerald, P Lennon, C Timon, J Kinsella, J Smith, T Boyle, D Alazawi, E Connolly, W Butt, S M Croghan, R P Manecksha, N Fearon, D Winter, H Heneghan, D Maguire, T Gallagher, K Conlon, N Kennedy, S Martin, R Kennelly, A Hanly, K C Ng, J Fagan, E Geary, C Cullinane, A Hanly, E Carrington, J Geraghty, E McDermott, R Pritchard, D McPartland, M Boland, A Stafford, D Maguire, J Geoghegan, J A Elliott, P F Ridgway, A E Gillis, G A Bass, P C Neary, J M O'Riordan, D O Kavanagh, I S Reynolds, K Conlon, D P Joyce, E Boyle, B Egan, M Whelan, R Elkady, S Tierney, T M Connelly, H Earley, M Umair, C O'Connell, R P Manecksha, A Z Thomas, D Rice, A Madden, Y Bashir, B Creavin, O Cullivan, P Owens, A Canas-Martinez, C Murphy, L Pickett, B Murphy, A Mastrosimone, D Beddy, M Arumugasamy, M Allen, M Aremu, C McCarthy, C O’Connor, D B O'Connor, E Kent, F Malone, M Geary, K L McKevitt, A J Lowery, é J Ryan, T M Aherne, A Fowler, A Hassanin, A M Hogan, C G Collins, L Finnegan, P A Carroll, M J Kerin, S R Walsh, D Nally, C Peirce, J C Coffey, R M Cunningham, S Tormey, N P Hardy, P M Neary, L Muallem-Kalmovich, N Kugler, R Lavy, O Zmora, N Horesh, R Vergari, S Mochet, R Barmasse, A Usai, L Morelli, A Picciariello, V Papagni, D F Altomare, M Colledan, M F Zambelli, S Tornese, A Camillo, E Rausa, F Bianco, A Lucianetti, G M Prucher, A M Baietti, F Ruggiero, P Maremonti, F Neri, S Ricci, M Biasini, A G Zarabini, A Belvedere, P Bernante, P Bertoglio, S Boussedra, E Brunocilla, R Cipriani, G Cisternino, E De Crescenzo, P De Iaco, A N Della Gatta, G Dondi, F Frio, E Jovine, F Mineo Bianchi, J Neri, D Parlanti, A M Perrone, A P Pezzuto, M Pignatti, G Pilu, V Pinto, G Poggioli, M Ravaioli, M Rottoli, R Schiavina, M Serenari, M Serra, P Solli, M Taffurelli, M Tanzanu, M Tesei, T Violante, S Zanotti, V Tonini, L Sartarelli, M Cervellera, A Gori, G Armatura, G Scotton, S Patauner, A Frena, M Podda, A Pisanu, G Esposito, F Frongia, E Abate, L Laface, M Casati, M Schiavo, T Casiraghi, G Sammarco, G Gallo, G Vescio, S Fulginiti, V Scorcia, G Giannaccare, A Carnevali, M C Giuffrida, A Marano, S Palagi, S Di Maria Grimaldi, V Testa, C Peluso, F Borghi, A Simonato, A Puppo, M D'Agruma, R Chiarpenello, L Pellegrino, F Maione, D Cianflocca, V Pruiti Ciarello, G Giraudo, E Gelarda, E Dalmasso, A Abrate, A Daniele, V Ciriello, F Rosato, A Garnero, L Leotta, M Giacometti, S Zonta, D Lomiento, L Taglietti, S Dester, B Compagnoni, F Viotti, R Cazzaniga, R Del Giudice, F Mazzotti, F Pasini, G Ugolini, N Fabbri, C V Feo, E Righini, S Gennari, M Chiozza, G Anania, A Urbani, M Koleva Radica, P Carcoforo, M Portinari, M Sibilla, A Anastasi, B Bartalucci, A Bellacci, G Canonico, L Capezzuoli, C Di Martino, P Ipponi, C Linari, M Montelatici, T Nelli, G Spagni, L Tirloni, A Vitali, C Agostini, G Alemanno, I Bartolini, C Bergamini, A Bruscino, C Checcucci, R De Vincenti, A Di Bella, M Fambrini, L Fortuna, G Maltinti, P Muiesan, F Petraglia, P Prosperi, M N Ringressi, M Risaliti, F Sorbi, A Taddei, V Lizzi, F Vovola, A Arminio, A Cotoia, A L Sarni, P Familiari, G D'Andrea, V Picotti, F Bàmbina, T Fontana, F Barra, S Ferrero, C Gustavino, C Kratochwila, A Ferraiolo, S Costantini, P Batistotti, A Aprile, C Almondo, L Ball, C Robba, S Scabini, D Pertile, A Massobrio, D Soriero, S D'Ugo, N Depalma, M G Spampinato, L Lippa, C Gambacciani, O S Santonocito, F Aquila, F Pieri, M Ballabio, P Bisagni, M Longhi, T Armao, M Madonini, A Gagliano, P Pizzini, A Costanzi, M Confalonieri, M Monteleone, G Colletti, C Frattaruolo, G Mari, A Spinelli, G Mercante, G Spriano, F Gaino, F Ferreli, A De Virgilio, V Rossi, M M Carvello, F Di Candido, H Kurihara, E Marrano, G Torzilli, C Castoro, F M Carrano, F Martinelli, A Macchi, M Fiore, S Pasquali, S P B Cioffi, M Baia, C Abatini, C Sarre, A Mosca, D Biasoni, A Gronchi, D Citterio, V Mazzaferro, P Cadenelli, M Gennaro, V Capizzi, M Guaglio, L Sorrentino, G Bogani, G Sarpietro, L Giannini, L V Comini, L Rolli, S Folli, F Raspagliesi, C Piazza, M Cosimelli, R Salvioni, B Antonelli, L Baldari, L Boni, E Cassinotti, L Pignataro, G Rossi, S Torretta, G A Beltramini, A Gianni', M Tagliabue, R De Berardinis, G Pietrobon, F Chu, S Cenciarelli, L Adamoli, M Ansarin, U Fumagalli Romario, F Mastrilli, N M Mariani, V Nicastro, P Cellerino, F Colombo, A Frontali, A Bondurri, C Guerci, A Maffioli, L Ferrario, M Candiani, G Bonavina, J Ottolina, L Valsecchi, P Mortini, F Gagliardi, M Piloni, M Medone, G Negri, A Bandiera, P De Nardi, P Sileri, M Carlucci, D Pelaggi, R Rosati, A Vignali, P Parise, U Elmore, N Tamini, L C Nespoli, M Rennis, L Pitoni, M F Chiappetta, E Vico, R Fruscio, T Grassi, D Sasia, M Migliore, A Gattolin, R Rimonda, E Travaglio, E Olearo, A Tufo, E Marra, P Maida, G Marte, P Tammaro, F Bianco, P Incollingo, F Izzo, A Belli, R Patrone, V Albino, M Leongito, V Granata, M Piccirillo, R Palaia, E Francone, S Gentilli, H Nikaj, A Fiorini, C Norcini, A Chessa, M V Marino, A Mirabella, G Vaccarella, L Musini, L Ampollini, M Bergonzani, A Varazzani, L Bellanti, M Domenichini, G Rossi, E Cabrini, A Fornasari, A Freyrie, D O Dejana, G D'Angelo, G Bertoli, F Di Lella, G Bocchialini, M Falcioni, D Lanfranco, T Poli, M Giuffrida, A Annicchiarico, G Perrone, F Catena, A Raffaele, A De Manzoni Garberini, E Baldini, L Conti, M Ribolla, P Capelli, S M Isolani, P Maniscalco, M Cauteruccio, C Ciatti, C Puma Pagliarello, S Gattoni, R Galleano, M Malerba, M Ciciliot, F Farnesi, M Calabrò, N S Pipitone Federico, E G Lunghi, A Muratore, L Morelli, G Di Franco, M Palmeri, D Tartaglia, F Coccolini, M Chiarugi, T Simoncini, A Gadducci, M Caretto, A Giannini, A Perutelli, L Domenici, S Garibaldi, R Capanna, L Andreani, N Furbetta, S Guadagni, M Bianchini, D Gianardi, E Pinotti, M Montuori, F Carissimi, G Baronio, M Zizzo, C Castro Ruiz, V Annessi, M T Montella, G Falco, S Mele, G Ferrari, V Mastrofilippo, V D Mandato, L Aguzzoli, C Corbellini, C Baldi, G M Sampietro, G M Palini, N Zanini, G Garulli, R Barone, A Murgese, S Mungo, M Grasso, C Marafante, S L Birolo, E Moggia, M Caccetta, A Masciandaro, A Deirino, M Garino, L Gordini, C P Lombardi, F Marzi, A A Marra, C Ratto, M Di Muro, F Litta, V De Simone, V Cozza, F Rosa, A Agnes, A Parello, S Alfieri, G Sganga, P Lapolla, A Mingoli, G De Toma, E Fiori, F La Torre, P Sapienza, G Brachini, B Cirillo, I Iannone, M Zambon, A Chiappini, S Meneghini, G B Fonsi, P M Cicerchia, P Bruzzaniti, A Santoro, A Frati, G Marruzzo, D Ribuffo, A Sagnotta, L Marino Cosentino, S Mancini, G Lisi, D Spoletini, V Bellato, M Campanelli, G Sica, L Siragusa, L Bonavina, E Asti, D Bernardi, A Lovece, T Perra, A Porcu, A Fancellu, C F Feo, A M Scanu, F Tuminello, R Galleano, A Franceschi, A Langone, F Fleres, A Spolini, P Bordoni, M Franzini, G Clarizia, A Grechi, A Longhini, U Grossi, S Novello, G Zanus, M Romano, S Rossi, G Pirozzolo, A Recordare, S Paiella, G Turri, S Rattizzato, T Campagnaro, A Guglielmi, C Pedrazzani, A Ruzzenente, E Poletto, S Conci, L Casetti, M Fontana, R Salvia, G Malleo, A Esposito, L Landoni, M De Pastena, C Bassi, M Tuveri, S Nobile, G Marchegiani, L Bortolasi, E Sambugaro, M Malavolta, G Moretto, H Impellizzeri, M Inama, G Barugola, F Ascari, G Ruffo, S Granieri, C Cotsoglou, M Berselli, M Desio, V Marchionini, E Cocozza, S Di Saverio, G Ietto, D Iovino, G Carcano, F Ayasra, A Qasem, Y Ayasra, M Al-Masri, M K Abou Chaar, H Al-Najjar, K Ghandour, F Alawneh, R Abdel Jalil, S Abdel Al, M Elayyan, R Ghanem, I Lataifeh, O Alsaraireh, F J Abu Za’nouneh, T Fahmawee, A Ibrahim, K Obeidat, I Albader, J Alabbad, M A S Albader, A Bouhuwaish, A S Taher, M S M Omar, E Abdulwahed, M Biala, M Morgom, Elhadi A, Alarabi A, A Msherghi, Elhajdawe F, A Alsoufi, A Salamah, H Salama, M Bulugma, H Almabrouk, D Venskutonis, E Dainius, E Kubiliute, S Bradulskis, A Parseliunas, J Kutkevicius, A Subocius, Y J Cheong, M S Masood, C W Ngo, R Saravanan, N Abdul Maei, G Yanowsky Reyes, J Orozco Perez, R Damian, R Santana Ortiz, C A Colunga Tinajero, F Cordera, A Gómez-Pedraza, A Maffuz-Aziz, J A Posada, M A De la Rosa Abaroa, M R Alvarez, R Arrangoiz, R Hernández, K Bozada Gutierrez, M Trejo-Avila, C Valenzuela-Salazar, J Herrera-Esquivel, M Moreno-Portillo, V M Pinto-Angulo, E E Sosa-Duran, H Ziad-Aboharp, X Jimenez Villanueva, C E Soulé Martínez, A I Lupián-Angulo, J J Martínez Zarate, E Reyes Rodriguez, G Montalvo Dominguez, F C Becerra García, J Melchor-Ruan, D Vilar-Compte, E Romero-Bañuelos, A Herrera-Gomez, A Meneses-Garcia, D Isla-Ortiz, R A Salcedo-Hernández, J M Hernández-Nava, J E Morales-Castelan, C Sarre, O E Posadas-Trujillo, G A Buerba, A Alfaro-Goldaracena, E Pena Gomez-Portugal, G Lopez-Pena, C A Hinojosa, M A Mercado, A Ramos-De la Medina, L Martinez, I Duran, D S Gonzalez, M J Martinez, A Nayen Sainz de la Fuente, L Miguelena, L Hernández Miguelena, S M Louraoui, A El Azhari, M Rghioui, E Khya, A Ghannam, A Souadka, B El Ahmadi, Z H Belkhadir, M A Majbar, A Benkabbou, R Mohsine, M Y Oudrhiri, H Bechri, Y Arkha, A El Ouahabi, H Frima, S Bachiri, L C Groen, T Verhagen, F M ter Brugge, J C G Scheijmans, M A Boermeester, R Hompes, E M Meima-van Praag, S Sharabiany, A B J Borgstein, S S Gisbertz, M I van Berge Henegouwen, S Gans, P Van Duijvendijk, T Herklots, T De Hoop, M R De Graaff, D Sloothaak, M Bolster-van Eenennaam, J Baaij, J H G Klinkenbijl, R Van Eekeren, E J Spillenaar Bilgen, S W Van der Burg, N J Harlaar, F H W Jonker, M Vermaas, K R Voigt, D Nellensteijn, E A B Bensi, L Posma-Bouman, M Van Sambeek, M Holscher, W T Van den den Broek, S H Kruijff, J P P M De Vries, P J Steinkamp, P K C Jonker, W Y Van der Plas, W Bierman, Y Janssen, J Franken, S Oosterling, E G Boerma, D Schweitzer, M H F Keulen, S Ketting, J A Wegdam, T S de Vries Reilingh, E Schipper, P H E Teeuwen, E R Hendriks, A A W Van Geloven, M Emous, R Poelstra, M Teunissen, S L Gerritsen, D Boerma, P R De Reuver, F Thunnissen, B A M Vermeulen, A Groen, T M Van Ginhoven, C L Viëtor, M J W van der Oest, P W H E Vriens, T Houwen, J Heisterkamp, A S van Petersen, W van der Meij, C T Stevens, A Pronk, W J Bakker, M C Richir, M R Vriens, M D Filipe, M Uittenbogaart, W K G Leclercq, J M L Sijmons, P J Vancoillie, J Konsten, M Van Heinsbergen, N A M Dekker, F C den Boer, A Akinmade, A Adeyeye, E Enoch, S Fayose, A I Okunlola, A A Adeniyi, O T Adeyemo, I O Adebara, A Bakare, O F Babalola, O H Abiyere, O O Banjo, S Olori, O G Akaba, E T Agida, I H Abdullahi, I K Egbuchulem, D Olulana, T A Lawal, O Ogundoyin, O A Oyelakin, O G Nwaorgu, S O Sule, C C Makwe, B B Afolabi, J O Seyi-Olajide, A O Ademuyiwa, C O Bode, O Atoyebi, O A Elebute, A A Okunowo, O M Williams, N G Eke, O A Oshodi, O M Faboya, A S Adeniran, O A Omisanjo, Y A Oshodi, A A Ogunyemi, K M Atobatele, A Adeyeye, I Aremu, O Olasehinde, L Abdur-Rahman, J Bello, A Popoola, T O Sayomi, H O Raji, N Adeleke, B Lawal, O Habeeb, O Agodirin, M A Tolani, T T Sholadoye, S E Nwabuoku, M Abubakar, T Risteski, V Cvetanovska Naunova, L Jovcheski, E Lazova, I Agledahl, R G Breuer, J Massoud, S H Waqar, I Rashid, A Ayubi, A B H Bhatti, M U Younis, A Ghouri, B Ayub, R H Sayyed, A Saleem, K Turk, A Alvi, J Abassy, S Khan, M Arshad, K Ahmed, T Siddiqui, A Pirzada, A A Kerawala, A Jamal, L Rai, R Nafees Ahmed, A S Memon, A U Qureshi, M Ayyaz, M Umar, U Butt, M Kashif, W H Khan, M Waris Farooka, T Wasim, N Talat, W Tahir, J Naseem, A Akbar, S Afroze, L Ali, A Sultan, H B Ali, M H Janjua, A Janjua, S Asghar, M S Farooq, M Z Sarwar, S A Naqi, K M Gondal, S I Bukhari, M Tariq, S Javed, E Yaqoob, M Ashraf, U Mahmood, K Raja Shabbir, S A Abukhalaf, A Amro, J M Cabada Lee, A Aguilar, E Rodriguez, K Castillo, M Cukier, H Rodriguez-Zentner, E Arrue, R Isaacs Beron, A Rodríguez Gonzalez, V Panduro-Correa, D K Cornelio, C E Otiniano Alvarado, V D Caballero Sarabia, X P Vasquez-Ojeda, G Lizzetti-Mendoza, M Niquen-Jimenez, S B Shu-Yip, J L León Palacios, G Borda-Luque, S A Zegarra, E Huamán Egoávil, C Suazo Carmelo, R Castro de la Mata, D Rivas, J Targarona, Y Trujillo, M Olivera Villanueva, A Lahoud-Velaochaga, K Cabillas, W Castañeda, J Colina Casas, J Betalleluz Pallardel, F Camacho Zacarías, E Vélez Segura, D L Cruz Condori, E Huamán, R Ugarte Oscco, C Vergel Cabrera, E Huamán Egoávil, Y T Carpio Colmenares, L A García Barrionuevo, D Cárdenas Ruiz de Castilla, P Mansilla Doria, M R Li Valencia, A Salazar, A Sarmiento, C Díaz, E Morales, E Ore, H Zegarra, J Siccha, M Guardia, M Sandoval, G C Mendiola, M Mimbela, R Diaz-Ruiz, L A Zeta, E Cordova-Calle, H M Nuñez, M R Ortiz-Argomedo, J Caballero-Alvarado, A Salazar-Tantaleán, R Espinoza-Llerena, M Aliaga-Ramos, O Asodisen, E Jabagat, C M Tedoy, R A Ramos, M P J Lopez, K L E Violago, R Aram, P Carlos Santos, R F Filarca, A Carlos, P Santos, R L Filarca, E J Domingo, K J O Khu, M C Lapitan, M D P Sacdalan, M J N Kho, R E Baticulon, S L R Bravo, M A C Cueto, C L Ramos, J R Fuentes, H Sadian, A Gumarao, A Barraquio, E M Cruz, A D Gonzales, J A S Reyes, J A Salud, E G Tancinco, R D Rivera, J A Lim, J C Barcelon, J A Chiu, M I Carballo, P Major, I Gawron, R Jach, F Borges, P Matos Costa, S Henriques, S C Rodrigues, N Gonçalves, A Cabeleira, C Branco, P Serralheiro, R Alves, T Teles, A Lázaro, C Canhoto, J Simões, M Costa, A C Almeida, O Nogueira, A Oliveira, R Athayde Nemésio, M Silva, C Lopes, M J Amaral, A Valente da Costa, R Andrade, R Martins, A Guimarães, P Guerreiro, A Ruivo, C Camacho, M Duque, E Santos, D Breda, J M Oliveira, A L De Oliveira Lopez, S Garrido, M Colino, J De Barros, S Correia, M Rodrigues, P Cardoso, R Martins, J Teixeira, A P Soares, H Morais, R Pereira, T Revez, M I Manso, J C Domingues, P Henriques, R Ribeiro, V I Ribeiro, N Cardoso, S Sousa, G Martins dos Santos, L Carvalho, C Osório, J Antunes, S Lourenço, P Balau, M Godinho, A Pereira, N Silva, A Kam da Silva Andrade, A Pereira Rodrigues, N Borges, J Correia, I Vieira, T Ribeiro, J Catarino, R Correia, F Pais, R Carreira Garcia, R Bento, J Cardoso, M Luis, E Santos, J Henriques, J Patena Forte, J Maciel, J Pinheiro Santos, M Silva, T P Silva, A Branquinho, A Caiado, P Miranda, R Garrido, M Peralta Ferreira, J Ascensão, B Costeira, C Cunha, L Rio Rodrigues, M Sousa Fernandes, P Azevedo, J Ribeiro, I Lourenço, H Gomes, G Mendinhos, A Nobre Pinto, A Ribeiro, C G Gil, C Lima-da-Silva, C Pereira, F Tavares, I Ferraz, J I Almeida, J Marialva, L Lopes, M J M A Costa, M Nunes-Coelho, M J Teixeira, N Machado, J P Alfonso, P Saraiva, R L Silva, R Santos, R Almeida-Reis, T Correia-de-Sá, V Fernandes, J Almeida-Pinto, J P Gonçalves, H Santos-Sousa, S Cavaleiro, A M Leite-Moreira, A Pereira, A Pereira-Neves, C S Faria, J M Monteiro, J Nogueiro, M Sampaio-Alves, M Magalhães Maia, P Vieira, T Pina-Vaz, F Jácome, V Devezas, A Almeida, H Silveira, S Vaz, S Castanheira Rodrigues, D Costa Santos, J V Grilo, A Abreu da Silva, M Claro, A C Deus, R Branquinho, P M D D Santos, B Patrício, A C Vieira Paiva Lopes, J M Mendes, M F Carvalho, C M Oliveira, A Tojal, J Pinto, A Abutaka, A Zarour, M Abdelkareem, S M Ali, M Al Tarakji, R Alfkey, K Mukhtar, I R Wani, R Singh, K Ahmed, N Bouchiba, H Mahdi, S Abdelaziem Mustafa, A Al Ansari, R Drasovean, A Caziuc, E Galliamov, M Agapov, V Kakotkin, E Semina, XXX Kubyshkin, A Kamalov, S K Efetov, V S Kochetkov, T Garmanova, P Tsarkov, I Tulina, S Rodimov, D Markaryan, E Kazachenko, A Yanishev, A Abelevich, A Bazaev, A Kokobelyan, P Zarubenko, A Zakharenko, A Novikova, G Kim, D Shmatov, M Stoliarov, M Kamenskikh, G Nambi, A S Almulhim, T Madkhali, A Alzouhir, A Alissa, E Alameer, M Badedi, A Q Alnami, H Darraj, A Alkhuzaie, F Khadwardi, M Abualjadayel, W Tashkandi, A Farsi, N Malibary, N Trabulsi, S Farsi, A Said Bayazeed, M Nasser, M S Siddiqui, S Al Awwad, A Gudal, A Alasmari, S Alqahtani, S Majrashi, A Mashat, R Al Raddadi, A Alharbi, Y Nasser, H Hamayel, A Alhojaili, R Aljohani, O Sogair, O Alfarhan, A Alzahrani, B Alzomaili, W Tashkandi, M A Azab, M Alotaibi, A Maashi, A Zowgar, M Alsakkaf, M Alnemary, S Khayat, S Felmban, A Almhmadi, M Alqannas, D Cortés Guiral, M Alyami, A Elawad, A Alhefdhi, F Alresaini, W Kurdi, M Tulbah, M Aldakheel, N Alsahan, S Koussayer, H Elsheikh, M Al-qattan, S Alshanafey, A Rafique, N Mahabbat, B Saeed, E Al-Kharashi, K Alsowaina, N Arab, F Aljaber, I Al Hasan, A Alghamdi, F Badahdah, A Alghuliga, F Abdulfattah, F Alanazi, F Albaqami, A Alsuhaibani, A AlFakhri, S Alqasem, N Alajaji, T Nouh, A Bin Nasser, J Alowais, A Alburakan, O Alamri, A Albdah, K Alawi, M Alshalhoub, K ElSanhoury, A Almofarreh, S Ibrahim, H Elshafie, I Osman, T Guzman, H Mutair, A Siddiqui, S Chowdhury, R Alghamdi, S Almutrafi, J Alfaifi, J D'Souza, A Alshitwi, N Alkreedees, M Alramadhan, M Alshehri, A Alzahrani, S Alobaysi, H Badr, A Alshahrani, A Alshehri, M Alrashed, T Altahan, T Alsabahi, R Alhossaini, M Sbaih, Y Alalawi, K Alnwijy, A Al Ayed, S Ghedan, R Alharthi, S Awad, M Sharara, S Abdelrhman, W Althobaiti, L Srbinovic, M Perovic, Z Mikovic, B Nikolic, M Vasiljevic, V Pazin, V Mandic Markovic, D Dimitrijevic, N Zecevic, P Gregoric, D Micic, Z Loncar, K Doklestic, N Ivancevic, V Djukic, D Stojakov, R Ilic, P Savic, N Pijanovic, M Milanovic, M Radosavljevic, T Dejanovic, M Kostic, J Paskas, S Bojic, P Stevanovic, M Djuric, M Kadija, G Tulic, I Glisovic Jovanovic, B Lieske, E Kayombo, I Kruger, M De Kock, A Malan, C Ferreira, H Du Preez, W Mulder, C Noel, S Le Grange, O Lusawana, C Kies, E Steyn, J Janson, J J P Buitendag, K Chu, M Mihalik, R Nel, S Naidoo, C Kloppers, D Nel, E Jonas, H Pickard, M Bernon, N Almgla, S Rayamajhi, W Mugla, G Y Hyman, M Fourtounas, R Moore, A Sánchez Mozo, H Aguado Lopez, A Zárate Pinedo, M Jimenez Toscano, N Alonso de la Fuente, G Mancebo, L Cecchini, M Munarriz, M Cazador Labat, A López Campillo, P Martorell, C A Espinosa, P Caja Vivancos, I Villalabeitia Ateca, M Prieto Calvo, H Marín, P Martin Playa, A Gainza, E J Aragon Achig, A Rodriguez Fraga, I Melchor Corcóstegui, G Mallabiabarrena Ormaechea, J J Garcia Gutierrez, L Barbier, M A Pesántez Peralta, M Jiménez Jiménez, J A Municio Martín, J Gómez Suárez, G García Operé, L A Pascua Gómez, M Oñate Aguirre, A Fernandez-Colorado, M De la Rosa-Estadella, A Gasulla-Rodriguez, M Serrano-Martin, A Peig-Font, S Junca-Marti, M Juarez-Pomes, S Garrido-Ondono, L Blasco-Torres, M Molina-Corbacho, Y Maldonado-Sotoca, A Gasset-Teixidor, J Blasco-Moreu, L Gomez Fernandez, L Cayetano Paniagua, O Izquierdo, D Ventura, J Castellanos, E Ballester Vazquez, A Sanchez Lopez, C Balague Ponz, E M Targarona Soler, S Sanchez Cabús, V Molina Santos, J A Gonzalez Lopez, R Medrano Caviedes, A Moral Duarte, E Espin-Basany, G Pellino, R Blanco-Colino, V Turrado-Rodriguez, A M Lacy, F B de Lacy, X Morales, A Carreras-Castañer, P Torner, M Jornet-Gibert, M Balaguer-Castro, M Renau-Cerrillo, P Camacho-Carrasco, M Vives-Barquiel, B Campuzano-Bitterling, I Gracia, R Pujol-Muncunill, O Martin-Sole, J Rubio-Palau, X Tarrado, L Garcia-Aparicio, I De Haro Jorge, A Martin, J Rojas-Ticona, S Perez-Bertolez, M Cuesta Argos, B Capdevila Vilaro, M Coronas Soucheiron, M Riba Martinez, L Saura Garcia, J Prat Ortells, M Bejarano Serrano, P Parri, C Massaguer, F Vicario, P Palazon Bellver, I Moraleda Gudayol, A Lara, D Escobar, M Arrieta, U Garcia de Cortazar, I Villamor Garcia, A Landaluce-Olavarria, M Gonzalez De Miguel, L Fernández Gómez Cruzado, E Begoña, D Lecumberri, M A Acosta Mérida, A F Yepes Cano, M Estaire Gómez, D Padilla-Valverde, S Sánchez-García, D Sanchez-Pelaez, E Jimenez Higuera, R Picón Rodríguez, à Fernández Camuñas, C Martínez-Pinedo, E P Garcia Santos, V Muñoz-Atienza, A Moreno Pérez, C A López de la Manzanara Cano, B Ugarte-Sierra, F J Ibáñez-Aguirre, U De Andres Olabarria, F J Fernández Pablos, M Durán Ballesteros, A Sanz Larrainzar, V Jiménez Carneros, A Valle Rubio, L Alonso-Lamberti, A Salazar, J García-Quijada, R Leon, J L Rodriguez, J Jimenez Miramón, J M Jover, M B Martín Salamanca, M Assaf, V Pérez Simón, S A Landeo Agüero, N Baeza Pintado, M A Huertas Fernandez, A Carabias, M V Sosa, P Lora-Cumplido, L Lanuza, M Galipienso Eri, J D Garcia Montesino, J Dellonder Frigolé, D Noriego Muñoz, A Navarro-Sánchez, D Enjuto, M Perez Gonzalez, P Díaz Peña, J Gonzalez, M Marqueta De Salas, P Martinez Pascual, L Rodríguez Gómez, R Garcés García, A Ramos Bonilla, N Herrera-Merino, P Fernández Bernabé, E P Cagigal Ortega, I Hernández, E García de Castro Rubio, I Cervera, M Espino Segura-Illa, G Sánchez Aniceto, A M Castaño-Leon, L Jimenez-Roldan, J Delgado Fernandez, A Pérez Núñez, A Lagares, D Garcia Perez, M Santas, I Paredes, O Esteban Sinovas, L Moreno-Gomez, E Rubio, V Vega, A Vivas Lopez, M Labalde Martinez, O García Villar, P M Pelaéz Torres, J Garcia-Borda, E Ferrero Herrero, P Gomez, C Eiriz Fernandez, C Ojeda-Thies, J M Pardo Garcia, M Di Martino, A De la Hoz Rodriguez, J García Septiem, R Maqueda González, L Delgado Búrdalo, A Correa Bonito, E Martin-Perez, J E García Villayzán, B Albi Martin, P Lozano Lominchar, L Martin, M Fernadez, C Rey-Valcarcel, M Tousidonis, L Martin-Albo Caballero, A Lowy, P Alonso Ortuño, E Ayuso Herrera, J Caño Velasco, J Aragon-Chamizo, M D Perez Diaz, O Mateo-Sierra, B Quintana-Villamandos, J M Barrio, M Fanjul, C Sanchez-Perez, M L Fernandez, S Hernandez-Kakauridze, J Rio, D Díaz Pérez, J Serrano González, L Colao García, M Gutierrez Samaniego, M A Hernandez Bartolome, P Galindo Jara, E Esteban Agustí, J Ripollés-Melchor, A Abad-Motos, A Abad-Gurumeta, E Martínez-Hurtado, A Ruiz-Escobar, N Brogly, E Guasch, A Hernandez Gutierrez, J L Bartha Rasero, Y Perez, V Garcia-Pineda, M Gracia, J Siegrist Ridruejo, M D Diestro, J I Sanchez-Mendez, C Marti, M Melendez, E Moreno-Palacios, A Loayza, L Frias, I Zapardiel, I Rubio-Perez, M I Prieto Nieto, J Guevara, A Gegundez Simon, S Gortazar, N Chavarrias, E Alvarez, J Saavedra, P Ramos-Martin, A Urbieta, J Gomez Rivas, C Toribio-Vázquez, A Yebes, M Hernández-García, M Losada, B Diéguez, M García-Conde, A Alonso Poza, L Marquez, R Becerra, M Martin, T Jorgensen, J M Muguerza, J Dziakova, C Sánchez del Pueblo, P Saez Carlin, E Camarero, S Picazo, M J Pizarro, R Avellana, V Catalán, L Lopez Antoñanzas, O Cano, R Anula, R Sanz Lopez, G Sanz Ortega, M Garcia Alonso, A J Torres, E Martin Antona, S Garcia Botella, D Ramos, A G Barranquero, J Ocaña, J Núñez, C Cerro Zaballos, D Crego-Vita, M Huecas-Martinez, M Diez Alonso, F Mendoza-Moreno, C Vera Mansilla, E Ovejero Merino, P Hernandez, A Blazquez Martin, F Ruiz Grande, N Morales Palacios, E Garcia-Loarte Gomez, E Garca Rico, A M Minaya-Bravo, C San Miguel Méndez, A Galvan Pérez, E Gonzalez-Gonzalez, A Robin Valle de Lersundi, E Calcerrada Alises, M A García-Ureña, A Cruz Cidoncha, P Troncoso Pereira, F Alcaide Matas, J M García Pérez, J M Muñoz Vives, A Osorio, C J Gómez Díaz, C A Guariglia, C Soto Montesinos, L Sanchon, M Xicola Martínez, N Guàrdia, P Collera, R Diaz Del Gobbo, R Sanchez Jimenez, R Farre Font, R Flores Clotet, P Calvo Espino, P Guillamot Ruano, J Rey-Biel, L Pingarrón-Martin, I Ruiz Martin, C Moliner Sanchez, M Carrasco-Prats, C Giménez-Francés, M Ruiz-Marín, A J Fernández-López, D García-Escudero, V García-Porcel, R Lax-Pérez, M Sánchez-Robles, M Valero-Soriano, E Medina-Manuel, V García-Soria, E Gurrea-Almela, A Marco-Garrido, J A Martínez-Alonso, F M González-Valverde, P V Fernández-Fernández, C Sánchez-Rodríguez, J Aguilar-Jimenez, M Baeza-Murcia, J L Aguayo-Albasini, T Nicolás-López, F Alconchel, D Fernández Martínez, L Solar-Garcia, L J García Flórez, H Llaquet Bayo, N Pujol-Cano, J J Segura-Sampedro, C Soldevila-Verdeguer, S Jeri-McFarlane, A Gil-Catalan, A Craus-Miguel, L Cruz, P Valente, M Afonso-Garcia, E Ferrer-Inaebnit, A Oseira-Reigosa, L Fernandez-Vega, B Villalonga-Ramirez, F X Gonzalez Argente, I Mora-Guzmán, F J Landete Molina, F J Morera Ocón, E Canelles Corell, M T Gavaldà Pellicé, J R Salinas Peña, P Cavallé Busquets, J Trebol, A B Sánchez-Casado, L Munoz-Bellvis, L E Pérez-Sánchez, V Concepción Martín, A Díaz García, M Vallve-Bernal, A Calvo Rey, G M Prada Hervella, L Dos Santos Carregal, M I Rodriguez Fernandez, M Freijeiro, S El Drubi Vega, A L Picardo, A Cuadrado-Garcia, D Serralta de Colsa, J A Rojo Lopez, F Sanchez Cabezudo Noguera, I Ortega Vazquez, L Garcia-Sancho Tellez, P Mato, J Heras Aznar, J Jimeno Fraile, D Morales-Garcia, M Carrillo-Rivas, E Toledo Martínez, à Pascual, A Senent-Boza, A Sánchez-Arteaga, I Benítez-Linero, F Manresa-Manresa, L Tallón-Aguilar, L Melero-Cortés, M R Fernández-Marín, V M Durán-Muñoz-Cruzado, I Ramallo-Solís, P Beltrán-Miranda, F Pareja-Ciuró, B T Antón-Eguía, F Oliva Mompean, J Gomez-Rosado, J Reguera-Rosal, J Valdes-Hernandez, L Capitan-Morales, M D del Toro Lopez, M Achalandabaso Boira, R Memba Ikuga, M Abellán, R Sales, C Olona, R Jorba, J Hernandez Gutierrez, A Tébar Zamora, J Sancho-Muriel, H Cholewa, M Frasson, J Domenech, A Roselló Añón, M J Sangüesa, D Moro-Valdezate, M Garcés-Albir, F Lopez, J C Bernal-Sprekelsen, J C Catalá Bauset, P Renovell Ferrer, C Martínez Pérez, O Gil-Albarova, J Gilabert Estellés, K Aghababyan, B De Andrés-Asenjo, J Beltrán de Heredia, A Vázquez-Fernández, F J Ortiz de Solorzano-Aurusa, J Trujillo-Díaz, M Ruiz-Soriano, C Jezieniecki, T Gómez-Sanz, H Núñez-Del Barrio, A Romero-De Diego, V García-Virto, H J Aguado, M T Fernández Martín, F J Tejero-Pintor, B Pérez-Saborido, E Choolani Bhojwani, F Acebes García, P Marcos-Santos, AD Bueno Cañones, J Sanchez Gonzalez, M Toledano, M Bailón, D Pacheco Sánchez, M Paniagua Garcia Senorans, R Sanchez-Santos, A Vazquez Melero, D Garcia, E Díez, I Herrero, I M Soeda, M Camuera, M Balluerca, M Sánchez-Rubio, P Paunero Vazquez, A Martinez-German, C Gracia-Roche, I Gascon-Ferrer, V Duque-Mallen, M D C De Miguel-Ardevines, N Sanchez-Fuentes, M S Santero-Ramirez, M Matute-Najarro, M Herrero-Lopez, M Sanchez-Rubio, M Cantalejo-Diaz, M T Gonzalez-Nicolas-Trebol, S Saudi-Moro, U M Jariod-Ferrer, R Rivas, F Rivas, J Escartin, J L Blas Laina, A Nogués, B Cros, I Talal El-Abur, J Garcia Egea, C Yanez, U Jayarajah, S Ravindrakumar, V S D Rodrigo, A Arulanantham, G B K D Bandara, H K S Hamid, E E Ali, A B H Widatalla, I Bakheit, M Awadelkarim, A A Ali Karar, M Saleh, H Taflin, P Myrelid, L Amorim Braz, L Hagander, M Hambraeus, E Omling, M SalÖ, S Arkani, J Freedman, C Montan, E K Lindqvist, P Elbe, R Hultgren, A Älgå, M Nordberg, G Sandblom, M Rutegård, F Holmner, M Sund, N LÖfgren, A Tampakis, O Kollmar, M von Fluee, A Balaphas, C Toso, N Colucci, S G Popeskou, M Gass, A Scheiwiller, J Metzger, E Gialamas, M Chevallay, M Sauvain, O Dwidar, S Y Kiessling, S J Stoeckli, F Mongelli, M Bernasconi, M Di Giuseppe, D Christoforidis, D La Regina, M Arigoni, M Adamina, G Peros, L Guglielmetti, F Solimene, M Giardini, T BÄchler, A S Crugnale, C A Gutschow, M Turina, O Ersen, M A Onan, R Kozan, T Erol, H A Dincer, A Yildiz, N Iflazoglu, O Yalkın, A Isik, V Ozben, E Aytac, Z Aliyeva, E Akaydin, B B Ozmen, B Baca, Y Altinel, F Calikoglu, M Tokocin, N A Hacim, A Akbas, S Meric, T Vartanoglu, H Yigitbas, C Ercetin, G Ercan, I Ozgur, M Keskin, K T Saracoglu, B Cimenoglu, R Demirhan, A Kale, T Simsek, E C Gundogdu, A Abbasov, M Tanal, B Citgez, E Bozkurt, S G Yetkin, M Mihmanli, A Alhamed, S Ergun, A N Sanli, M Velidedeoglu, M F OZcelik, S S Uludag, A K Zengin, S Cebi, F Demirkiran, T Bese, A S Acikgoz, B Kayan, Y Aykanat, D Mutlu, B GÖksoy, Y Kara, M A Bozkurt, A Kocatas, H Öğücü, G Uslu, C Arican, C Tugmen, C Aydin, D Yesilyurt, E K Avci, E Kebapçı, G Kilinc, İ Sert, K Tuncer, M Akalin, M Emiroglu, S Demirli Atici, S Salimoğlu, T Kaya, Y Kirmizi, O C Tatar, E Yüksel, S A Güler, A Yildirim, N Z Utkan, K GÖzal, H KÖken, A Yabas, E Gonullu, F Altintoprak, E Akin, B Kamburoglu, R Capoglu, F Kucuk, H Demir, G Cakmak, N Firat, F Celebi, B Kocer, B Mantoglu, Z Bayhan, E Dikicier, E Colak, G O Kucuk, E Karaman, A Kolusab, O Karaaslan, i Majid, S Alshryda, F Abbas, F M A Abbas, D Mohammed, M A Tahlak, A Yammahi, A A Albaroudi, B Elyafawi, A Saber, H Khansaheb, H Alsaadi, N Alzarooni, M Bekheit, B S Kamera, M Elhusseini, P Sharma, A Ahmeidat, G Gradinariu, W Cymes, A Hannah, G Mignot, S Shaikh, J Agilinko, D Angelou, D Neely, A McCanny, B McAree, A J Baldwin, R West, E Gammeri, A Catton, S Marinos Kouris, J Pereca, J Singh, Z Seymour, R Jones, S Leeson, R Peevor, A K Lala, C Houlden, J Kahiu, N Hossain, S Hosny, P Patel, S Handa, M Kaushal, A Kler, V Reghuram, S Tezas, K Fairhurst, C Yates, S Mitchell, J Bunni, S Richards, R George, S M Lee, J Phull, J Frost, S Burnard, R Crowley, A Airey, K Bevan, R Makin-Taylor, C S Ong, R Callan, O Bloom, F Aljanadi, N Moawad, M Jones, A Gregg, R Jeganathan, M Pachl, B Martin, J E Archer, A Odeh, N Siddaiah, R Singhal, D N Naumann, S Karandikar, A Syed, O N Tucker, R Alam, M Kalkat, J K C Mak, R Kulkarni, N Sharma, P Nankivell, F Tirotta, A Parente, O Breik, A Kisiel, L D Cato, S Saeed, A Bhangu, E Griffiths, A M Pathanki, S Ford, A Desai, M Almond, M Kamal, S Sundar, E Y L Leung, R Kaur, C Brett-Miller, F E Buruiana, G Markose, A De Gea Rico, A Taib, D Myatt, A Sulaiman Khaled, F Younis, A Sultana, M Taggarsi, L Vitone, J Lambert, O P Vaz, i Sarantitis, D Shrestha, S Timbrell, A Shugaba, B Quddus, J Law, M N Bittar, M Creanga, M Elniel, M Youssef, S Ali, S T Qadri, G Brixton, L Findlay, T Klatte, A Majkowska, J Manson, R Potter, V Oktseloglou, F Mosley, M F i De La Cruz Monroy, P Bobak, i Omar, S Ahad, F Langlands, V Brown, M Hashem, L Kennedy, S Jaunoo, E Coomber, O Williams, M Shalaby, H L Rhodes, A Williams, A Ridgway, D Pournaras, E Britton, E Lostis, G K Ambler, H Chu, J Hopkins, J Manara, M Chan, M Doe, R D C Moon, S Lawday, T Jichi, W Singleton, B Main, T Maccabe, C Newton, N S Blencowe, D P Fudulu, D Bhojwani, M Baquedano, M Caputo, F Rapetto, O Flannery, A Hassan, A Coonar, G Aresu, C Smith, D Gearon, J Hogan, i S Pradeep, H Durio Yates, A Peryt, Z M Barrett-Brown, M King, N Ahmadi, D Jenkins, N Moorjani, F Taghavi, F Wells, J Hardie, S Page, F Anazor, S D King, J Luck, S Kazzaz, R Mannion, G D Stewart, J Ramzi, M Mohan, A A Singh, J Ashcroft, O J Baker, P Coughlin, R J Davies, A Z E D Durst, A Abood, A Habeeb, V E Hudson, A Kolias, B Lamb, L Luke, S Mitrasinovic, S Murphy, A W T Ngu, J R O'Neill, S Waseem, K Wong, F Georgiades, P J Hutchinson, X S Tan, J Pushpa-rajah, A Colquhoun, L Masterson, i Abu-Nayla, C Walker, A Balakrishnan, S Rooney, E irune, M H V Byrne, A Durrani, A Simoes, B Eddy, E Streeter, i Ahmed, M Yao, W Wang, A Djouani, J Tait-Bailey, M Thomas, F Hassan, S Kommu, S Chopra, T Richards, A Sethuraman Venkatesan, T Combellack, J Williams, G Tahhan, M Mohammed, M Kornaszewska, V Valtzoglou, i Deglurkar, M Rahman, U Von Oppell, D Mehta, M Koutentakis, S A H Syed Nong Chek, G Hill, C Morris, M Shinkwin, J Torkington, J Cornish, R Houston, S Mannan, F Ayeni, H Tustin, M Bordenave, A Robson, G Dovell, R Preece, P Rolland, B H Miranda, A Sobti, A Khaleel, A Unnithan, K Memon, R R Pala Bhaskar, F Maqboul, F Kamel, A Al-Samaraee, R Madani, L Kumar, P Nisar, S Agrawal, D Vimalachandran, N Manu, N Eardley, E Krishnan, O L Serevina, E Martin, C Smith, A Jones, S Roy Mahapatra, R Clifford, G P Jones, A Gardner, S S Tripathi, M S Greenhalgh, W Matthews, K Mohankumar, i Khawaja, A Palepa, T Doulias, C Gill, N Dunne, D R Sarma, C Godbole, W Carlos, N Tewari, D Jeevan, P Naredla, A Khajuria, H Connolly, S Robertson, C Sweeney, G Di Taranto, S Shanbhag, K Dickson, K McEvoy, J Skillman, M Sait, H Al-Omishy, M Baig, B Heer, A Brown, A Ebrahim, A Alwadiya, A Goyal, A Phillips, A Bhalla, C Demetriou, E Grimley, E Theophilidou, E Ogden, F L Malcolm, G Davies-Jones, J C K Ng, M Mirza, M Hassan, N Elmaleh, P Daliya, S Williams, A Bateman, Z Chia, Y Premakumar, Y Jauhari, Z Koshnow, D Bowen, A Uberai, F Hirri, B M Stubbs, R Crichton, J Sonksen, K Aldridge, C McDonald, J Manickavasagam, K Ragupathy, S Davison, S Dalgleish, N McGrath, R Kanitkar, C J Payne, L Ramsay, C E Ng, T Collier, K Khan, R Evans, C Brennan, D E Henshall, T Drake, E M Harrison, V Zamvar, A Tambyraja, R J E Skipworth, G Linder, R McGregor, P Brennan, J Mayes, L Ross, S Smith, T White, A A B Jamjoom, R Pasricha, K Gallagher, R Swan, H Paterson, Y Maeda, A M F Kwok, A Tsiaousidou, P G Vaughan-Shaw, C Boyle, D Fernando, D Tham, S Leung, A Laird, T Holme, S Abbott, A Razik, S Thrumurthy, J Steinke, M Baker, D Howden, Z Baxter, L Osagie, M Bence, G E Fowler, L Massey, N Rajaretnam, J Evans, J John, A Goubran, N Campain, F D McDermott, J S McGrath, M Ng, J Pascoe, J R A Phillips, i R Daniels, J A'Court, A Konarski, G Faulkner, H Emerson, K Vejsbjerg, L Pearce, W McCormick, A Fisher, K Singisetti, Y Aawsaj, C Barry, O Bajomo, S Rizvi, C Grimes, K Dusu, P Y Tint, A Kirk, V irvine, S Lammy, R O'Kane, L Elliott, G McCabe, D Holroyd, N B Jamieson, A Geddes, J McMahon, J McCaul, M Al-Azzawi, E Aitken, P Glen, L O H Sinan, S Lammy, A Grivas, E J Tilling, O Brown, M Boal, H Dean, S Higgs, S Stanger, H Abdalaziz, J Constable, H ishii, R Preece, G Dovell, R Gopi Reddy, T K Madhuri, A Tailor, M Flavin, D Walker, S Humphries, H Assalaarachchi, T Curl-Roper, E Westwood, C Delimpalta, C C L Liao, V Velchuru, D A Raptis, J M Pollok, N Machairas, B Davidson, G Fusai, F Soggiu, S Xyda, C Hidalgo Salinas, H Tzerbinis, T Pissanou, J Gilliland, S Chowdhury, M Varcada, C Hart, R Mirnezami, J Knowles, N Angamuthu, V Vijay, T Shakir, R Hasan, R Tansey, C Hardie, E Powell-Smith, F Kashora, M H Siddique, A Singh, C Barmpagianni, A Basgaran, A Basha, V Okechukwu, A Bartsch, P Gallagher, A Maqsood, K Sahnan, C A Leo, S E Lewis, H K Ubhi, R Exley, U Khan, P Shah, S Saxena, N Zafar, H Abdul-Jabar, M Patel, A Shabana, A Alanbuki, O Usman, C T Ong, W Butterworth, O Budha Magar, M El Hadi, S Abas, J Annett, E Ross, M Loubani, A Wilkins, H Cao, H Capitelli-McMahon, L Hitchman, H ikram, A Andronic, A Aboelkassem ibrahim, J Totty, J Blanco, R Vanker, M Ghobrial, G Jones, S Kanthasamy, H Fawi, M Awadallah, F Chen, J Cheung, A Moscalu, T Bhuvanakrishna, L Bibby, M Sinclair, M A K Nahid, L Williams, P S Basnyat, A K Shrestha, N K Kumaran, S Sambhwani, N A Sheikh, O M Taylor, i Liew, A Al-Sukaini, S Mediratta, D Saxena, A Sgrò, M M Rashid, K Milne, J Mcintyre, M A Akhtar, A Turnbull, A Brunt, K E Stewart, M S J Wilson, D Rutherford, K McGivern, E Massie, M Ho, R G Wade, J Johnstone, G Bourke, A Brunelli, H Elkadi, M Otify, C Pompili, J R Burke, E Bagouri, M Chowdhury, Z Abual-Rub, A Kaufmann, S Munot, T Lo, A Young, M Kowal, J Wall, A Peckham-Cooper, G R Layton, B Karki, H Jeong, S Pankhania, S Asher, A Folorunso, S Mistry, B Singh, J Winyard, J Mangwani, E J Caruana, A Mohammad, M Acharya, K Chandarana, K Ang, M F Chowdhry, S Rathinam, A Nakas, A Boddy, T Hossain, C Ashmore, S Annamalai, A Kourdouli, E irvine, A Al-Harbawi, K Kassam, A Al-Harbawee, A Miller, M Mair, R Lunevicius, A R G Sheel, M Sundhu, A J A Santini, M S A T Fathelbab, K M A Hussein, Q M Nunes, R P Jones, K Shahzad, i Haq, M M A S Baig, J L Hughes, A Kattakayam, K Rajput, N Misra, S B Shah, A L Clynch, N Georgopoulou, H M Sharples, A A Apampa, i C Nzenwa, A Sud, A Harky, B H Kirmani, M Shackcloth, M D Jenkinson, R Zakaria, T Elmoslemany, C P Millward, R Baron, D Dunne, P Szatmary, A Thomas, F McNicol, S Gahunia, D Sochorova, G E Nita, R McKinney, J Russ, J R Tan, R Harwood, H J Corbett, C Rossborough, B L Skelly, N A Che Bakri, S Nazarian, R Vashisht, L Jiao, Z Jawad, A Y Allan, C Kontovounisios, T Grove, O Warren, M G Fadel, M Chatzikonstantinou, P Sorelli, S Rahman, M Hadjipavlou, C Holbrook, C Chong, D Kufeji, S R Rufai, I C Lloyd, G James, A Chari, A H D Silva, L Stroman, B Challacombe, A Sayasneh, M Najdy, A Bill√®, S Fraser, P Agoston, V Rizzo, J King, R Nath, S McCrindle, G Mehra, K Harrison-Phipps, J Pilling, L Okiror, T Routledge, L Mills, A Wali, K El-Boghdadly, C Fotopoulou, S Saso, M Fehervari, J Ploski, S Ghaem-Maghami, D Spalding, P Rajagopal, M Pai, N Habib, Z Jawad, S Hamrang-Yousefi, L Jiao, S Tayeh, T Chase, L Humphreys, J Ayorinde, A Ghanbari, T Cuming, N Anscomb, R Baldwin-Smith, M Rizk, C Grainger, M Davies, A Surendran, J W Nunoo-Mensah, M Dunstan, P Beak, i Gerogiannis, A Jain, A Menon, B Pramodana, D Choi, H J Marcus, L Webber, R May, R Hutchison, V Luoma, S Ranjit, J Parakh, V Sarodaya, A Daadipour, M Khalifa, K D Bosch, V Bashkirova, L S Dvorkin, V K Kalidindi, J Dudek, T Singhal, S El-Hasani, A De Souza, M Cannoletta, M Rochon, S Bhudia, S Bennett, L Navaratne, M Venn, V Yip, B Kayani, C Sohrabi, H M Kocher, A Minicozzi, A Banerjee, T Sullivan, R Sivaprakasam, A Anzak, K Ghufoor, M A Thaha, C Knowles, F S Ledesma, P Patki, D Popova, P Sadigh, R Ramamoorthy, C Uff, L Attwell, C Tanabalan, M A Goh, J D Jayasinghe, I Leal Silva, B Thakur, M Lebe, M S Thet, F Hughes, R Rahman, O Fuwa, J Sanders, A Oo, T Bueser, M Curtis, S A Stamenkovic, T Abbott, S Anwar, C Sohrabi, K Williams, E Chung, R Hagger, A Karim, A Hainsworth, M Flatman, A Trompeter, C Hing, O Brown, P Tsinaslanidis, M W Benjamin, A Leyte, C Tan, J Smelt, P Vaughan, G Santhirakumaran, i Hunt, M Raza, A Labib, X Luo, A Sudarsanam, A Rolls, O Lyons, S Onida, J Shalhoub, K Sugand, C Park, K M Sarraf, S Erridge, J Kinross, M Denning, S Yalamanchili, A Abuown, M ibrahim, G Martin, D Davenport, S Wheatstone, V Kasivisvanathan, K Kapriniotis, A Elhamshary, S M B imam, N Kalavrezos, D Sinha, M Chand, L Green, N Beech, R McEwen, H Kiconco, S M Andreani, M F Bath, A Sahni, N Judkins, L Rigueros Springford, C Sohrabi, J Bacarese-Hamilton, F G Taylor, P Patki, C Tanabalan, C Parmar, S McCluney, S Shah, R Talwar, K Patel, A Askari, P S Jambulingam, S Shaw, A Maity, C Hatzantonis, J Sagar, S Kudchadkar, N Cirocchi, C H Chan, J Reynolds, M E Alexander, C J Smart, B Jayasankar, D Balasubramaniam, K Abdelsaid, N Mundkur, B Gallagher, J Shah, J Anthoney, O Emmerson, N Stylianides, M Abdalla, K Newton, K Bhatia, R Edmondson, L Abdeh, D Jones, M Zeiton, O ismail, H Naseem, R Advani, S Duff, F Moura, B C Brown, A Khan, P Asaad, B Wadham, i A Aneke, J Collis, H Warburton, A Fell, A Smith, C Halkias, J Evans, S Nikolaou, C English, S Kristinsson, T Oni, N ilahi, K Ballantyne, Z Woodward, R Merh, J Dunning, Y Viswanath, K Freystaetter, J Dixon, J N Hadfield, A Hilley, A Egglestone, B Smith, T Hine, B Keeler, R E Soulsby, A Taylor, E Davies, O Ryska, T Raymond, S Rogers, A Tong, P Hawkin, S Tingle, F Abbadessa, A Sachdeva, B Rai, C D Chan, i McPherson, K Booth, F Mahmoud Ali, S Pandanaboyana, T Grainger, S Nandhra, A Patience, A Rogers, C Roy, T Williams, N Dawe, C McCaffer, J Riches, S Bhattacharya, J Moir, N S Kalson, H Elamin Ahmed, C Mellor, C Saleh, R M Koshy, J Hammond, L Sanderson, S Wahed, A W Phillips, K Ghosh, A Tang, A J Beamish, C Price, D Bosanquet, D Magowan, F Solari, G Williams, H Nassa, L Smith, B Robertson-Smith, A Mahmoud, P Ameerally, J G Finch, C Gnanachandran, i Pop, M Rogers, Y Yousef, i Mohamed, R Woods, H Zahid, G Mundy, M Youssef, L Sreedharan, D Baskaran, i Shaikh, K Seebah, J Reid, D Watts, V Kouritas, D Chrastek, G Maryan, D F Gill, F Khatun, K Gajjar, K Williamson, D Bratt, K Konstantinidi, T Walton, N Burnside, H Weaver, M Hawari, E Addae-Boateng, R A Rollett, M L Collins, M S Tamimy, H Riyat, J Wen, J Neil-Dwyer, H Brewer, D Humes, D Worku, A Chowdhury, O Oyende, C Lewis-Lloyd, A Adiamah, A Koh, J Jackman, R Vohra, A Navarro, J Reilly, A Aujayeb, D Townshend, N McLarty, A Shenfine, K Jackson, C Johnson, D Dass, D Ford, S C Winter, E Belcher, D Stavroulias, F Di Chiara, K Wallwork, A Qureishi, M Lami, S Sravanam, S Mastoridis, K Shah, S Chidambaram, R Smillie, A V Shaw, S Bandyopadhyay, C Cernei, C Bretherton, D Jeyaretna, M Ganau, R J Piper, E Duck, S Brown, C Jelley, S C Tucker, G Bond-Smith, X L Griffin, G D Tebala, N Neal, M Vatish, T M Noton, H Ghattaura, M Maher, H Fu, O B F Risk, H Soleymani Majd, S Sinha, S Shankar, A Aggarwal, H Kharkar, K Lakhoo, C Verberne, S Mastoridis, B Dean, C Luney, R Myatt, M A Williams, J McVeigh, L J Rogers, P L Labib, D Miller, G Minto, N Hope, A Marchbank, K Emslie, P Panahi, B Ho, C Perkins, E Clough, H Roy, I Enemosah, R Campbell, J Natale, K Gohil, M Rela, N Raza, I Biliatis, J Khan, G Thiruchandran, S K C Toh, Y Ahmad, A Allana, C Bellis, O Babawale, Y C Phan, U Lokman, M Ismail, T Koc, A Witek, L Duggleby, S Shamoon, S Stefan, H Clancy, R Chadha, S B Middleton, K Wilmott, C Hayden, C Mclaren, J Sutton, A Whyte, A Belgaumkar, A Day, C Gilbert, B Oyewole, P Narayan, H Dent, A Sandhya, T De Silva, S Waheed, A Day, K Kapoor, A P Belgaumkar, P Narayan, M Fahim, T Gala, R Mithany, R Morgan, M Abdelkarim, S Ibrahim, A Maw, A Asqalan, G Sundaram Venkatesan, S Singh, S Mukherjee, D Ferguson, C Smith, A Mansuri, A Thakrar, L Wickramarachchi, R Cuthbert, S Sivayoganathan, K Chui, E Karam, C Dott, S Shankar, K Madhvani, M Hampton, A P Hormis, M Thomas, L Pearce, D M Fountain, R Laurente, K V Sigamoney, M Dasa, K George, Z Naqui, M Galhoum, C Lipede, A Gabr, A Radhakrishnan, M T Hasan, R Kalenderov, O Pathmanaban, F Colombo, R Chelva, G Branagan, L Longstaff, D Ding, C Barlow, J Foster, J Edwards, A Ward, D Tadross, L Majkowski, C Blundell, S Forlani, R Nair, S Guha, S R Brown, C Steele, C J Kelty, T Newman, M Lee, G Chetty, G Lye, S P Balasubramanian, N Sureshkumar Shah, M Sherif, A Al-mukhtar, E Whitehall, A Giblin, F Wells, A Sharkey, A Adamec, S Madan, B Narice, M Sterrenburg, A Thompson, I Varley, M Stavrakas, O Rominiyi, J Ray, A Adamec, M Crank, A Bacon, Y Al-Tamimi, J Catto, S Saad, N N Abd Kahar, S Sinha, A Sou, D Simpson, E Hamilton, J Blair, S Jallad, J Lord, C Anderson, J El Kafsi, K Logishetty, A Saadya, R Midha, M Ip, H Subbiah Ponniah, T Stockdale, T Bacarese-Hamilton, L Foster, A James, N Anjarwalla, D Marujo Henriques, R Hettige, C Baban, A Tenovici, G Salerno, R Singh, J Lane, H V Colvin, A Badran, A Cadersa, S Williams, A Cumpstey, Z Hamady, R Aftab, F Wensley, J Byrne, V Morrison-Jones, G K Sekhon, H Shields, Z Shakoor, A Yener, T Talbot, A Khan, A Alzetani, R Cresner, B H B Babu, A S D Liyanage, S Newman, I Blake, C Weerasinghe, R Baumber, J Parry, C Menakaya, J I Webb, M Antar, N Modi, R Sofat, J Noel, R Nunn, S Adegbola, F Eriberto, V Sharma, R Tanna, S Lodhia, D Johnson, I Hughes, J Hall, J Rooney, S Chatterji, Y Zhang, R Owen, M Rudic, J Hunt, D Zakai, M Thomas, A Aladeojebi, M Ali, A Gaunt, B Barmayehvar, M Kitchen, M Gowda, F Mansour, M Jarvis, E Halliday, R Lefroy, P Nanjaiah, S Ali, M Kitchen, D J Lin, A D Rajgor, R J Scurrah, C Kang, L J Watson, G Harris, T Royle, Y Cunningham, G James, B Steel, A C O Luk, A J Boulton, M T Khan, G Bakolas, I Ahmed, P Herrod, E Gemmill, H Boyd-Carson, M Jibreel, E Lenzi, T Saafan, D Sapre, Z Li, K Parkins, N Spencer, R Harries, R J Egan, D Motter, C Jenvey, R Mahoney, N Fine, T Minto, A Henry, M Hollyman, C Grieco, C Gemmell, H Whitmore, M S Babar, S Goodrum, R Scott, B Collard, K Lau, E Thomas, A Patel, J Allison, J Bowen, A Dias, B Mahendran, S Gopalswamy, S Patil, L Scott, J Sarveswaran, M Michel, S Ravindran, K Subba, A K Abou-Foul, M Khalefa, F Hossain, T Moores, L Pickering, G Stables, A Doorgakant, V G Thiruvasagam, J Carter, S Reid, R Mohammed, W Marlow, H Ferguson, R Wilkin, C Konstantinou, D Yershov, J Vatish, A Denning, H B Shah, G W V Cross, P Seyed-Safi, Y W Smart, A Kuc, M Al-Yaseen, J Olivier, M Hanna, P Eskander, R Duncan, S Halaseh, R Das, H Wynn Jones, H Divecha, C Whelton, T Board, S Powell, C Magee, K Agarwal, E Mangos, T Nambirajan, R Vidya, G Chauhan, J Kaur, A Burahee, S Bleibleh, N Pigadas, D Snee, S Bhasin, A Crichton, A Habeebullah, A S Bodla, N Yassin, M Mondragon, V Dewan, I Flindall, V Mahendran, A Hanson, E Jenner, J Richards, K Thomas-Fernandez, R Wall, A Alqallaf, A Ben-Sassi, I Mohamed, K Mellor, P Joshi, Y Joshi, R Young, V Miu, K Sheridan, L MacDonald, S Green, L Onos, J J Wong, L Napolitano, M Hemmila, D Amin, S Abramowicz, S M Roser, K A Olson, C Riley, C Heron, T Cardenas, E Leede, M Thornhill, A B Haynes, K McElhinney, S Roward, M D Trust, C E Hill, P G Teixeira, E Etchill, K Stevens, M R Ladd, C Long, J Rose, A Kent, P Yesantharao, D Vervoort, H Jenny, A Gabre-Kidan, A Margalit, L Tsai, H Malapati, L Yesantharao, H Abdou, J Diaz, M Richmond, J Clark, L O'Meara, N Hanna, Y Ying, J Fleming, A Ovaitt, J Gigliotti, A Fuson, Z Cooper, A Salim, S A Hirji, A Brown, C Chung, L Hansen, B U Okafor, V Roxo, C P Raut, J S Jolissaint, D A Mahvi, H Kaafarani, K Breen, B Bankhead-Kendall, O Alser, H Mashbari, G Velmahos, L R Maurer, M El Moheb, A Gaitanidis, L Naar, M A Christensen, C Kapoen, K Langeveld, M El Hechi, A Mokhtari, M H Haqqani, F T Drake, A Goldenberg-Sandau, B Galbreath, C Reinke, S Ross, K Thompson, D Manning, R Perkins, E Eriksson, H Evans, M Masrur, P Giulianotti, E Benedetti, G Chang, J Ourieff, D Dehart, A Dorafshar, T Price, A R Bhama, A Torquati, E Cherullo, R Kennedy, J Myers, K Rubin, V S Ban, S G Aoun, H H Batjer, J Caruso, H Carmichael, C G Velopulos, F L Wright, S Urban, R C McIntyre, T J Schroeppel, E A Hennessy, J Dunn, L Zier, C Burlew, J Coleman, K P Colling, B Hall, H E Rice, E S Hwang, S A Olson, D Moris, R Verma, R Hassan, A Volpe, S Merola, L A O'Banion, J Lilienstein, R Dirks, H Marwan, M Almasri, G Kulkarni, M Mehdi, A Abouassi, M Abdallah, M San Andrés, J Eid, E Aigbivbalu, J Sundaresan, B George, A Ssentongo, P Ssentongo, J S Oh, J Hazelton, J Maines, N Gusani, M Garner, S Horvath, F Zheng, M Ujiki, G Kinnaman, A Meagher, I Sharma, E Holler, K McKenzie, J Chan, K Fretwell, W Nugent III, A Khalil, D Chen, N Post, T Rostkowski, D Brahmbhatt, K Huynh, M L Hibbard, M Schellenberg, R C G Martin, N Bhutiani, E Giorgakis, J Laryea, A Bhavaraju, K Sexton, M Roberts, M Kost, M Kimbrough, L Burdine, K Kalkwarf, R Robertson, A Gosain, L Camp, R Lewit, J P Kronenfeld, E Urrechaga, N Goel, R Rattan, V Hart, M Allen, G Gilna, A Cioci, G Ruiz, M Allen, K Rakoczy, W Pavlis, R Saberi, R Morris, B S Karam, C E M Brathwaite, H Liu, P Petrone, H Hakmi, A H Sohail, G Baltazar, R Heckburn, R M Nygaard, E T Colonna, F W Endorf, M J Hill, A Maiga, B Dennis, J H Levin, M Lallemand, R Choron, G Peck, F Soliman, S Rehman, N Glass, B Juthani, D Deisher, N M Ruzgar, S J Ullrich, M Sion, C Paranjape, M El Moheb, A R Kar, C Gillezeau, J Rapp, E Taioli, B A Miles, N Alpert, D Podolsky, N L Coleman, M P Callahan, I Ganly, L Brown, J R T Monson, A Dehal, A Abbas, A Soliman, B Kim, C Jones, E Dauer, E Renza-Stingone, E Hernandez, E Gokcen, E Kropf, H Sufrin, H Hirsch, H Ross, J Engel, J Sewards, J Diaz, J Poggio, K Sanserino, L Rae, M Philp, M Metro, P McNelis, R Petrov, S Rehman, T Pazionis, B Till, R Lamm, A J Rios-Diaz, F Palazzo, M Rosengart, K Nicholson, M M Carrick, K Rodkey, A Suri, R Callcut, S Nicholson, N Talathoti, D Klaristenfeld, W* Biffl, C Marsh, K Schaffer, A E Berndtson, S Averbachs, T Curry, R Kwan-Feinberg, E Consorti, R Gonzalez, R Grolman, T Liu, O Merzlikin, M K Abel, D Ozgediz, M Boeck, L Z Kornblith, B Nunez-Garcia, B Robinson, P Park, A F Utria, S E Rice-Townsend, P Javid, J Hauptman, K Kieran, D Nehra, A Walters, J Cuschieri, G H Davidson, J Nunez, R Cosker, S Eckhouse, A Choudhry, W Marx, T Jamil, S Seegert, S Al-Embideen, M Quintana, H Jackson, S D Wexner, I Kent, P N Martins, M Alshehari, H Al-Naggar, M Alsayadi, M Alyazidi, S Shream, W Alhaddad, A Maqus, M Abu Hamra'a, R Alsayadi, R Ghannam, S Al-Maqtari, S Masdoos, Y Al-Harazi, H Bajjah, S Al-Ameri, M Aldawbali, G P Gwini, D Mazingi

## Abstract

To support the global restart of elective surgery, data from an international prospective cohort study of 8492 patients (69 countries) was analysed using artificial intelligence (machine learning techniques) to develop a predictive score for mortality in surgical patients with SARS-CoV-2. We found that patient rather than operation factors were the best predictors and used these to create the COVIDsurg Mortality Score (https://covidsurgrisk.app). Our data demonstrates that it is safe to restart a wide range of surgical services for selected patients.

## Introduction

Since the beginning of the COVID-19 pandemic tens of millions of operations have been cancelled^1^ as a result of excessive postoperative pulmonary complications (51.2 per cent) and mortality rates (23.8 per cent) in patients with perioperative SARS-CoV-2 infection^2^. There is an urgent need to restart surgery safely in order to minimize the impact of untreated non-communicable disease.

As rates of SARS-CoV-2 infection in elective surgery patients range from 1–9 per cent^3–8^, vaccination is expected to take years to implement globally^9^ and preoperative screening is likely to lead to increasing numbers of SARS-CoV-2-positive patients, perioperative SARS-CoV-2 infection will remain a challenge for the foreseeable future.

To inform consent and shared decision-making, a robust, globally applicable score is needed to predict individualized mortality risk for patients with perioperative SARS-CoV-2 infection. The authors aimed to develop and validate a machine learning-based risk score to predict postoperative mortality risk in patients with perioperative SARS-CoV-2 infection.

## Methods

### Cohort study design

An international, multicentre, prospective, cohort study (COVIDSurg Cohort Study) included consecutive patients who were diagnosed with SARS-CoV-2 in the 7 days before or the 30 days after surgery. Patients undergoing any type of surgery were included from 1 February 2020 to 31 July 2020. Full study methodology has been published previously^2^.

Machine learning methods were used to analyse the COVIDSurg Cohort Study dataset. Sixteen patient and operative variables were entered into the analysis and, based on time, data were split into a derivation set, where all analysis and modelling were performed, and a validation set, where the final model was assessed. Five features were selected following variable importance measurements from both linear and non-linear modelling. These features were then combined into 26 different predictor sets, which were fitted through three different algorithms (logistic regression, decision trees and random forest), generating a total of 78 different models. These models were tuned with a 10-fold cross-validation, fitted in the 75 per cent split of the derivation set and assessed in the remaining 25 per cent. In order to ascertain the model’s stability, this training and testing split was randomly repeated 100 times (bootstraps). Finally, to decide which model to select, performance was evaluated through the mean area under the receiver operating characteristic curve (AUROC) value. A full description of methods is available in *Appendix S1*.

## Results

### Patients

**Fig. 1:**
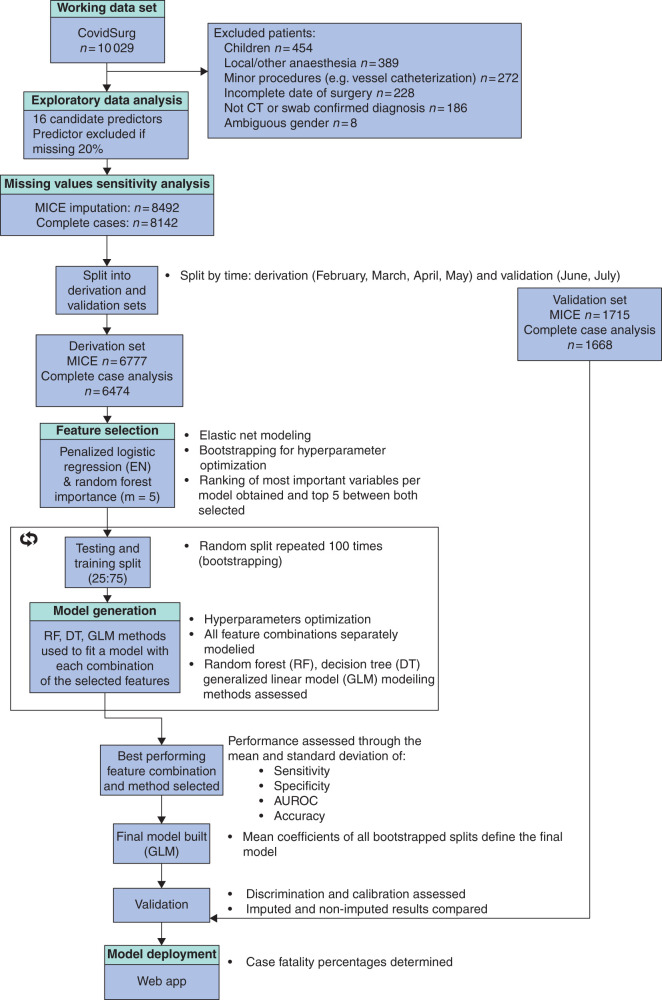
Cohort study patient inclusion and model derivation and validation flow MICE, multiple imputation by chained equations; EN, elastic net.

From 10 029 patients in the cohort study, data from 8492 patients entered the machine learning processes (*Fig. 1*). The most common surgical procedures were abdominal (40.6 per cent, 3446 of 8492 patients), orthopaedic (33.8 per cent, 2867 of 8492) and head and neck surgery (9.8 per cent, 835 of 8492). Emergency surgery accounted for 80.8 per cent (6862 of 8492 patients) and benign disease for 57.3 per cent (4868 of 8492) (*Table S1*).

Of the 8492 patients, data from 6777 patients who had surgery from February to May 2020 (726 hospitals, 65 countries) were entered into the derivation set and data from 1715 who had surgery from June to July 2020 (296 hospitals, 53 countries) were entered into the validation set. Despite changes in patient demographics and treatment options, such as the recommendations from the RECOVERY trial^10^ in June 2020, model performance remained robust between the derivation and validation sets. Mortality rates were 17.2 per cent overall (1462 of 8492 patients), 18.9 per cent (1279 of 6777) in the derivation set and 10.7 per cent (183 of 1715) in the validation set. Missing data handling is described in *Appendix S2*.

### Feature selection

**Fig. 2:**
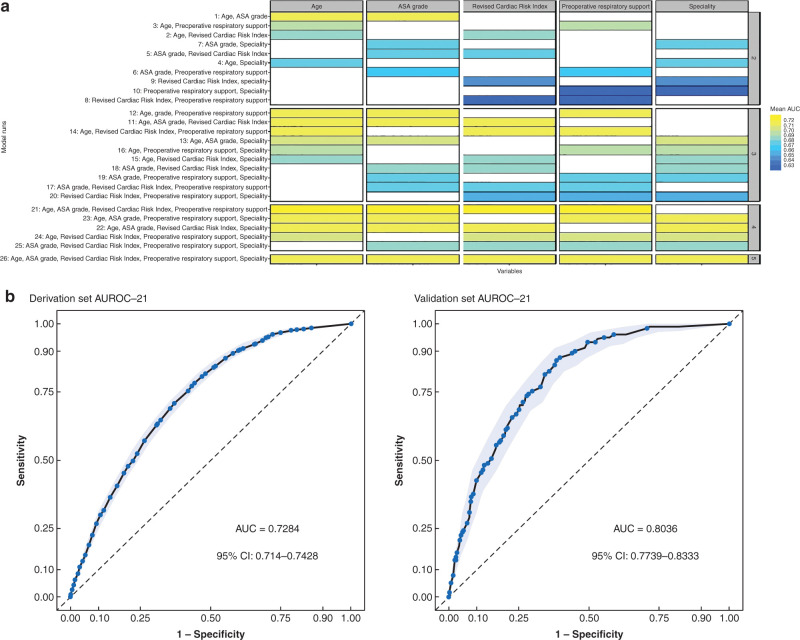
Model performance and evaluation. **a** Logistic model performance of all different runs divided by each of the features. Colour coded according to performance values (mean area under the curve (AUC) of 100 bootstraps). **b** Receiver operating characteristic curves for model evaluation. After generating the final model made up by the averaged coefficients of the bootstraps of run 21, it was evaluated in both the derivation set as a whole (Area under receiver operating characteristic curve (AUROC) = 0.7284, 95 per cent c.i. 0.7140 to 0.7428) and the validation set AUROC = 0.8036, 95 per cent c.i. 0.7739 to 0.8333). Results are depicted with AUROC and confidence intervals generated through the pROC and plotROC packages. 95 per cent confidence intervals were computed with default 2000 stratified bootstrap replicates.

A full breakdown of feature and model selection are in *Tables S3–S7*. Random forest and elastic net were applied to the derivation set and the most important features were ranked (*Fig. S1*). Logit model performance of all different feature combination (runs) divided by each of the features is shown in *Fig. 2a*. The five features taken forward into model building were age, preoperative respiratory support, ASA grade, specialty and revised cardiac risk index (RCRI) score.

### Model building and validation

Multivariable models were built using all possible combinations of the five selected features, resulting in 26 model runs (*Table S8*). The smallest, higher-performing and most robust model was a logistic regression model with four features (age, ASA grade, RCRI score and preoperative respiratory support). A full description is in *Appendix S2*.

The AUROC for the final score was 0.73 (95 per cent c.i. 0.71 to 0.74) in the derivation set and 0.80 (95 per cent c.i. 0.77 to 0.83) in the validation set (*Fig. 2b*). Calibration in the validation set is shown in *Fig. S2* (Brier score 0.084, intercept 0.078, slope 1.08).

Mortality rates were studied for different probability risk cut-offs (*Fig. S3*), with a balance between distribution and clinical importance leading to cut-off values at approximately 2, 10, 30 and 45 per cent mortality. These showed high concordance between derivation and validation sets (*Table S7*) with the final mortality percentages in the combined dataset being very similar.

### Deployment

The COVIDSurg Mortality Score has been deployed online at http://covidsurgrisk.app/.

## Discussion

Understanding risks related to SARS-CoV-2 and COVID-19 will remain important for the foreseeable future. There is an urgent need to restart elective surgery in order to reduce the backlog of cancelled operations^11^. Vaccination programmes are expected to take years to deliver, particularly in low- and middle-income countries^9^, and may not protect against new variants^12^. COVID-19-free surgical pathways are unlikely to be universally implemented, particularly in emergency settings^3^, leaving patients at risk of in-hospital SARS-CoV-2 transmission. Research is critical to mitigate impact. The COVIDsurg Morality Score is a tool that can help overcome these challenges and has clear clinical applications. It can inform consent and shared decision making by patients and surgeons. If individual patient mortality risk is too high, they may choose to postpone surgery. If surgery is unavoidable, risks should be clearly documented as part of informed consent, and postoperative critical care can be identified and justified. It can also identify patients who are at very low risk, where surgery can continue as planned.

The COVIDSurg Mortality Score shows high discrimination for predicting mortality in patients with perioperative SARS-CoV-2. The AUROCs in the derivation (0.73) and validation (0.80) sets are consistent with published non-surgical COVID risk scores^13^. The COVIDSurg Mortality Score is globally applicable and consists of variables that are readily available across all resource settings. The score was derived and validated in a global data set (all types of surgery, 756 hospitals, 69 countries) and demonstrated validity across geographic regions and over time as community SARS-CoV-2 infection rates fluctuated.

**Table:** **PROBAST: Prediction model**  **Risk Of Bias ASsessment Tool** **Step 1: Specify your**  **systematic review question(s) – not applicable.** **Step 2: Classify the type of prediction**  **model evaluation –** development and validation. **Step 3: Assess risk of**  **bias and applicability**

Risk of Bias			
Participants	Predictors	Outcome	Analysis
Were appropriate data sources used, e.g., cohort, RCT, or nested case–control study data? **Yes**	Were predictors defined and assessed in a similar way for all participants? **Yes**	Was the outcome determined appropriately? **Yes**	Were there a reasonable number of participants with the outcome? **Yes**
Were all inclusions and exclusions of participants appropriate? **Yes**	Were predictor assessments made without knowledge of outcome data? **Yes**	Was a prespecified or standard outcome definition used? **Yes**	Were continuous and categorical predictors handled appropriately? **Yes**
	Are all predictors available at the time the model is intended to be used? **Yes**	Were predictors excluded from the outcome definition? **Yes**	Were all enrolled participants included in the analysis? **No**
		Was the outcome defined and determined in a similar way for all participants? **Yes**	Were participants with missing data handled appropriately? **Yes**
		Was the outcome determined without knowledge of predictor information? **Yes**	Was selection of predictors based on univariable analysis avoided? **No**
		Was the time interval between predictor assessment and outcome determination appropriate? **Yes**	Were complexities in the data (e.g., censoring, competing risks, sampling of control participants) accounted for appropriately? **Yes**
			Were relevant model performance measures evaluated appropriately? **Yes**
			Were model overfitting, underfitting, and optimism in model performance accounted for? **Yes**
			Do predictors and their assigned weights in the final model correspond to the results from the reported multivariable analysis? **Yes**

ROB = risk of bias. + indicates low ROB/low concern regarding applicability; − indicates high ROB/high concern regarding applicability; and ? indicates unclear ROB/unclear concern regarding applicability.

**Table:** Step 4: Overall judgment

ROB				Applicability			Overall	
Participants	Predictors	Outcome	Analysis	Participants	Predictors	Outcome	ROB	Applicability
+	+	+	+	NA	NA	NA	+	NA

The COVIDSurg cohort study was prospectively collected and conducted, but it had limitations. Variables were coded following standardized medical criteria but a further analysis of continuous variables as well as the inclusion of interactions^14^ could be incorporated in future studies. As there are no other international, multispecialty data sets for SARS-CoV-2 surgical patients, further external validation was not possible, but should be considered if such data sets emerge. High mortality percentages (approximately 47 per cent) at higher probability outputs (greater than 0.5) should be interpreted with caution, as numbers of patients with these feature combinations were low. The authors acknowledge that the model will warrant updates as more data become available.

Despite differences in mortality between the derivation and validation sets, model performance remained robust. There are likely to have been structural and system differences between hospitals that were not captured in the cohort study. These differences are further compounded by variation in community SARS-CoV-2 across time and across geographical locations. Nonetheless, the score performed well in the validation set, suggesting the score can be used reliably both during COVID waves and troughs.

## Collaborators

**Writing group:** Laura Bravo* (UK), Dmitri Nepogodiev* (UK), James C Glasbey* (UK), Elizabeth Li* (UK), Joana FF Simoes* (Portugal), Sivesh K Kamarajah (UK), Maria Picciochi (Portugal), Tom EF Abbott (UK), Adesoji O Ademuyiwa (Nigeria), Alexis P Arnaud (France), Arnav Agarwal (Canada), Amanpreet Brar (Canada), Muhammed Elhadi (Libya), Dennis Mazingi (Zimbabwe), Victor Roth Cardoso (UK), Samuel Lawday (UK), Raza Sayyed (Pakistan), Omar M Omar (UK), Antonio Ramos de la Madina (Mexico), Luke Slater (UK), Mary Venn (UK), Georgios Gkoutos (UK)†, Aneel Bhangu (UK)†

*Joint first authors

†Joint Senior Authors

**Statistical analysis:** Laura Bravo, Victor Roth Cardoso, Luke Slater, Andreas Karwath, Georgios Gkoutos. Underlying data have been verified by Dmitri Nepogodiev, Laura Bravo, Aneel Bhangu (overall study guarantor).

**Operations Committee:** Kwabena Siaw-Acheampong, Leah Argus, Daoud Chaudhry, Brett E. Dawson, James C. Glasbey, Rohan R. Gujjuri, Conor S. Jones, Sivesh K. Kamarajah, Chetan Khatri, James M. Keatley, Samuel Lawday, Elizabeth Li, Harvinder Mann, Ella J Marson, Kenneth A. Mclean, Maria Picciochi, Elliott H. Taylor, Abhinav Tiwari, Joana F.F. Simoes, Isobel M. Trout, Mary L. Venn, Richard J.W. Wilkin, Aneel Bhangu*, Dmitri Nepogodiev*

*Co-chairs

**Dissemination Committee:** Irida Dajti (Albania), Arben Gjata (Albania), Luis Boccalatte (Argentina), Maria Marta Modolo (Argentina), Daniel Cox (Australia), Peter Pockney (Australia), Philip Townend (Australia), Felix Aigner (Austria), Irmgard Kronberger (Austria), Kamral Hossain (Bangladesh), Gabrielle VanRamshorst (Belgium), Ismail Lawani (Benin), Gustavo Ataide (Brazil), Glauco Baiocchi (Brazil), Igor Buarque (Brazil), Muhammad Gohar (Bulgaria), Mihail Slavchev (Bulgaria), Arnav Agarwal (Canada), Amanpreet Brar (Canada), Janet Martin (Canada), Maria Marta Modolo (Chile), Maricarmen Olivos (Chile), Jose Calvache (Colombia), Carlos Jose Perez Rivera (Colombia), Ana Danic Hadzibegovic (Croatia), Tomislav Kopjar (Croatia), Jakov Mihanovic (Croatia), Jaroslav Klat (Czech Republic), René Novysedlak (Czech Republic), Peter Christensen (Denmark), Alaa El-Hussuna (Denmark), Sylvia Batista (Dominican Republic), Eddy Lincango (Ecuador), Sameh H Emile (Egypt), Mengistu Gebreyohanes Mengesha (Ethiopia), Samuel Hailu (Ethiopia), Hailu Tamiru (Ethiopia), Joonas Kauppila (Finland), Johanna Laukkarinen (Finland), Alexis Arnaud (France), Markus Albertsmeiers (Germany), Hans Lederhuber (Germany), Markus Loffler (Germany), Stephen Tabiri (Ghana), Symeon Metallidis (Greece), Georgios Tsoulfas (Greece), Maria Aguilera Lorena (Guatemala), Gustavo Grecinos (Guatemala), Tamas Mersich (Hungary), Daniel Wettstein (Hungary), Dhruv Ghosh (India), Gabriele Kembuan (Indonesia), Peiman Brouk (Iran), Mohammad Khosravi (Iran), Masoud Mozafari (Iran), Ahmed Adil (Iraq), Helen M. Mohan (Ireland), Oded Zmora (Israel), Marco Fiore (Italy), Gaetano Gallo (Italy), Francesco Pata (Italy), Gianluca Pellino (Italy), Sohei Satoi (Japan), Faris Ayasra (Jordan), Mohammad Chaar (Jordan), Ildar R Fakhradiyev (Kazakhstan), Mohammad Jamal (Kuwait), Muhammed Elhadi (Libya), Aiste Gulla (Lithuania), April Roslani (Malaysia), Laura Martinez (Mexico), Antonio Ramos De La Medina (Mexico), Oumaima Outani (Morocco), Pascal Jonker (Netherlands), Schelto Kruijff (Netherlands), Milou Noltes (Netherlands), Pieter Steinkamp (Netherlands), Willemijn van der Plas (Netherlands), Adesoji Ademuyiwa (Nigeria), Babatunde Osinaike (Nigeria), Justina Seyi-olajide (Nigeria), Emmanuel Williams (Nigeria), Sofija Pejkova (North Macedonia), Knut Magne Augestad (Norway), Kjetil Soreide (Norway), Zainab Al Balushi (Oman), Ahmad Qureshi (Pakistan), Raza Sayyed (Pakistan), Mustafa Abu Mohsen Daraghmeh (Palestine), Sadi Abukhalaf (Palestine), Moises Cukier (Panama), Hugo Gomez (Paraguay), Sebastian Shu (Peru), Ximena Vasquez (Peru), Marie Dione Parreno-Sacdalan (Philippines), Piotr Major (Poland), José Azevedo (Portugal), Miguel Cunha (Portugal), Irene Santos (Portugal), Ahmad Zarour (Qatar), Eduard-Alexandru Bonci (Romania), Ionut Negoi (Romania), Sergey Efetov (Russia), Andrey Litvin (Russia), Faustin Ntirenganya (Rwanda), Ehab AlAmeer (Saudi Arabia), Dejan Radenkovic (Serbia), Frederick Koh Hong Xiang (Singapore), Chew Min Hoe (Singapore), James Ngu Chi Yong (Singapore), Rachel Moore (South Africa), Ncamsile Nhlabathi (South Africa), Ruth Blanco Colino (Spain), Ana Minaya Bravo (Spain), Ana Minaya-Bravo (Spain), Umesh Jayarajah (Sri Lanka), Dakshitha Wickramasinghe (Sri Lanka), Mohammed Elmujtaba (Sudan), William Jebril (Sweden), Martin Rutegård (Sweden), Malin Sund (Sweden), Arda Isik (Turkey), Sezai Leventoğlu (Turkey), Tom E.F. Abbott (UK), Ruth Benson (UK), Ed Caruna (UK), Sohini Chakrabortee (UK), Andreas Demetriades (UK), Anant Desai (UK), Thomas D. Drake (UK), John G. Edwards (UK), Jonathan P. Evans (UK), Samuel Ford (UK), Christina Fotopoulou (UK), Ewen Griffiths (UK), Peter Hutchinson (UK), Michael D. Jenkinson (UK), Tabassum Khan (UK), Stephen Knight (UK), Angelos Kolias (UK), Elaine Leung (UK), Siobhan McKay (UK), Lisa Norman (UK), Riinu Ots (UK), Vidya Raghavan (UK), Keith Roberts (UK), Andrew Schache (UK), Richard Shaw (UK), Katie Shaw (UK), Neil Smart (UK), Grant Stewart (UK), Sudha Sundar (UK), Dale Vimalchandran (UK), Naomi Wright (UK), Sattar Alshryda (United Arab Emirates), Osaid Alser (USA), Kerry Breen (USA), Ian Ganly (USA), Haytham Kaafarani (USA), Brittany Kendall (USA), Hassan Mashbari (USA), Hamza Al Naggar (Yemen), Dennis Mazingi (Zimbabwe), EuroSurg, European Society of Coloproctology (ESCP), GlobalSurg, GlobalPaedSurg, ItSURG, PTSurg, SpainSurg, Italian Society of Colorectal Surgery (SICCR), Association of Surgeons in Training (ASiT), Irish Surgical Research Collaborative (ISRC), Joana F.F. Simoes (Chair).

Collaborators (*asterix indicates principle investigator):I. Dajti (University Hospital Centre Nene Tereza, Tirana, Albania); J. I. Valenzuela* (Hospital Velez Sarsfield, Buenos Aires, Argentina); L. A. Boccalatte*, N. A. Gemelli, D. E. Smith (Hospital Italiano De Buenos Aires, Buenos Aires, Argentina); N. N. Dudi-Venkata, H. M. Kroon*, T. Sammour (Royal Adelaide Hospital, Adelaide, Australia); M. Roberts*, D. Mitchell*, K. Lah*, A. Pearce, A. Morton (Royal Brisbane and Women's Hospital, Brisbane, Australia); A. C. Dawson*, A. Drane (Gosford Hospital, Gosford, Australia); C. Sharpin*, R. M. Nataraja*, M. Pacilli (Monash Children’s Hospital, Melbourne, Australia); D. R. A. Cox, V. Muralidharan*, G. E. Riddiough, E. M. Clarke, W. Jamel, K. R. Qin (Austin Hospital, Heidelberg, Australia); P. Pockney*, D. Cope, N. Egoroff, N. Lott (John Hunter Hospital, Newcastle, Australia); S. Putnis, S. De Robles, Z. Ang (Wollongong Public Hospital, Wollongong, Australia); M. Mitteregger, S. Uranitsch, M. Stiegler, G. Seitinger, F. Aigner* (Krankenhaus der Barmherzigen Brüder Graz, Graz, Austria); D.B. Lumenta*, S.P. Nischwitz, E. Richtig, M. Pau, P. Srekl-Filzmaier, N. Eibinger, B. Michelitsch, M. Fediuk, A. Papinutti, G. Seidel, J. Kahn, T.U. Cohnert (Medical University of Graz, Graz, Austria); F. Messner, D. Öfner* (Medical University of Innsbruck, Innsbruck, Austria); J. Presl*, M. Varga, M. Weitzendorfer, K. Emmanuel (Paracelsus Medical University, Salzburg, Austria); A. D. Binder (Universitätsklinikum Tulln, Tulln, Austria); M. Zimmermann, S. Holawe, E. Nkenke, C. Grimm, M. Kranawetter (Medical University of Vienna, Vienna, Austria); A. Rahman Mitul*, N. Islam, S. Karim (Dhaka Shishu (Children) Hospital, Dhaka, Bangladesh); N. Komen*, S. Putnis, S. De Robles, E. Ang (University Hospital Antwerp (UZA), Edegem, Belgium); H. De Praetere*, T. Tollens, G. Schols, C. Smets, L. Haenen (Imelda Hospital, Bonheiden, Belgium); J. Quintens, K Van Belle* (Europe Hospitals, Belgium); G.H. Van Ramshorst*, P. Pattyn, L. Desender, T. Martens, D. Van de Putte (Ghent University Hospital, Ghent, Belgium); P Lerut*, A. Grimonprez, M. Janssen, G. De Smul, P. Wallaert (AZ Groeninge, Kortrijk, Belgium); J. Van den Eynde, W. Oosterlinck*, R. Van den Eynde, A. Sermon, A. Boeckxstaens, A. Cordonnier, J. De Coster, J. Jaekers, C. Politis, M. Miserez (UZ Leuven, Leuven, Belgium); N. Duchateau, C. De Gheldere* (Heilig Hart Ziekenhuis Lier, Lier, Belgium); N. Flamey, P. Pattyn* (AZ Delta, Roeselare, Belgium); A. Christiano*, B. Guidi, A. L. Minussi, S. Castro, W. Okoba, F. H. R. Maldonado, P. Oliveira, T. Baldasso, L. Santos (Fundação Centro Médico De Campinas, Campinas, Brazil); G. M. A. Gomes, I. L. Buarque, L. Pol-Fachin, T. S. Bezerra, A. V. Barros*, A. M. R. da Silva, A. L. S. Leite, D. W. A. Silvestre, C. C. Ferro (Hospital Santa Casa De Misericórdia De Maceió, Maceió, Brazil); M. S. Araujo*, L. M. Lopes, P. D. Damasceno, D. H. S. Araujo (Hospital Universitário Antonio Pedro, Niterói, Brazil); G. Laporte*, M. C. Salem (Irmandade Da Santa Casa De Misericórdia De Porto Alegre, Porto Alegre, Brazil); M. A. C. Guimaraes-Filho (Pedro Ernesto University Hospital, Rio de Janeiro, Brazil); L. Nacif* (Hospital Nove De Julho, São Paulo, Brazil); R. L. G. Flumignan*, L. C. U. Nakano, D. A. B. Kuramoto, A. L. S. Aidar, M. R. Pereda, R. M. Correia, B. C. Santos, A. A. Carvalho, J. E. Amorim, H. J. Guedes Neto, L. L. Areias, A. F. Sousa, C. D. Q. Flumignan, W. G. Lustre, D. H. Moreno, N. Barros Jr, J. C. C. Baptista-Silva (Hospital São Paulo, São Paulo, Brazil); L. L. Matos*, L. P. Kowaski, M. A. V. Kulcsar, K. S. Nunes, M. F. Teixeira (Instituto do Câncer do Estado de São Paulo, São Paulo, Brazil); R. L. Nunes*, T. R. Ijichi, N. J. Kim, A. Marreiro, B. Muller, J. Barakat Awada (Notre Dame Intermédica—Hospital Salvalus, São Paulo, Brazil); G. Baiocchi*, L. P. Kowalski*, J. G. Vartanian, F. B. Makdissi, S. Aguiar Jr*, N. Marques, G. B. Carvalho, T. M. D. M. Marques, E. A. Abdallah, C. E. Zurstrassen*, J. L. Gross*, S. C. Zequi*, B. T. Gonçalves, S. S. Santos, J. P. Duprat, F. J. F. Coimbra* (A.C. Camargo Cancer Center, São Paulo, Brazil); R. Cicco* (Instituto de Câncer Dr Arnaldo Vieira de Carvalho, São Paulo, Brazil); F. Takeda, I. Cecconello, U. Ribeiro Jr (Instituto Do Câncer De Estado De São Paulo, São Paulo, Brazil); A. Gatti*, R. Oliva, C. Nardi (Hospital Geral De Pirajussara, São Paulo, Brazil); M. Slavchev*, B. Atanasov, N. Belev (University Hospital Eurohospital, Plovdiv, Bulgaria); A. Dell, D. Bigam*, K. Dajani, S. Al Riyami (University of Alberta Hospital, Edmonton, Canada); J. Martin*, D. Cheng, H. Yang, A. Fayad (London Health Sciences Centre and St Josephs Health Care London, London, Canada); F.M. Carrier*, E. Amzallag, J. Desroches, M. Ruel (Centre hospitalier de l’Université de Montréal, Montréal, Canada); N. G. Caminsky, M. Boutros*, J. Moon, E. G. Wong, T. Vanounou, J. Pelletier, S. Wong, E. Girsowicz, J. Bayne, D. Obrand, H. Gill, O. Steinmetz, K. MacKenzie, M. Lukaszewski, G. Jamjoum (Jewish General Hospital, Montréal, Canada); P. Richebé, O. Verdonck, S. Discepola, N. Godin, M. Idrissi (Hôpital Maisonneuve-Rosemont, Montréal, Canada); D. Briatico, S. Sharma, G. Talwar, K. Bailey (McMaster Children’s Hospital, Hamilton, Ontario, Canada); V Lecluyse* (Hôpital du Sacré-Cœur-de-Montréal, Montréal, Canada); G. Côté (Centre hospitalier universitaire Sainte-Justine, Montréal, Canada); S. Demyttenaere*, R. Garfinkle, M. Boutros* (St. Mary's Hospital, Kitchener, Canada); A. Kouyoumdjian, M. Boutros*, S. Dumitra, J. Moon, E. G. Wong, K. Khwaja, L. Luo, M. Lukaszewski, H. Gill, G. Berry, A. S. Liberman, E. Girsowicz, J. Bayne, D. Obrand, O. Steinmetz, K. MacKenzie, S. Schmid, J. Spicer, M. Al Farsi* (McGill University Health Center, Montréal, Canada); J. Abou-Khalil (The Ottawa Hospital, Ottawa, Canada); E. Couture*, S. Mohammadi, H. Tremblay, N. Gagné, A. Bergeron (Institut universitaire de cardiologie et de pneumologie de Québec, Québec City, Canada); A. F. Turgeon*, O. Costerousse, D. Bellemare, C. Babin, C. Blier (Centre hospitalier universitaire de Québec, Québec City, Canada); M. L. Wood, A. Persad, G. Groot*, H. Pham (Saskatoon City Hospital/Royal University Hospital/St. Paul's Hospital, Saskatoon, Canada); F. D'Aragon*, E. Carbonneau, M. Bouchard, M. Masse, F. Pesant, J. Héroux (Centre hospitalier universitaire de Sherbrooke, Sherbrooke, Canada); P. Karanicolas*, J. Hallet, A. Nadler, A. Nathens (Sunnybrook Hospital, Toronto, Canada); M. Ko*, A. Brar (St. Joseph's Health Centre, Toronto, Canada); K. Mayson* (Vancouver General Hospital, Vancouver, Canada); B. Kidane*, S. Srinathan (Health Sciences Centre, Winnipeg, Canada); M. I. Escudero*, J. T. Reyes (Hospital Clinico Universidad De Chile/Hospital San Jose, Santiago, Chile); M. M. Modolo*, P. Ramirez Nieto, R. Sepulveda, A. Bolbarán, A. Molero, I. Ruiz (Barros Luco Trudeau, Santiago, Chile); G. P. Reyes*, R. Salas, C. Suazo (Hospital del Salvador, Santiago, Chile); R. Muñoz*, E. Grasset*, M. Inzunza, N. Besser, M. J. Irarrázaval, C. Jarry, F. Bellolio, C. A. Romero Manqui, M. Ruiz Esquide (Hospital Clínico Universidad Católica, Santiago, Chile); T. Fuentes*, J. Campos (Hospital Sotero Del Rio, Puente Alto, Chile); C. J. Perez Rivera*, P. A. Cabrera (Fundacion Cardioinfantil-Instituto de Cardiología, Bogotá, Colombia); R. E. Pinilla*, O. Guevara, L. J. Jimenez Ramirez, B. G. Velasquez Cuasquen, D. R. Herrera Mora, A. Bonilla, S. Diaz*, E. Manrique, H. Facundo, J. L. Velez Bernal, J. Ángel, M. García, L. Guzmán, C. Lehmann, S. Cervera, L. M. Trujillo Sanchez (Instituto Nacional De Cancerologia, Bogotá, Colombia); R. Guevara*, D. Valbuena, L. Suarez, G. Jimenez, A. Velandia, J. Vargas, J. Espinosa, S. Rey (Clínica Universitaria Colombia, Bogotá, Colombia); J. Mendoza Quevedo (Subred Sur Occidente de Kennedy (Hospital de Kennedy), Bogotá, Colombia); J. A. Calvache*, C. M. Orozco-Chamorro, T. A. Sánchez-Gómez, D. A. Rojas-Tejada (Hospital Universitario San José, Popayán, Colombia); J. Mihanovic*, B. Bakmaz, I. Rakvin, N. Sulen, T. Andabaka (Zadar General Hospital, Zadar, Croatia); I. Luksic*, M. Mamic (University Hospital Dubrava, Zagreb, Croatia); L. Martinek, M. Skrovina* (Hospital & Oncological Centre Novy Jicin, Novy Jicin, Czech Republic); J. Žatecký*, M. Peteja (Slezská nemocnice v Opavě, p.o., Opavě, Czech Republic); H. Ø. Kristensen, M. Mekhael, P. Christensen*, L. Westh (Aarhus University Hospital, Aarhus, Denmark); H. Smith*, A. F. Haugstvedt, M. L. Jönsson (Bispebjerg Hospital, Copenhagen, Denmark); A. Crespo, S. Batista*, J. Rodriguez-Abreu*, N. Tactuk, P. J. Diaz-Delgado, R. Rivas (Cedimat—Centro De Diagnóstico, Medicina Avanzada, Laboratorio Y Telemedicina, Santo Domingo, Dominican Republic); J. A. Sarmiento-Bobadilla (Hospital General Norte de Guayaquil, Guayaquil, Ecuador); F. Ashoush, A. Samir Abdelaal, M. S. Qatora, M. E. Elsayed Hewalla, M. Metwalli, R. Atta, A. Abdelmajeed, N. E. Abosamak, A. Sabry*, S. Shehata* (Alexandria Main University Hospital, Alexandria, Egypt); I. Sallam*, G. Amira, M. Sherief, A. Sherif (Misr Cancer Centre, Giza, Egypt); H. Salem*, R. Hamdy, H. Aboulkassem, G. Ghaly*, G. Sherif, A. Morsi, A. Abdelrahman (National Cancer Institute, Cairo, Egypt); A. Omnia (Coptic Hospital, Cairo, Egypt); A. Tawheed, M. El Kassas*, W. Omar (Helwan University, Cairo, Egypt); A. Abdelsamed, A. Seleim, H. Salem*, A. Y. Azzam* (Al Azhar University Hospitals, Cairo, Egypt); M. ElFiky*, A. Nabil (Kasr Alainy Faculty of Medicine, Cairo University, Cairo, Egypt); M. Ibraheem*, G. Ghaly, M. ElDeeb, M. Fawzy (Baheya Centre for Early Detection and Treatment of Breast Cancer, Cairo, Egypt); H. Hamed* (Elkheir Hospital, Mansoura, Egypt); S. Emile*, A. Elfallal, H. Elfeki, M. Shalaby, A. Sakr (Mansoura University Hospital, Mansoura, Egypt); M. Alrahawy*, H. Atif, A. Sakr, H. Soltan (Menoufia University Hospital, Menoufia, Egypt); A. K. Sayed, A. Salah, A. Atiya* (Minia University Hospital, Minia, Egypt); K. Wassim* (Quwaysna Central Hospital, Quwaysna, Egypt); A. M. Abbas, H. A. S. Abd Elazeem, A. Y. Abd-Elkariem, M. M. Abd-Elkarem, S. Alaa, A. K. Ali, M. Ashraf, A. Ayman, M. G. Azizeldine, H. Elkhayat, A. Emad Mashhour, M. Gaber, H. M. Hamza, I. Hawal, H. F. Hetta, S. M. Elghazaly, M. M. Mohammed, F. A. Monib, M. A. Nageh, A. Saad, M. M. Saad, M. Shahine, E. A. Yousof, A. Youssef (Assiut University Hospital, Assiut, Egypt); E. Esmail*, M. Khalaf (Kafr El Zayyat Hospital, Kafr El Zayyat, Egypt); A. Eldaly* (El-Menshawy Hospital, El-Menshawy, Egypt); A. Ghoneim*, A. Hawila, H. Badr, I. Elhalaby, M. Abdel-bari, M. Elbahnasawy, M. K. Hamada, M. S. Morsy, M. Hammad, M. Hammad, M. Essa, M. T. Fayed, M. Elzoghby, M. Rady, O. Hamad, S. Salman, S. Sarsik, S. Abd-elsalam, S. Gamal Badr, Y. El-Masry ( University Hospital, Tanta. Egypt); M. M. H. Moahmmed (Zagazig University Hospitals, Zagazig, Egypt); S. Hailu*, A. Wolde, M. Mengesha, S. Nida, M. Workneh, M. Y. Ahmed, T. Fisseha, D. Kassa, H. Zeleke, A. Admasu, T. Laeke, A. Tirsit, M. Gessesse, A. Addissie (Tikur Anbessa (Black Lion) Hospital-Addis Ababa University, Addis Ababa, Ethiopia); D. Bekele* (Maddawalabu University Goba Referral Hospital, Bale Robe, Ethiopia); J. H. Kauppila*, E. Sarjanoja (Länsi-Pohja Central Hospital, Kemi, Finland); S. Testelin*, S. Dakpé, B. Devauchelle, J. Bettoni, N. Lavagen (CHU Amiens, Amiens, France); F. Schmitt*, J. M. Lemée, S. Boucher, R. Breheret, J. D. Kün-Darbois, A. Kahn, A. Gueutier, P. Bigot (CHU Angers, Angers, France); B. Borraccino (Centre Hospitalier Auxerre, Auxerre, France); Z. Lakkis*, A. Doussot, B. Heyd, S. Manfredelli, P. Mathieu, B. Paquette, C. Turco, A. Barrabe, A. Louvrier (CHU Besançon, Besançon, France); D. Moszkowicz*, D. Giovinazzo, F. Bretagnol (Hôpital Louis-Mourier AP-HP, Paris, France); A. Police*, L. Charre, E. Volpin, H. Braham, N. El Arbi, V. Villefranque, L. Bendjemar (Hôpital Simone Veil, Paris, France); E. Girard*, J. Abba, B. Trilling (CHU Grenoble-Alpes, Hôpital Michallon, Grenoble, France); A. Chebaro*, K. Lecolle, S. Truant, M. El Amrani, P. Zerbib, F. R. Pruvot, D. Mathieu, E. Surmei, L. Mattei, H. Marin (CHU Lille, Lille, France); N. Christou, Q. Ballouhey*, P. Ferrero, P. Coste Mazeau, J. Tricard, B. Barrat, A. Taibi, J. Usseglio, J. Laloze, H. Salle, L. Fourcade (CHU Limoges, Limoges, France); E. Duchalais*, N. Regenet, J. Rigaud, D. Waast, W. Denis, O. Malard, K. Buffenoir, F. Espitalier, C. Ferron, Y. Varenne, V. Crenn, S. De Vergie, J. Cristini, E. Samarut (Nantes University Hospital, Nantes, France); S. Tzedakis*, P. A. Bouche*, S. Gaujoux (Hôpital Cochin—AP-HP, Paris, France); E. Kantor (AP-HP Hopital Bichat Claude Bernard, Paris, France); D. Gossot, A. Seguin-Givelet*, D. Fuks, M. Grigoroiu, R. Sanchez Salas, X. Cathelineau, P. Macek, Y. Barbé, F. Rozet, E. Barret, A. Mombet, N. Cathala, E. Brian, F. Zadegan, C. Conso (Institut Mutualiste Montsouris, Paris, France); T. Blanc*, A. Broch, S. Sarnacki (Necker Enfants Malades University Children Hospital–APHP, Paris; Université de Paris, Paris, France); L. Ali, A. Bonnard, M. Peycelon* (Robert-Debré Children University Hospital–APHP, Paris; Université de Paris, Paris, France); E. Hervieux*, P. Clermidi, E. Maisonneuve, E. Aubry, A. Thomin, T. Langlais (Hôpital Trousseau–APHP, Paris, France); G. Passot*, O. Glehen, E. Cotte, J. C. Lifante (Hopital Lyon Sud, Pierre Bénite, France); B. De Simone*, E. Chouillard* (Centre Hospitalier Intercommunal Poissy Saint Germain En Laye, France); A. P. Arnaud*, P. Violas (CHU Rennes–Hôpital Sud, Rennes, France); D. Bergeat, A. Merdrignac, A. P. Arnaud (CHU Rennes–Hopital Pontchaillou, Rennes, France); A. Scalabre*, L. O. Perotto, B. Le Roy, E. Haddad, S. Vermersch (CHU Saint Etienne, Saint Etienne, France); A. C. Ezanno*, O. Barbier, F. Vigouroux, B. Malgras, A. Aime (HIA Begin, Paris, France); B. Seeliger*, D. Mutter, G. Philouze, P. Pessaux (IHU-Strasbourg/Strasbourg University Hospitals, Strasbourg, France); A. Germain*, H. Chanty, A. Ayav (CHRU Nancy, Nancy, France); R. Kassir*, P. Von Theobald, F. Sauvat (CHU Reunion, Reunion, France); J. O'Connor*, M. Mayombo Idiata, Z. O'Connor, S. Tchoba (Bongolo Hospital, Lembamba, Gabon); A. Modabber*, P. Winnand, F. Hölzle (University Hospital Aachen, Aachen, Germany); B. Sommer, E. Shiban, S. Wolf, M. Anthuber*, F. Sommer (University Hospital Augsburg, Augsburg, Germany); D. Kaemmerer*, T. Schreiber (Zentralklinik Bad Berka, Bad Berka, Germany); C. Kamphues*, J. C. Lauscher, C. Schineis, F. N. Loch, K. Beyer (Charité University Medicine, Campus Benjamin Franklin, Berlin, Germany); S. Nasser, J. Sehouli* (Charité Comprehensive Cancer Centre, Berlin, Germany); P. Höhn, C. Braumann*, F. Reinkemeier, W. Uhl (St Josef-Hospital, Bochum, Germany); J. Weitz, U. Bork*, T. Welsch, C. Praetorius, S. Korn, M. Distler (University Hospital Carl Gustav Carus, Technical University Dresden, Dresden, Germany); G. Fluegen, W. T. Knoefel, C. Vay* (University Hospital Düsseldorf, Düsseldorf, Germany); H. Golcher, R. Grützmann*, J. Binder (Universitätsklinikum Erlangen, Erlangen, Germany); P. Meister*, A. Gallinat, A. Paul (University Hospital Essen, Essen, Germany); A. A. Schnitzbauer*, P. Thoenissen, H. El Youzouri, T. Schreckenbach, T. A. Nguyen (Frankfurt University Hospital, Goethe University, Frankfurt, Germany); H. Eberbach*, J. Bayer*, B. Erdle, R. Sandkamp (Medical Centre—Albert-Ludwigs-University of Freiburg, Freiburg, Germany); C. Nitschke, J. Izbicki, F. G. Uzunoglu*, D. Koenig, M. Gosau, A. Böttcher, A. Heuer, T. O. Klatte, M. Priemel, C. S. Betz, S. Burg, N. Möckelmann, C. J. Busch, J. Bewarder, N. Zeller, R. Smeets, S. Thole, T. Vollkommer, U. Speth, M. Stangenberg (University Medical Center Hamburg-Eppendorf, Hamburg, Germany); I. Hakami*, C. Boeker, J. Mall* (KRH Nordstadt-Siloah Hospitals, Hannover, Germany); H. M. Schardey, T. von Ahnen*, M. von Ahnen, U. Brunner (Agatharied Hospital, Hausham, Germany); C. Tapking*, U. Kneser, C. Hirche, M. Jung, K. F. Kowalewski (BG Trauma Centre Ludwigshafen, Ludwigshafen, Germany); P. Kienle* (Theresienkrankenhaus, Mannheim, Germany); C. Reissfelder, S. Seyfried*, F. Herrle, J. Hardt, C. Galata, E. Birgin, N. Rahbari, N. Vassos (Mannheim University Medical Centre, Mannheim, Germany); M. G. Stoleriu*, R. Hatz (Asklepios Pulmonary Hospital, Munich Gauting, Germany); M. Albertsmeier*, N. Börner, C. Lampert, J. Werner (Ludwig-Maximilians-Universität Munich, Munich, Germany); B. Kuehlmann, L. Prantl (University Hospital Regensburg, Regensburg, Germany); S. M. Brunner*, H. J. Schlitt, F. Brennfleck, K. Pfister*, K. Oikonomou (University Medical Centre Regensburg, Regensburg, Germany); T. Reinhard, K. Nowak* (RoMed Klinikum Rosenheim, Rosenheim, Germany); U. Ronellenfitsch*, J. Kleeff, K. S. Delank, C. W. Michalski, G. Szabo (University Hospital Halle (Saale), Halle (Saale), Germany); R. Widyaningsih, G. A. Stavrou (Klinikum Saarbrücken, Saarbrücken, Germany); R. Bschorer*, J. Mielke, T. Peschel (Helios Kliniken Schwerin, Schwerin, Germany); A. Königsrainer*, M. Quante, M. W. Löffler, C. Yurttas (University Hospital Tübingen, Tübingen, Germany); J. Doerner*, R. Seiberth (Helios Universitätsklinikum Wuppertal, Wuppertal, Germany); K. Bouchagier*, S. Klimopoulos, D. Paspaliari, G. Stylianidis (Evaggelismos General Hospital, Athens, Greece); A. Syllaios, E. Baili, D. Schizas, T. Liakakos, A. Charalabopoulos*, C. Zografos, E. Spartalis (Laiko University Hospital, Athens, Greece); D. K. Manatakis*, N. Tasis, M. I. Antonopoulou (Athens Naval and Veterans Hospital, Athens, Greece); S. Xenaki*, E. Xynos*, E. Chrysos, E. Athanasakis, J. Tsiaousis (University Hospital of Heraklion Crete and Interclinic Hospital of Crete, Heraklion, Greece); E. Lostoridis*, P. Tourountzi (Kavala General Hospital, Kavala, Greece); G. Tzovaras*, K. Tepetes, D. Zacharoulis, I. Baloyiannis, K. Perivoliotis, J. Hajiioannou*, C. Korais, E. Gkrinia, C. E. Skoulakis, A. Saratziotis, O. Koukoura, D. Symeonidis, A. Diamantis (General University Hospital of Larissa, Larissa, Greece); G. Tsoulfas, C. D. Christou, A. Tooulias, V. Papadopoulos*, C. Anthoulakis, G. Grimbizis, D. Zouzoulas, D. Tsolakidis(Papageorgiou General Hospital, Thessaloniki, Greece); D. Tatsis, P. Christidis, L. Loutzidou, O. Ioannidis*, I. Astreidis, A. Antoniou, K. Antoniadis*, K. Vachtsevanos, K. Paraskevopoulos, I. Kalaitsidou, V. Alexoudi, A. Stavroglou, A. Mantevas, D. Michailidou, T. Grivas, D. Deligiannidis, S. Politis (George Papanikolaou General Hospital of Thessaloniki, Thessaloniki, Greece); A. Barrios Duarte*, A. L. Portilla, M. J. Lowey, G. Recinos, I. Lopez Muralles (Hospital General De Enfermedades, Guatemala City, Guatemala); M. A. Siguantay* (Hospital Roosevelt, Guatemala City, Guatemala); E. E. Estrada*, M. L. Aguilera-Arévalo*, J. M. Cojulun, G. Echeverría-Dávila (Hospital General San Juan De Dios, Guatemala City, Guatemala); C. Marín*, G. C. Icaza de Marín* (Hospital Regional De Zacapa, Zacapa, Guatemala); S. Y. Kok, H. K. M. Joeng, L. L. Chan, D. Lim (United Christian Hospital, Hong Kong); Z. Novak*, T. Echim (National Institute of Oncology, Budapest, Hungary); N. Suszták, B. Banky* (Szent Borbála Kórház, Tatabánya, Hungary); G. Kembuan, H. Pajan, A. A. Islam* (Rsud Wahidin Sudirohusodo, Jawa Timor, Indonesia); F. Rahim (Baghaei Hospital, Ahvaz, Iran); H. Safari (Golestan Hospital & Ahvaz Jundishapur University of Medical Sciences, Ahvaz, Iran); M. Mozafari, P. Brouki Milan*, A. Tizmaghz, M. Rezaei Tavirani (Firoozabadi Hospital, Tehran, Iran); A. Ahmed (Baghdad Medical City, Iraq); R. Hussein* (Zafaraniyah General Hospital, Baghdad, Iraq); C. Fleming, S. O'Brien, M. Y. Kayyal, A. Daly, S. Killeen, M. Corrigan* (Cork University Hospital, Cork, Ireland); J. De Marchi, A. Hill*, T. Farrell, N. F. Davis, D. Kearney, T. Nelson (Beaumont Hospital, Dublin, Ireland); P. J. Maguire*, C. Barry*, R. Farrell, L. A. Smith, H. M. Mohan, B. J. Mehigan, P. McCormick, J. O. Larkin*, B. A. Fahey, A. Rogers, N. Donlon, H. O'Sullivan, T. Nugent, J. V. Reynolds*, C. Donohue, P. Shokuhi, N. Ravi, C. Fitzgerald, P. Lennon, C. Timon, J. Kinsella, J. Smith, T. Boyle, D. Alazawi, E. Connolly, W. Butt, S. M. Croghan, R. P. Manecksha (St. James's Hospital, Dublin, Ireland); N. Fearon*, D. Winter*, H. Heneghan*, D. Maguire, T. Gallagher, K. Conlon, N. Kennedy, S. Martin, R. Kennelly, A. Hanly, K. C. Ng, J. Fagan, E. Geary, C. Cullinane, A. Hanly, E. Carrington, J. Geraghty, E. McDermott, R. Pritchard, D. McPartland, M. Boland, A. Stafford, D. Maguire, J. Geoghegan (St Vincent's University Hospital, Dublin, Ireland); J. A. Elliott*, P. F. Ridgway, A. E. Gillis, G. A. Bass, P. C. Neary, J. M. O'Riordan, D. O. Kavanagh, I. S. Reynolds, K. Conlon, D. P. Joyce, E. Boyle, B. Egan, M. Whelan, R. Elkady, S. Tierney, T. M. Connelly, H. Earley, M. Umair, C. O'Connell, R. P. Manecksha, A. Z. Thomas, D. Rice, A. Madden, Y. Bashir, B. Creavin (Tallaght Hospital, Dublin, Ireland); O. Cullivan, P. Owens, A. Canas-Martinez, C. Murphy, L. Pickett, B. Murphy, A. Mastrosimone, D. Beddy, M. Arumugasamy, M. Allen, M. Aremu* (Connolly Hospital Blanchardstown, Dublin, Ireland); C. McCarthy, C. O’Connor, D. B. O'Connor*, E. Kent, F. Malone, M. Geary (Rotunda Hospital, Dublin, Ireland); K. L. McKevitt*, A. J. Lowery*, É. J. Ryan, T. M. Aherne, A. Fowler, A. Hassanin, A. M. Hogan, C. G. Collins, L. Finnegan, P. A. Carroll, M. J. Kerin, S. R. Walsh (University Hospital Galway, Galway, Ireland); D. Nally*, C. Peirce, J. C. Coffey, R. M. Cunningham, S. Tormey (University Hospital Limerick, Limerick, Ireland); N. P. Hardy*, P. M. Neary (University Hospital Waterford, Waterford/University College Cork, Cork, Ireland); L. Muallem-Kalmovich, N. Kugler, R. Lavy, O. Zmora* (Shamir Medical Centre, Be'er Ya'akov, Israel); N. Horesh* (Sheba Medical Centre, Ramat Gan, Israel); R. Vergari* (Ospedali Riuniti Di Ancona, Ancona, Italy); S. Mochet*, R. Barmasse, A. Usai, L. Morelli (Ospedale Regionale Umberto Parini, Aosta, Italy); A. Picciariello*, V. Papagni, D. F. Altomare (Azienda Ospedaliero Universitaria Consorziale Policlinico Di Bari, Bari, Italy); M. Colledan*, M. F. Zambelli, S. Tornese, A. Camillo, E. Rausa*, F. Bianco, A. Lucianetti (Asst-Papa Giovanni Xxiii- Bergamo, Bergamo, Italy); G. M. Prucher*, A. M. Baietti, F. Ruggiero, P. Maremonti, F. Neri, S. Ricci, M. Biasini, A. G. Zarabini (Ospedale Maggiore/Bellaria Carlo Alberto Pizzardi Ausl Bologna, Bologna, Italy); A. Belvedere, P. Bernante, P. Bertoglio, S. Boussedra, E. Brunocilla, R. Cipriani, G. Cisternino, E. De Crescenzo, P. De Iaco, A. N. Della Gatta, G. Dondi, F. Frio, E. Jovine, F. Mineo Bianchi, J. Neri, D. Parlanti, A. M. Perrone, A. P. Pezzuto, M. Pignatti, G. Pilu, V. Pinto, G. Poggioli, M. Ravaioli, M. Rottoli*, R. Schiavina, M. Serenari, M. Serra, P. Solli*, M. Taffurelli, M. Tanzanu, M. Tesei, T. Violante, S. Zanotti (Sant'orsola Hospital, Alma Mater Studiorum University of Bologna, Bologna, Italy); V. Tonini*, L. Sartarelli, M. Cervellera, A. Gori (S.Orsola-Malpighi Hospital, Bologna, Italy); G. Armatura*, G. Scotton, S. Patauner, A. Frena (St. Moritz Hospital, Bologna, Italy); M. Podda*, A. Pisanu, G. Esposito, F. Frongia (Cagliari University Hospital, Cagliari, Italy); E. Abate, L. Laface, M. Casati*, M. Schiavo, T. Casiraghi (Ospedale Vittorio Emanuele III—Carate Brianza, Carate Brianza, Italy); G. Sammarco, G. Gallo*, G. Vescio, S. Fulginiti, V. Scorcia, G. Giannaccare, A. Carnevali (University ‘Magna Graecia’ of Catanzaro, Catanzaro, Italy); M. C. Giuffrida*, A. Marano, S. Palagi, S. Di Maria Grimaldi, V. Testa, C. Peluso, F. Borghi, A. Simonato, A. Puppo, M. D'Agruma, R. Chiarpenello, L. Pellegrino, F. Maione, D. Cianflocca, V. Pruiti Ciarello, G. Giraudo, E. Gelarda, E. Dalmasso, A. Abrate, A. Daniele, V. Ciriello, F. Rosato, A. Garnero, L. Leotta (Santa Croce E Carle Hospital, Cuneo, Italy); M. Giacometti*, S. Zonta (San Biagio Hospital, Domodossola—ASL VCO, Domodossola, Italy); D. Lomiento, L. Taglietti, S. Dester, B. Compagnoni, F. Viotti, R. Cazzaniga, R. Del Giudice (ASST Valcamonica Ospedale Di Esine, Esine, Italy); F. Mazzotti*, F. Pasini, G. Ugolini (Ospedale per gli Infermi di Faenza, Faenza, Italy); N. Fabbri, C. V. Feo*, E. Righini, S. Gennari (Azienda Unità Sanitaria Locale di Ferrara, Ferrara, Italy); M. Chiozza, G. Anania*, A. Urbani, M. Koleva Radica, P. Carcoforo*, M. Portinari, M. Sibilla (Azienda Ospedaliero Universitaria Sant'Anna, Ferrara, Italy); A. Anastasi, B. Bartalucci, A. Bellacci, G. Canonico*, L. Capezzuoli, C. Di Martino, P. Ipponi, C. Linari, M. Montelatici, T. Nelli, G. Spagni, L. Tirloni, A. Vitali (Ospedale San Giovanni Di Dio, Florence, Italy); C. Agostini, G. Alemanno, I. Bartolini, C. Bergamini, A. Bruscino, C. Checcucci, R. De Vincenti, A. Di Bella, M. Fambrini, L. Fortuna, G. Maltinti, P. Muiesan, F. Petraglia, P. Prosperi*, M. N. Ringressi, M. Risaliti, F. Sorbi*, A. Taddei* (Azienda Ospedaliera Universitaria Careggi, Florence, Italy); V. Lizzi*, F. Vovola, A. Arminio, A. Cotoia, A. L. Sarni (Ospedali Riuniti Azienda Ospedaliera Universitaria Foggia, Foggia, Italy); P. Familiari*, G. D'Andrea, V. Picotti, F. Bàmbina (Fabrizio Spaziani, Frosinone, Italy); T. Fontana* (Vittorio Emanuele, Italy); F. Barra*, S. Ferrero, C. Gustavino, C. Kratochwila, A. Ferraiolo, S. Costantini, P. Batistotti, A. Aprile, C. Almondo, L. Ball, C. Robba, S. Scabini, D. Pertile, A. Massobrio, D. Soriero (IRCCS Ospedale Policlinico San Martino, Genoa, Italy); S. D'Ugo*, N. Depalma, M. G. Spampinato (‘Vito Fazzi’ Hospital, Lecce, Italy); L. Lippa, C. Gambacciani, O. S. Santonocito*, F. Aquila, F. Pieri (Spedali Riuniti Di Livorno, Livorna, Italy); M. Ballabio*, P. Bisagni, M. Longhi, T. Armao, M. Madonini, A. Gagliano, P. Pizzini (Ospedale Maggiore Di Lodi, Lodi, Italy); A. Costanzi*, M. Confalonieri, M. Monteleone, G. Colletti, C. Frattaruolo, G. Mari (San Leopoldo Mandic, Padua, Italy); A. Spinelli*, G. Mercante, G. Spriano, F. Gaino, F. Ferreli, A. De Virgilio, V. Rossi, M. M. Carvello, F. Di Candido, H. Kurihara, E. Marrano, G. Torzilli, C. Castoro, F. M. Carrano (Humanitas Clinical and Research Centre—IRCCS, Milan, Italy); F. Martinelli, A. Macchi, M. Fiore*, S. Pasquali, S. P. B. Cioffi, M. Baia, C. Abatini, C. Sarre, A. Mosca, D. Biasoni, A. Gronchi, D. Citterio, V. Mazzaferro, P. Cadenelli, M. Gennaro, V. Capizzi, M. Guaglio, L. Sorrentino, G. Bogani, G. Sarpietro, L. Giannini, L. V. Comini, L. Rolli, S. Folli, F. Raspagliesi, C. Piazza, M. Cosimelli, R. Salvioni (Fondazione IRCCS Istituto Nazionale dei Tumori, Milan, Italy); B. Antonelli, L. Baldari, L. Boni, E. Cassinotti*, L. Pignataro, G. Rossi, S. Torretta, G. A. Beltramini, A. Gianni' (Fondazione IRCSS Ca' Granda—Ospedale Maggiore Policlinico, Milan, Italy); M. Tagliabue, R. De Berardinis, G. Pietrobon, F. Chu, S. Cenciarelli, L. Adamoli, M. Ansarin*, U. Fumagalli Romario*, F. Mastrilli (Istituto Europeo Di Oncologia—IRCCS, Milano, Italy); N. M. Mariani*, V. Nicastro (Asst Santi Paolo E Carlo, Milan, Italy); P. Cellerino* (Ospedale Fatebenefratelli E Oftalmico, Milan, Italy); F. Colombo*, A. Frontali, A. Bondurri, C. Guerci, A. Maffioli, L. Ferrario (Ospedale Luigi Sacco Milano, Milano, Italy); M. Candiani*, G. Bonavina, J. Ottolina, L. Valsecchi, P. Mortini, F. Gagliardi, M. Piloni, M. Medone, G. Negri, A. Bandiera, P. De Nardi, P. Sileri, M. Carlucci, D. Pelaggi, R. Rosati, A. Vignali, P. Parise, U. Elmore (San Raffaele Scientific Institute, Milan, Italy); N. Tamini*, L. C. Nespoli, M. Rennis, L. Pitoni, M. F. Chiappetta, E. Vico, R. Fruscio, T. Grassi (Ospedale San Gerardo, Università degli Studi di Milano Bicocca, Milan, Italy); D. Sasia*, M. Migliore, A. Gattolin, R. Rimonda, E. Travaglio, E. Olearo (Regina Montis Regalis Hospital, Mondovì, Italy); A. Tufo*, E. Marra, P. Maida, G. Marte, P. Tammaro (Ospedale Del Mare, Naples, Italy); F. Bianco*, P. Incollingo (Ospedale S. Leonardo—Asl Napoli 3 Sud, Castellammare Di Stabia, Naples, Italy); F. Izzo, A. Belli, R. Patrone, V. Albino, M. Leongito, V. Granata, M. Piccirillo, R. Palaia (Istituto Nazionale Tumori Fondazione, G. Pascale-IRCCS, Naples, Italy); E. Francone*, S. Gentilli, H. Nikaj (Azienda Ospedaliero Universitaria Maggiore Della Carità, Novara, Italy); A. Fiorini, C. Norcini, A. Chessa* (San Giovanni Di Dio, Italy); M. V. Marino*, A. Mirabella, G. Vaccarella (Azienda Ospedaliera, Ospedali Riuniti Villa Sofia-Cervello, Palermo, Italy); L. Musini, L. Ampollini*, M. Bergonzani, A. Varazzani*, L. Bellanti, M. Domenichini, G. Rossi, E. Cabrini, A. Fornasari, A. Freyrie*, D. O. Dejana, G. D'Angelo*, G. Bertoli, F. Di Lella, G. Bocchialini, M. Falcioni, D. Lanfranco, T. Poli (Azienda Ospedaliero-Universitaria di Parma, Parma, Italy); M. Giuffrida, A. Annicchiarico, G. Perrone, F. Catena* (Parma University Hospital, Parma, Italy); A. Raffaele* (Policlinico San Matteo, Pavia, Italy); A. De Manzoni Garberini* (Ospedale Civile Spirito Santo, Pescara, Italy); E. Baldini*, L. Conti, M. Ribolla, P. Capelli, S. M. Isolani, P. Maniscalco, M. Cauteruccio, C. Ciatti, C. Puma Pagliarello, S. Gattoni (Ospedale ‘Guglielmo Da Saliceto’, Piacenza, Italy); R. Galleano*, M. Malerba, M. Ciciliot (Ospedale Santa Corona, Pietra Ligure (SV), Pietra Ligure, Italy); F. Farnesi, M. Calabrò*, N. S. Pipitone Federico, E. G. Lunghi, A. Muratore (Edoardo Agnelli, Italy); L. Morelli*, G. Di Franco, M. Palmeri, D. Tartaglia*, F. Coccolini, M. Chiarugi, T. Simoncini*, A. Gadducci, M. Caretto, A. Giannini, A. Perutelli, L. Domenici*, S. Garibaldi, R. Capanna, L. Andreani, N. Furbetta, S. Guadagni, M. Bianchini, D. Gianardi (Azienda Ospedaliero Universitaria Pisana, Pisa, Italy); E. Pinotti*, M. Montuori, F. Carissimi, G. Baronio (Policlinico San Pietro, Ponte San Pietro, Italy); M. Zizzo*, C. Castro Ruiz, V. Annessi, M. T. Montella, G. Falco*, S. Mele, G. Ferrari, V. Mastrofilippo, V. D. Mandato*, L. Aguzzoli (Azienda Unità Sanitaria Locale—IRCCS Di Reggio Emilia, Reggio Emilia, Italy); C. Corbellini, C. Baldi*, G. M. Sampietro (Ospedale Di Rho—Asst Rhodense, Rho, Italy); G. M. Palini, N. Zanini*, G. Garulli (Ospedale Infermi di Rimini, Rimini, Italy); R. Barone*, A. Murgese, S. Mungo, M. Grasso, C. Marafante, S. L. Birolo, E. Moggia, M. Caccetta, A. Masciandaro, A. Deirino, M. Garino (Ospedale Degli Infermi Di Rivoli, Rivoli, Italy); R. Perinotti, F. Maiello (Ospedale degli Infermi, Biella, Italy); L. Gordini, C. P. Lombardi, F. Marzi, A. A. Marra, C. Ratto, M. Di Muro, F. Litta, V. De Simone, V. Cozza*, F. Rosa, A. Agnes, A. Parello, S. Alfieri, G. Sganga (Fondazione Policlinico Universitario Agostino Gemelli IRCCS, Rome, Italy); P. Lapolla*, A. Mingoli, G. De Toma, E. Fiori, F. La Torre, P. Sapienza, G. Brachini, B. Cirillo, I. Iannone, M. Zambon, A. Chiappini, S. Meneghini, G. B. Fonsi, P. M. Cicerchia, P. Bruzzaniti, A. Santoro, A. Frati, G. Marruzzo, D. Ribuffo (Policlinico Umberto I Sapienza University of Rome, Rome, Italy); A. Sagnotta*, L. Marino Cosentino, S. Mancini (Ospedale San Filippo Neri, Rome, Italy); G. Lisi*, D. Spoletini (Sant'Eugenio Hospital, Rome, Italy); V. Bellato, M. Campanelli, G. Sica*, L. Siragusa, (Policlinico Tor Vergata Hospital, Rome, Italy); L. Bonavina*, E. Asti, D. Bernardi, A. Lovece (IRCSS Policlinico San Donato, University of Milan, Milan, Italy); T. Perra, A. Porcu*, A. Fancellu, C. F. Feo, A. M. Scanu (Cliniche San Pietro, A.O.U. Sassari, Sassari, Italy); F. Tuminello, R. Galleano*, A. Franceschi, A. Langone (San Paolo, Italy); F. Fleres*, A. Spolini, P. Bordoni, M. Franzini, G. Clarizia, A. Grechi, A. Longhini (Ospedale Di Sondrio (Asst Valtellina E Alto Lario), Sondrio, Italy); E. Guaitoli, G. Manca (Perrino Hospital Brindisi, Italy); U. Grossi, S. Novello, G. Zanus*, M. Romano, S. Rossi (Ca' Foncello Treviso—DISCOG, Università di Padova, Padua, Italy); G. Pirozzolo*, A. Recordare (Dell'Angelo Hospital, ULSS3 Serenissima, Venezia, Italy); S. Paiella, G. Turri, S. Rattizzato, T. Campagnaro*, A. Guglielmi, C. Pedrazzani, A. Ruzzenente, E. Poletto, S. Conci, L. Casetti, M. Fontana, R. Salvia*, G. Malleo, A. Esposito, L. Landoni, M. De Pastena, C. Bassi, M. Tuveri, S. Nobile, G. Marchegiani, L. Bortolasi* (Azienda Ospedaliera Universitaria Integrata Di Verona, Verona, Italy); F. Ferrara (San Carlo Borromeo Hospital, ASST Santi Paolo e Carlo, Italy); M. La Torre (Fabia Mater Hospital, Rome, Italy); E. Sambugaro*, M. Malavolta*, G. Moretto, H. Impellizzeri, M. Inama* (Ospedale Pederzoli, Peschiera del Garda, Italy); G. Barugola, F. Ascari, G. Ruffo (IRCCS Ospedale Sacro Cuore Don Calabria, Negrar di Valpolicella, Verona, Italy); S. Granieri*, C. Cotsoglou (ASST Vimercate, Vimercate, Italy); M. Berselli*, M. Desio, V. Marchionini, E. Cocozza (ASST Settelaghi, Varese, Italy); S. Di Saverio*, G. Ietto, D. Iovino, G. Carcano (University of Insubria, Ospedale di Circolo e Fondazione Macchi, Varese, Italy); F. Ayasra*, A. Qasem, Y. Ayasra (Al-Basheer Hospital, Amman, Jordan); M. Al-Masri*, M. K. Abou Chaar, H. Al-Najjar, K. Ghandour, F. Alawneh, R. Abdel Jalil, S. Abdel Al, M. Elayyan, R. Ghanem, I. Lataifeh, O. Alsaraireh (King Hussein Cancer Centre, Amman, Jordan); F. J. Abu Za’nouneh, T. Fahmawee, A. Ibrahim, K. Obeidat* (King Abdullah University Hospital, Ar-Ramtha, Jordan); K. J. Lee (Keimyung University School of Medicine, Daegu, Korea); S. J. Shin, H. Chung (Keimyung University, Daegu, Korea); I. Albader, J. Alabbad, M. A. S. Albader (Mubarak Al-Kabeer Hospital, Jabriya, Kuwait); A. Bouhuwaish*, A. S. Taher, M. S. M. Omar (Tobruk Medical Centre, Tobruk, Libya); E. Abdulwahed*, M. Biala, M. Morgom (Tripoli Central Hospital, Tripoli, Libya); Elhadi A, Alarabi A, Msherghi A*, Elhajdawe F, Alsoufi A (Tripoli University Hospital, Libya); A. Salamah*, H. Salama, M. Bulugma*, H. Almabrouk (Zawia Teaching Hospital, Zawia, Libya); D. Venskutonis*, E. Dainius, E. Kubiliute, S. Bradulskis, A. Parseliunas, J. Kutkevicius, A. Subocius (Lithuanian University of Health Sciences Kaunas Clinical Hospital, Kaunas, Lithuania); Y. J. Cheong*, M. S. Masood* (Hospital Raja Permaisuri Bainun, Ipoh, Malaysia); C. W. Ngo*, R. Saravanan, N. Abdul Maei (Hospital Enche' Besar Hajjah Khalsom, Kluang, Malaysia); F. Hayati, N. Amin Sahid (Queen Elizabeth Hospital & Universiti Malaysia Sabah, Kota Kinabalu, Sabah, Malaysia); G. Yanowsky Reyes, J. Orozco Perez, R. Damian, R. Santana Ortiz, C. A. Colunga Tinajero (Antiguo Hospital Civil De Guadalajara, Guadalajara, Mexico); F. Cordera*, A. Gómez-Pedraza, A. Maffuz-Aziz, J. A. Posada, M. A. De la Rosa Abaroa, M. R. Alvarez, R. Arrangoiz, R. Hernández (ABC Medical Centre, Mexico); K. Bozada Gutierrez, M. Trejo-Avila*, C. Valenzuela-Salazar, J. Herrera-Esquivel, M. Moreno-Portillo (Hospital General Dr Manuel Gea González, Mexico City, Mexico); V. M. Pinto-Angulo*, E. E. Sosa-Duran, H. Ziad-Aboharp, X. Jimenez Villanueva (Hospital Juárez de México, Mexico City, Mexico); C. E. Soulé Martínez*, A. I. Lupián-Angulo (Hospital Central Norte Pemex, Mexico City, Mexico); J . J. Martínez Zarate*, E. Reyes Rodriguez, G. Montalvo Dominguez (Hospital Médica Sur, Mexico City, Mexico); F. C. Becerra García* (Hospital San Ángel Inn Chapultepec, Mexico City, Mexico); J. Melchor-Ruan, D. Vilar-Compte*, E. Romero-Bañuelos, A. Herrera-Gomez, A. Meneses-Garcia, D. Isla-Ortiz, R. A. Salcedo-Hernández, J. M. Hernández-Nava, J. E. Morales-Castelan (Instituto Nacional De Cancerologia, Mexico City, Mexico); C. Sarre, O. E. Posadas-Trujillo, G. A. Buerba, A. Alfaro-Goldaracena, E. Pena Gomez-Portugal, G. Lopez-Pena, C. A. Hinojosa*, M. A. Mercado* (Universidad Nacional Autonoma de Mexico, Instituto Nacional de Ciencias Medicas y Nutricion Salvador Zubiran, Mexico City, Mexico); A. Ramos-De la Medina*, L. Martinez, I. Duran, D. S. Gonzalez, M. J. Martinez, A. Nayen Sainz de la Fuente (Hospital Español Veracruz, Veracruz, Mexico); L. Miguelena, L. Hernández Miguelena (Hospital Regional Veracruz, Veracruz, Mexico); S. M. Louraoui*, A. El Azhari, M. Rghioui (Cheikh Khalifa International University Hospital, Casablanca, Morocco); E. Khya (Moulay Youssef, Casablanca, Morocco); A. Ghannam*, A. Souadka*, B. El Ahmadi, Z. H. Belkhadir, M. A. Majbar, A. Benkabbou, R. Mohsine (Institut National D'oncologie, Rabat, Morocco); M. Y. Oudrhiri*, H. Bechri, Y. Arkha, A. El Ouahabi (Centre Hospitalier Universitaire Ibn Sina Rabat, Rabat, Morocco); H. Frima*, S. Bachiri, L. C. Groen (Noordwest Ziekenhuisgroep, Alkmaar, Netherlands); T. Verhagen*, F. M. ter Brugge (Ziekenhuisgroep Twente, Almelo, Netherlands); J. C. G. Scheijmans, M. A. Boermeester*, R. Hompes*, E. M. Meima-van Praag, S. Sharabiany (Amsterdam UMC, Amsterdam, Netherlands); A. B. J. Borgstein, S. S. Gisbertz*, M. I. van Berge Henegouwen* (Amsterdam UMC VUMV, Amsterdam, Netherlands); S. Gans*, P. Van Duijvendijk, T. Herklots, T. De Hoop, M. R. De Graaff, D. Sloothaak, M. Bolster-van Eenennaam, J. Baaij, J. H. G. Klinkenbijl (Gelre ziekenhuis, Apeldoorn, Netherlands); R. Van Eekeren, E. J. Spillenaar Bilgen (Rijnstate, Arnhem, Netherlands); S. W. Van der Burg, N. J. Harlaar*, F. H. W. Jonker (Rode Kruis Ziekenhuis, Beverwijk, Netherlands); M. Vermaas, K. .R Voigt (Ijsselland Ziekenhuis, Capelle aan den Ijssel, Netherlands); D. Nellensteijn*, E. A. B. Bensi (Curacao Medical Center, Willemstad, Curaçao, Netherlands); L. Posma-Bouman* (Slingeland Ziekenhuis, Doetinchem, Netherlands); M. Van Sambeek*, M. Holscher (Catharina Ziekenhuis, Eindhoven, Netherlands); W. T. Van den den Broek (St. Anna Ziekenhuis, Geldrop, Netherlands); S. H. Kruijff*, J. P. P. M. De Vries, P. J. Steinkamp, P. K. C. Jonker, W. Y. Van der Plas, W. Bierman, Y. Janssen (University Medical Centre Groningen, Netherlands); J. Franken, S. Oosterling* (Spaarne Gasthuis, Netherlands); E. G. Boerma*, D. Schweitzer, M. H. F. Keulen, S. Ketting (Zuyderland MC, Sittard/Heerlen, Netherlands); J. A. Wegdam, T. S. de Vries Reilingh, E. Schipper, P. H. E. Teeuwen (Elkerliek Ziekenhuis, Helmond, Netherlands); E. R. Hendriks, A. A. W. Van Geloven (Tergooi Hospital, Hilversum, Netherlands); M. Emous*, R. Poelstra, M. Teunissen (Medisch Centrum Leeuwarden, Leeuwarden, Netherlands); S. L. Gerritsen, D. Boerma (St. Antonius Ziekenhuis, Nieuwegein, Netherlands); P. R. De Reuver*, F. Thunnissen, B. A. M. Vermeulen, A. Groen (Radboud Universitair Medisch Centrum, Nijmegen, Netherlands); T. M. Van Ginhoven*, C. L. Viëtor, M. J. W. van der Oest (Erasmus Medisch Centrum, Rotterdam, Netherlands); P. W. H. E. Vriens*, T. Houwen, J. Heisterkamp (Elisabeth TweeSteden Ziekenhuis, Tilburg, Netherlands); A. S. van Petersen, W. van der Meij, C. T. Stevens (Bernhoven, Uden, Netherlands); A. Pronk, W. J. Bakker (Diakonessenhuis, Utrecht, Netherlands); M. C. Richir*, M. R. Vriens, M. D. Filipe (University Medical Centre Utrecht, Utrecht, Netherlands); M. Uittenbogaart, W. K. G. Leclercq*, J. M. L. Sijmons, P. J. Vancoillie (Maxima Medical Centre, Eindhoven, Netherlands); J. Konsten*, M. Van Heinsbergen (VieCuri Medisch Centrum, Venlo, Netherlands); N. A. M. Dekker, F. C. den Boer (Zaans Medisch Centrum, Zaandam, Netherlands); A. Akinmade*, A. Adeyeye, E. Enoch, S. Fayose (Afe Babalola University Multi-System Hospital, Abuad, Nigeria); A. I. Okunlola*, A. A. Adeniyi, O. T. Adeyemo, I. O. Adebara, A. Bakare, O. F. Babalola, O. H. Abiyere, O. O. Banjo (Federal Teaching Hospital, Ido Ekiti, Nigeria); S. Olori*, O. G. Akaba, E. T. Agida, I. H. Abdullahi (University of Abuja Teaching Hospital, Abuja, Nigeria); I. K. Egbuchulem, D. Olulana, T. A. Lawal*, O. Ogundoyin, O. A. Oyelakin*, O. G. Nwaorgu, S. O. Sule (University College Hospital, Ibadan, Nigeria); C. C. Makwe, B. B. Afolabi, J. O. Seyi-Olajide*, A. O. Ademuyiwa, C. O. Bode, O. Atoyebi, O. A. Elebute, A. A. Okunowo (Lagos University Teaching Hospital, Lagos, Nigeria); O. M. Williams*, N. G. Eke, O. A. Oshodi, O. M. Faboya, A. S. Adeniran, O. A. Omisanjo, Y. A. Oshodi, A. A. Ogunyemi, K. M. Atobatele (Lagos State University Teaching Hospital, Lagos, Nigeria); A. Adeyeye, I. Aremu, O. Olasehinde, L. Abdur-Rahman*, J. Bello, A. Popoola, T. O. Sayomi, H. O. Raji, N. Adeleke, B. Lawal, O. Habeeb, O. Agodirin (University of Ilorin Teaching Hospital, Ilorin, Nigeria); M. A. Tolani*, T. T. Sholadoye*, S. E. Nwabuoku, M. Abubakar (Ahmadu Bello University Teaching Hospital, Zaria, Nigeria); T. Risteski*, V. Cvetanovska Naunova, L. Jovcheski, E. Lazova (University Clinic for Pediatric Surgery, Skopje, North Macedonia); I. Agledahl* (Hammerfest Hospital, Hammerfest, Norway); R. G. Breuer* (Soerlandet Hospital Kristiansand, Kristiansand, Norway); J. Massoud* (Khoula Hospital, Muscat, Oman); S. H. Waqar*, I. Rashid, A. Ayubi (Pakistan Institute of Medical Sciences, Islamabad, Pakistan); A. B. H. Bhatti* (Shifa International Hospital, Islamabad, Pakistan); M. U. Younis (Jinnah Postgraduate Medical Centre, Karachi, Pakistan); A. Ghouri, B. Ayub, R. H. Sayyed* (Patel Hospital, Karachi, Pakistan); A. Saleem, K. Turk*, A. Alvi, J. Abassy*, S. Khan, M. Arshad, K. Ahmed, T. Siddiqui, A. Pirzada (Aga Khan University, Karachi, Pakistan); A. A. Kerawala*, A. Jamal (Cancer Foundation Hospital, Karachi,Pakistan); L. Rai*, R. Nafees Ahmed, A. S. Memon* (Dr Ruth K.M. Pfau Civil Hospital, Karachi, Pakistan); A. U. Qureshi*, M. Ayyaz*, M. Umar, U. Butt*, M. Kashif, W. H. Khan*, M. Waris Farooka*, T. Wasim* (Services Hospital Lahore, Lahore, Pakistan); N. Talat, W. Tahir, J. Naseem (The Children's Hospital & The Institute of Child Health Lahore, Lahore, Pakistan); A. Akbar*, S. Afroze, L. Ali, A. Sultan, H. B. Ali (Doctors Hospital, Lahore, Pakistan); M. H. Janjua*, A. Janjua, S. Asghar, M. S. Farooq, M. Z. Sarwar, S. A. Naqi, K. M. Gondal (King Edward Medical University, Mayo Hospital, Lahore, Pakistan); S. I. Bukhari* (Lady Reading Hospital, Peshawar, Pakistan); M. Tariq (North West General Hospital and Research Centre, Peshawar, Pakistan); S. Javed*, E. Yaqoob, M. Ashraf, U. Mahmood, K. Raja Shabbir (Holy Family Hospital, Rawalpindi, Pakistan); S. A. Abukhalaf, A. Amro (Palestine Medical Complex, Ramallah, Palestine); J. M. Cabada Lee (Complejo Hospitalario Caja Seguro Social, Panama City, Panama); A. Aguilar (Hospital De Especialidades Pediatricas CSS, Panama City, Panama); E. Rodriguez (Hospital Santo Tomas, Panama City, Panama); K. Castillo (Hospital Irma De Lourdes Tzanetatos CSS, Panama City, Panama); M. Cukier*, H. Rodriguez-Zentner, E. Arrue (Pacifica Salud Hospital, Panama City, Panama); R. Isaacs Beron (Hospital Regional Rafael Hernandez CSS, Panama); A. Rodríguez Gonzalez (Hospital de Clínicas, Asuncion, Paraguay); V. Panduro-Correa*, D. K. Cornelio (Hospital Hermilio Valdizan Medrano, Huánuco, Peru); C. E. Otiniano Alvarado*, V. D. Caballero Sarabia (Arzobispo Loayza National Hospital, Lima, Peru); X. P. Vasquez-Ojeda, G. Lizzetti-Mendoza, M. Niquen-Jimenez, S. B. Shu-Yip, J. L. León Palacios, G. Borda-Luque* (Cayetano Heredia National Hospital, San Martín de Porres, Peru); S. A. Zegarra*, E. Huamán Egoávil, C. Suazo Carmelo (Guillermo Almenara National Hospital, La Victoria, Peru); R. Castro de la Mata*, D. Rivas, J. Targarona (Clínica Delgado Auna, Miraflores, Peru); Y. Trujillo*, M. Olivera Villanueva (Hospital Nacional Daniel Alcides Carrión, Bellavista, Peru); A. Lahoud-Velaochaga*, K. Cabillas, W. Castañeda, J. Colina Casas (Hospital Nacional Sergio E. Bernales, Lima, Peru); J. Betalleluz Pallardel*, F. Camacho Zacarías, E. Vélez Segura, D. L. Cruz Condori (Hospital De Emergencias Jose Casimiro Ulloa, Miraflores Peru); E. Huamán* (Hospital Guillermo Kaelin de la Fuente, Lima, Peru); R. Ugarte Oscco, C. Vergel Cabrera, E. Huamán Egoávil (Hospital Emergencia Ate Vitarte, Lima, Peru); Y. T. Carpio Colmenares*, L. A. García Barrionuevo, D. Cárdenas Ruiz de Castilla, P. Mansilla Doria, M. R. Li Valencia (Clínica El Golf—Sanna, San Isidro, Peru); A. Salazar, A. Sarmiento, C. Díaz, E. Morales, E. Ore, H. Zegarra, J. Siccha, M. Guardia, M. Sandoval, G. C. Mendiola*, M. Mimbela (Hospital Santa Rosa De Lima, Lima, Peru); R. Diaz-Ruiz*, L. A. Zeta, E. Cordova-Calle (Hospital Regional de Piura, Jose Cayetano Heredia, Piura, Peru); H. M. Nuñez (Hospital II-2 Tarapoto, Tarapoto, Peru); M. R. Ortiz-Argomedo* (Hospital Belén de Trujillo, Trujillo, La Libertad, Peru); J. Caballero-Alvarado, A. Salazar-Tantaleán, R. Espinoza-Llerena (Hospital Regional Docente de Trujillo, Trujillo, Peru); M. Aliaga-Ramos* (Hospital Alberto Hurtado Abadia, La Oroya—Yauli, Peru); O. Asodisen*, E. Jabagat, C. M. Tedoy (Cebu City Medical Center, Cebu City, Philippines); R. A Ramos, M. P. J. Lopez*, K. L. E. Violago, R. Aram (Ospital Ng Makati, Makati, Philippines); P. Carlos Santos, R. F. Filarca*, A. Carlos*, P. Santos, R. L. Filarca* (Medical Center Manila-ManilaMed, Manila, Philippines); E. J. Domingo, K. J. O. Khu, M. C. Lapitan, M. D. P. Sacdalan*, M. J. N. Kho, R. E. Baticulon, S. L. R. Bravo (Philippine General Hospital, Manila, Philippines); M. A. C. Cueto*, C. L. Ramos*, J. R. Fuentes (José R. Reyes Memorial Medical Centre, Manila, Philippines); H. Sadian, A. Gumarao, A. Barraquio, E. M. Cruz, A. D. Gonzales (Pasig City General Hospital, Pasig, Philippines); J. A. S. Reyes*, J. A. Salud, E. G. Tancinco, R. D. Rivera, J. A. Lim (The Medical City, Pasig, Philippines); J. C. Barcelon*, J. A. Chiu, M. I. Carballo (Cardinal Santos Medical Centre, San Juan, Philippines); P. Major*, I. Gawron*, R. Jach* (Jagiellonian University Medical College, Kraków, Poland); F. Borges*, P. Matos Costa, S. Henriques, S. C. Rodrigues (Hospital Garcia de Orta, Almada, Portugal); N. Gonçalves* (Hospital De Braga, Braga, Portugal); J. M. Curvas (Hospital de Bragança, Bragança, Portugal); A. Cabeleira, C. Branco, P. Serralheiro*, R. Alves, T. Teles (Hospital De Cascais—Dr José De Almeida, Alcabideche, Portugal); A. Lázaro*, C. Canhoto, J. Simões, M. Costa, A. C. Almeida, O. Nogueira, A. Oliveira, R. Athayde Nemésio, M. Silva, C. Lopes, M. J. Amaral, A. Valente da Costa, R. Andrade, R. Martins, A. Guimarães, P. Guerreiro, A. Ruivo, C. Camacho, M. Duque, E. Santos, D. Breda, J. M. Oliveira, A. L. De Oliveira Lopez, S. Garrido, M. Colino, J. De Barros, S. Correia, M. Rodrigues (Centro Hospitalar E Universitário De Coimbra, Coimbra, Portugal); P. Cardoso*, R. Martins, J. Teixeira, A. P. Soares, H. Morais*, R. Pereira, T. Revez, M. I. Manso, J. C. Domingues, P. Henriques, R. Ribeiro, V. I. Ribeiro, N. Cardoso, S. Sousa, G. Martins dos Santos (Centro Hospitalar Universitário do Algarve, Faro, Portugal); L. Carvalho, C. Osório, J. Antunes, S. Lourenço, P. Balau, M. Godinho, A. Pereira (Centro Hospitalar Entre o Douro e Vouga, Santa Maria da Feira, Portugal); N. Silva*, A. Kam da Silva Andrade, A. Pereira Rodrigues, N. Borges*, J. Correia, I. Vieira, T. Ribeiro, J. Catarino, R. Correia, F. Pais, R. Carreira Garcia, R. Bento, J. Cardoso, M. Luis, E. Santos, J. Henriques, J. Patena Forte, J. Maciel, J. Pinheiro Santos, M. Silva, T. P. Silva, A. Branquinho (Centro Hospitalar Universitário Lisboa Central, Lisbon, Portugal); A. Caiado* (Instituto Português De Oncologia De Lisboa Francisco Gentil, Lisbon, Portugal); P. Miranda, R. Garrido*, M. Peralta Ferreira, J. Ascensão, B. Costeira, C. Cunha, L. Rio Rodrigues, M. Sousa Fernandes, P. Azevedo, J. Ribeiro, I. Lourenço, H. Gomes, G. Mendinhos*, A. Nobre Pinto (Hospital Beatriz Angelo, Loures, Portugal); A. Ribeiro, C. G. Gil, C. Lima-da-Silva, C. Pereira, F. Tavares, I. Ferraz, J. I. Almeida, J. Marialva, L. Lopes, M. J. M. A. Costa, M. Nunes-Coelho, M. J. Teixeira, N. Machado, J. P. Alfonso, P. Saraiva, R. L. Silva, R. Santos, R. Almeida-Reis*, T. Correia-de-Sá, V. Fernandes, J. Almeida-Pinto, J. P. Gonçalves (Centro Hospitalar do Tâmega e Sousa, Penafiel, Portugal); H. Santos-Sousa*, S. Cavaleiro, A. M. Leite-Moreira, A. Pereira, A. Pereira-Neves, C. S. Faria, J. M. Monteiro, J. Nogueiro, M. Sampaio-Alves, M. Magalhães Maia, P. Vieira, T. Pina-Vaz, F. Jácome, V. Devezas, A. Almeida, H. Silveira, S. Vaz, S. Castanheira Rodrigues (Centro Hospitalar E Universitário De São João, Porto, Portugal); D. Costa Santos, J. V. Grilo, A. Abreu da Silva*, M. Claro, A. C. Deus (Hospital Do Litoral Alentejano, Santiago do Cacém, Portugal); R. Branquinho* (Centro Hospitalar Medio Tejo, Portugal); P. M. D. D. Santos*, B. Patrício, A. C. Vieira Paiva Lopes (Hospital De Torres Vedras—Centro Hospitalar Do Oeste, Torres Vedras, Portugal); J. M. Mendes, M. F. Carvalho, C. M. Oliveira* (Centro Hospitalar do Médio Ave, Santo Tirso, Portugal); A. Tojal*, J. Pinto (Centro Hospitalar Tondela-Viseu, Viseu, Portugal); A. Abutaka, A. Zarour*, M. Abdelkareem, S. M. Ali, M. Al Tarakji, R. Alfkey, K. Mukhtar, I. R. Wani, R. Singh, K. Ahmed, N. Bouchiba, H. Mahdi, S. Abdelaziem Mustafa, A. Al Ansari (Hamad General Hospital, Doha, Qatar); R. Drasovean, A. Caziuc (Clinica Chirurgie I, Spitalul Clinic Judetean de Urgenta, Cluj-Napoca, Romania); E. Galliamov, M. Agapov*, V. Kakotkin, E. Semina, V. Kubyshkin, A. Kamalov (Moscow Research and Educational Centre, Moscow, Russian Federation); S. K. Efetov, V. S. Kochetkov, T. Garmanova, P. Tsarkov*, I. Tulina, S. Rodimov, D. Markaryan, E. Kazachenko (Clinic of Coloproctology and Minimally Invasive Surgery, Moscow, Russian Federation); A. Yanishev*, A. Abelevich, A. Bazaev, A. Kokobelyan, P. Zarubenko (Nizhny Novgorod Regional Clinical Hospital, Nizhny Novgorod, Russian Federation); A. Zakharenko*, A. Novikova (Pavlov First State Medical University of St. Petersburg, St. Petersburg, Russian Federation); G. Kim*, D. Shmatov, M. Stoliarov, M. Kamenskikh (Saint Petersburg State University Hospital, Saint Petersburg, Russian Federation); G. Nambi (University Hospital, Saudi Arabia); A. S. Almulhim, T. Madkhali, A. Alzouhir, A. Alissa (King Fahad Hospital Hofuf, Al Hofuf, Saudi Arabia); E. Alameer*, M. Badedi, A. Q. Alnami, H. Darraj (Jazan University, Jazan, Saudi Arabia); A. Alkhuzaie*, F. Khadwardi, M. Abualjadayel, W. Tashkandi (King Abdulaziz Hospital and Oncology Centre, Jeddah, Saudi Arabia); A. Farsi, N. Malibary*, N. Trabulsi, S. Farsi (King Abdulaziz University Hospital, Jeddah, Saudi Arabia); A. Said Bayazeed*, M. Nasser*, M. S. Siddiqui*, S. Al Awwad* (King Fahad General Hospital, Jeddah, Saudi Arabia); M. Alshahrani, F. Alsharif, M. W. Fahmi (Aseer Central Hospital, Saudi Arabia); A. Gudal*, A. Alasmari, S. Alqahtani (King Abdullah Medical Complex, Jeddah, Saudi Arabia); S. Majrashi*, A. Mashat, R. Al Raddadi (East Jeddah General Hospital, Jeddah, Saudi Arabia); A. Alharbi*, Y. Nasser, H. Hamayel, A. Alhojaili, R. Aljohani (King Fahad General Hospital, Jeddah, Saudi Arabia); O. Sogair* (Ohud Hospital, Medina, Saudi Arabia); O. Alfarhan, A. Alzahrani, B. Alzomaili*, W. Tashkandi (Hera General Hospital, Mecca, Saudi Arabia); M. A. Azab* (King Abdullah Medical City, Mecca, Saudi Arabia); M. Alotaibi*, A. Maashi, A. Zowgar, M. Alsakkaf (King Faisal Hospital, Saudi Arabia); M. Alnemary*, S. Khayat, S. Felmban, A. Almhmadi (Alnoor Specialist Hospital, Mecca, Saudi Arabia); M. Alqannas, D. Cortés Guiral, M. Alyami*, A. Elawad (King Khalid Hospital, Najran, Saudi Arabia); A. Alhefdhi*, F. Alresaini, W. Kurdi, M. Tulbah, M. Aldakheel, N. Alsahan, S. Koussayer, H. Elsheikh, M. Al-qattan, S. Alshanafey, A. Rafique, N. Mahabbat, B. Saeed (King Faisal Specialist Hospital, Riyadh, Saudi Arabia); E. Al-Kharashi*, K. Alsowaina, N. Arab, F. Aljaber, I. Al Hasan, A. Alghamdi, F. Badahdah, A. Alghuliga, F. Abdulfattah, F. Alanazi, F. Albaqami, A. Alsuhaibani (Prince Sultan Military Medical City, Riyadh, Saudi Arabia); A. AlFakhri*, S. Alqasem, N. Alajaji (King Fahad Medical City, Riyadh, Saudi Arabia); T. Nouh*, A. Bin Nasser, J. Alowais, A. Alburakan, O. Alamri, A. Albdah, K. Alawi, M. Alshalhoub (King Saud University, Riyadh, Saudi Arabia); K. ElSanhoury*, A. Almofarreh, S. Ibrahim, H. Elshafie, I. Osman, T. Guzman, H. Mutair, A. Siddiqui, S. Chowdhury*, R. Alghamdi, S. Almutrafi, J. Alfaifi, J. D'Souza, A. Alshitwi, N. Alkreedees, M. Alramadhan, M. Alshehri (King Saud Medical City, Riyadh, Saudi Arabia); A. Alzahrani*, S. Alobaysi, H. Badr, A. Alshahrani (Security Forces Hospital, Saudi Arabia); A. Alshehri*, M. Alrashed, T. Altahan, T. Alsabahi, R. Alhossaini, M. Sbaih (Prince Mohammed Bin Abdulaziz Hospital, Riyadh, Saudi Arabia); Y. Alalawi, K. Alnwijy, A. Al Ayed (King Salman Armed Forces Hospital, Tabouk, Saudi Arabia); S. Ghedan, R. Alharthi, S. Awad*, M. Sharara, S. Abdelrhman, W. Althobaiti (King Faisal Medical Complex, Riyadh, Saudi Arabia); L. Srbinovic, M. Perovic, Z. Mikovic, B. Nikolic, M. Vasiljevic, V. Pazin, V. Mandic Markovic, D. Dimitrijevic, N. Zecevic (Clinic for Gynecology and Obstetrics Narodni Front, Belgrade, Serbia); P. Gregoric*, D. Micic, Z. Loncar, K. Doklestic, N. Ivancevic (Clinical Centre of Serbia, Belgrade, Serbia); V. Djukic*, D. Stojakov, R. Ilic, P. Savic, N. Pijanovic, M. Milanovic, M. Radosavljevic, T. Dejanovic, M. Kostic, J. Paskas, S. Bojic, P. Stevanovic, M. Djuric (KBC Dr Dragisa Misovic-Dedinje, Belgrade, Serbia); M. Kadija*, G. Tulic, I. Glisovic Jovanovic (Clinic for Orthopaedic Surgery and Traumatology, Belgrade, Serbia); B. Lieske* (National University Hospital, Singapore, Singapore); E. Kayombo, I. Kruger, M. De Kock, A. Malan, C. Ferreira, H. Du Preez, W. Mulder, C. Noel*, S. Le Grange, O. Lusawana (Universitas Academic Hospital Complex, Bloemfontein, South Africa); C. Kies, E. Steyn, J. Janson, J. J. P. Buitendag, K. Chu*, M. Mihalik, R. Nel, S. Naidoo (Tygerberg Hospital, Cape Town, South Africa); C. Kloppers*, D. Nel, E. Jonas, H. Pickard, M. Bernon, N. Almgla*, S. Rayamajhi, W. Mugla* (Groote Schuur Hospital, Cape Town, South Africa); C. Carapinha (Netcare Clinton Hospital, Johannesburg, South Africa); G. Y. Hyman, M. Fourtounas, R. Moore* (Chris Hani Baragwanath Academic Hospital, Johannesburg, South Africa); A. Sánchez Mozo (Complejo Hospitalario Universitario De Albacete, Albacete, Spain); H. Aguado Lopez* (Hellin Hospital, Hellin, Spain); A. Zárate Pinedo (Hospital Germans Trias i Pujol, Badalona, Spain); M. Jimenez Toscano*, N. Alonso de la Fuente, G. Mancebo, L. Cecchini, M. Munarriz, M. Cazador Labat, A. López Campillo, P. Martorell, C. A. Espinosa (Hospital Del Mar, Barcelona, Spain); P. Caja Vivancos*, I. Villalabeitia Ateca*, M. Prieto Calvo, H. Marín, P. Martin Playa*, A. Gainza, E. J. Aragon Achig, A. Rodriguez Fraga, I. Melchor Corcóstegui, G. Mallabiabarrena Ormaechea*, J. J. Garcia Gutierrez, L. Barbier, M. A. Pesántez Peralta*, M. Jiménez Jiménez, J. A. Municio Martín, J. Gómez Suárez, G. García Operé, L. A. Pascua Gómez, M. Oñate Aguirre (Hospital Universitario Cruces, Barakaldo, Spain); A. Fernandez-Colorado, M. De la Rosa-Estadella*, A. Gasulla-Rodriguez, M. Serrano-Martin, A. Peig-Font, S. Junca-Marti, M. Juarez-Pomes, S. Garrido-Ondono, L. Blasco-Torres, M. Molina-Corbacho, Y. Maldonado-Sotoca, A. Gasset-Teixidor, J. Blasco-Moreu (Corporació Sanitària Parc Taulí, Barcelona, Spain); L. Gomez Fernandez*, L. Cayetano Paniagua (Consorci Sanitari De Terrassa, Terrassa, Spain); O. Izquierdo, D. Ventura, J. Castellanos (Parc Sanitari Sant Joan de Déu, Barcelona, Spain); E. Ballester Vazquez, A. Sanchez Lopez*, C. Balague Ponz, E. M. Targarona Soler, S. Sanchez Cabús, V. Molina Santos, J. A. Gonzalez Lopez, R. Medrano Caviedes, A. Moral Duarte (Hospital De La Santa Creu I Sant Pau, Barcelona, Spain); E. Espin-Basany*, G. Pellino, R. Blanco-Colino (Vall D'Hebron University Hospital, Barcelona, Spain); V. Turrado-Rodriguez*, A. M. Lacy, F. B. de Lacy, X. Morales, A. Carreras-Castañer*, P. Torner, M. Jornet-Gibert, M. Balaguer-Castro, M. Renau-Cerrillo, P. Camacho-Carrasco, M. Vives-Barquiel, B. Campuzano-Bitterling, I. Gracia, R. Pujol-Muncunill (Hospital Clínic de Barcelona, Barcelona, Spain); O. Martin-Sole*, J. Rubio-Palau, X. Tarrado, L. Garcia-Aparicio, I. De Haro Jorge, A. Martin, J. Rojas-Ticona, S. Perez-Bertolez, M. Cuesta Argos, B. Capdevila Vilaro, M. Coronas Soucheiron, M. Riba Martinez, L. Saura Garcia, J. Prat Ortells, M. Bejarano Serrano, P. Parri, C. Massaguer, F. Vicario, P. Palazon Bellver, I. Moraleda Gudayol(Hospital Sant Joan de Deu de Barcelona, Barcelona, Spain); A. Lara, D. Escobar, M. Arrieta, U. Garcia de Cortazar*, I. Villamor Garcia (Hospital Universitario De Basurto, Bilbao, Spain); A. Landaluce-Olavarria*, M. Gonzalez De Miguel, L. Fernández Gómez Cruzado, E. Begoña, D. Lecumberri (Hospital Urduliz, Urduliz, Spain); M. A. Acosta Mérida*, A. F. Yepes Cano* (Hospital Universitario De Gran Canaria Doctor Negrín, Las Palmas, Spain); M. Estaire Gómez*, D. Padilla-Valverde*, S. Sánchez-García, D. Sanchez-Pelaez, E. Jimenez Higuera, R. Picón Rodríguez, À. Fernández Camuñas, C. Martínez-Pinedo, E. P. Garcia Santos, V. Muñoz-Atienza, A. Moreno Pérez, C. A. López de la Manzanara Cano (Hospital General Universitario De Ciudad Real, Ciudad Real, Spain); B. Ugarte-Sierra*, F. J. Ibáñez-Aguirre, U. De Andres Olabarria, F. J. Fernández Pablos, M. Durán Ballesteros, A. Sanz Larrainzar (Hospital Universitario De Galdakao, Galdakao, Spain); V. Jiménez Carneros*, A. Valle Rubio, L. Alonso-Lamberti, A. Salazar, J. García-Quijada, R. Leon, J. L. Rodriguez, J. Jimenez Miramón, J. M. Jover, M. B. Martín Salamanca*, M. Assaf, V. Pérez Simón, S. A. Landeo Agüero, N. Baeza Pintado, M. A. Huertas Fernandez, A. Carabias (Getafe University Hospital, Madrid, Spain); M. V. Sosa*, P. Lora-Cumplido, L. Lanuza (Hospital de Cabueñes, Gijón, Spain); M. Galipienso Eri, J. D. Garcia Montesino, J. Dellonder Frigolé, D. Noriego Muñoz* (Hospital Universitari De Girona Dr Josep Trueta, Girona, Spain); A. Navarro-Sánchez* (Complejo Hospitalario Universitario Insular-Materno Infantil de Gran Canaria, Las Palmas, Spain); D. Enjuto*, M. Perez Gonzalez, P. Díaz Peña, J. Gonzalez, M. Marqueta De Salas, P. Martinez Pascual, L. Rodríguez Gómez, R. Garcés García, A. Ramos Bonilla, N. Herrera-Merino, P. Fernández Bernabé, E. P. Cagigal Ortega, I. Hernández, E. García de Castro Rubio, I. Cervera (Severo Ochoa University Hospital, Spain); M. Espino Segura-Illa, G. Sánchez Aniceto*, A. M. Castaño-Leon*, L. Jimenez-Roldan, J. Delgado Fernandez, A. Pérez Núñez, A. Lagares, D. Garcia Perez, M. Santas, I. Paredes, O. Esteban Sinovas, L. Moreno-Gomez, E. Rubio*, V. Vega, A. Vivas Lopez, M. Labalde Martinez, O. García Villar, P. M. Pelaéz Torres, J. Garcia-Borda, E. Ferrero Herrero, P. Gomez, C. Eiriz Fernandez, C. Ojeda-Thies*, J. M. Pardo Garcia (12 De Octubre University Hospital, Madrid, Spain); M. Di Martino*, A. De la Hoz Rodriguez, J. García Septiem, R. Maqueda González, L. Delgado Búrdalo, A. Correa Bonito, E. Martin-Perez (Hospital Universitario De La Princesa, Madrid, Spain); J. E. García Villayzán*, B. Albi Martin (Fundación Jimenez Diaz University Hospital, Madrid, Spain); P. Lozano Lominchar, L. Martin, M. Fernadez, C. Rey-Valcarcel*, M. Tousidonis, L. Martin-Albo Caballero, A. Lowy, P. Alonso Ortuño, E. Ayuso Herrera, J. Caño Velasco, J. Aragon-Chamizo, M. D. Perez Diaz, O. Mateo-Sierra, B. Quintana-Villamandos, J. M. Barrio, M. Fanjul, C. Sanchez-Perez, M. L. Fernandez, S. Hernandez-Kakauridze, J. Rio (Hospital General Universitario Gregorio Marañón, Madrid, Spain); D. Díaz Pérez, J. Serrano González*, L. Colao García, M. Gutierrez Samaniego, M. A. Hernandez Bartolome, P. Galindo Jara, E. Esteban Agustí (Hospital Universitario De Torrejón De Ardoz, Torrejón De Ardoz, Spain); J. Ripollés-Melchor*, A. Abad-Motos, A. Abad-Gurumeta, E. Martínez-Hurtado, A. Ruiz-Escobar (Infanta Leonor University Hospital, Madrid, Spain); N. Brogly, E. Guasch, A. Hernandez Gutierrez, J. L. Bartha Rasero, Y. Perez, V. Garcia-Pineda, M. Gracia, J. Siegrist Ridruejo, M. D. Diestro, J. I. Sanchez-Mendez, C. Marti, M. Melendez, E. Moreno-Palacios, A. Loayza, L. Frias, I. Zapardiel*, I. Rubio-Perez*, M. I. Prieto Nieto, J. Guevara, A. Gegundez Simon, S. Gortazar, N. Chavarrias, E. Alvarez, J. Saavedra, P. Ramos-Martin, A. Urbieta, J. Gomez Rivas, C. Toribio-Vázquez, A. Yebes (Hospital Universitario La Paz, Madrid, Spain); M. Hernández-García, M. Losada*, B. Diéguez, M. García-Conde, A. Alonso Poza (Hospital Universitario Del Sureste, Madrid, Spain); L. Marquez*, R. Becerra, M. Martin, T. Jorgensen (Hospital Central De La Cruz Roja San Jose Y Santa Adela, Madrid, Spain); J. M. Muguerza*, J. Dziakova, C. Sánchez del Pueblo, P. Saez Carlin, E. Camarero, S. Picazo, M. J. Pizarro, R. Avellana, V. Catalán, L. Lopez Antoñanzas, O. Cano, R. Anula, R. Sanz Lopez, G. Sanz Ortega, M. Garcia Alonso, A. J. Torres, E. Martin Antona, S. Garcia Botella (Hospital Clínico San Carlos De Madrid, Madrid, Spain); D. Ramos*, A. G. Barranquero, J. Ocaña, J. Núñez, C. Cerro Zaballos (Hospital Universitario Ramón Y Cajal, Madrid, Spain); D. Crego-Vita*, M. Huecas-Martinez (Hospital Central De La Defensa Gomez Ulla, Madrid, Spain); L. Sánchez-Guillén, A. Fernández-Candela, C. Curtis-Martínez, Á. Soler-Silva, I. Oller, D. Triguero-Cánovas, M. Bosch-Ramírez, C. Lillo, S. Lario, A. Arroyo (Universidad Miguel Hernández, Elche, Alicante, Spain); M. Diez Alonso*, F. Mendoza-Moreno, C. Vera Mansilla, E. Ovejero Merino, P. Hernandez, A. Blazquez Martin, F. Ruiz Grande, N. Morales Palacios*, E. Garcia-Loarte Gomez, E. Garca Rico (Hospital Universitario Principe De Asturias, Madrid, Spain); A. M. Minaya-Bravo*, C. San Miguel Méndez, A. Galvan Pérez, E. Gonzalez-Gonzalez, A. Robin Valle de Lersundi, E. Calcerrada Alises, M. A. García-Ureña, A. Cruz Cidoncha (Hospital Del Henares, Madrid, Spain); P. Troncoso Pereira*, F. Alcaide Matas, J. M. García Pérez (Hospital Mateu Orfila, Mahón, Spain); J. M. Muñoz Vives, A. Osorio, C. J. Gómez Díaz, C. A. Guariglia, C. Soto Montesinos*, L. Sanchon, M. Xicola Martínez, N. Guàrdia, P. Collera, R. Diaz Del Gobbo, R. Sanchez Jimenez, R. Farre Font, R. Flores Clotet (Fundació Althaia—Xarxa Assistencial Universitària de Manresa, Manresa, Spain); P. Calvo Espino*, P. Guillamot Ruano (Hospital Universitario De Móstoles, Madrid, Spain); J. Rey-Biel*, L. Pingarrón-Martin, I. Ruiz Martin, C. Moliner Sanchez (Rey Juan Carlos University Hospital, Madrid, Spain); M. Carrasco-Prats*, C. Giménez-Francés, M. Ruiz-Marín, A. J. Fernández-López, D. García-Escudero, V. García-Porcel, R. Lax-Pérez, M. Sánchez-Robles, M. Valero-Soriano, E. Medina-Manuel, V. García-Soria, E. Gurrea-Almela, A. Marco-Garrido, J. A. Martínez-Alonso, F. M. González-Valverde, P. V. Fernández-Fernández, C. Sánchez-Rodríguez (Hospital General Reina Sofía, Murcia, Spain); J. Aguilar-Jimenez*, M. Baeza-Murcia, J. L. Aguayo-Albasini (Morales Meseguer University Hospital, Murcia, Spain); T. Nicolás-López, F. Alconchel* (Hospital Clínico Universitario Virgen de la Arrixaca (IMIB-Arrixaca), Murcia, Spain); D. Fernández Martínez, L. Solar-Garcia, L. J. García Flórez* (Hospital Universitario Central De Asturias (Huca), Oviedo, Spain); H. Llaquet Bayo* (Hospital de Palamós-SSIBE, Palamós, Spain); N. Pujol-Cano, J. J. Segura-Sampedro*, C. Soldevila-Verdeguer, S. Jeri-McFarlane, A. Gil-Catalan, A. Craus-Miguel, L. Cruz, P. Valente, M. Afonso-Garcia, E. Ferrer-Inaebnit, A. Oseira-Reigosa, L. Fernandez-Vega, B. Villalonga-Ramirez, F. X. Gonzalez Argente (Son Espases University Hospital, Palma, Spain); I. Mora-Guzmán* (Hospital Santa Bárbara, Soria, Spain); F. J. Landete Molina*, F. J. Morera Ocón, E. Canelles Corell (Hospital General Asociado Universitario de Requena, Valencia, Spain); M. T. Gavaldà Pellicé*, J. R. Salinas Peña, P. Cavallé Busquets (Hospital Universitari Sant Joan, Reus, Spain); J. Trebol*, A. B. Sánchez-Casado, L. Munoz-Bellvis (Complejo Asistencial Universitario De Salamanca, Salamanca, Spain); L. E. Pérez-Sánchez*, V. Concepción Martín, A. Díaz García, M. Vallve-Bernal (Hospital Universitario Nuestra Señora De Candelaria, Santa Cruz de Tenerife, Spain); A. Calvo Rey, G. M. Prada Hervella*, L. Dos Santos Carregal, M. I. Rodriguez Fernandez, M. Freijeiro, S. El Drubi Vega (Hospital Clinico Universitario De Santiago De Compostela (Chus), Santiago De Compostela, Spain); A. L. Picardo, A. Cuadrado-Garcia, D. Serralta de Colsa, J. A. Rojo Lopez, F. Sanchez Cabezudo Noguera, I. Ortega Vazquez*, L. Garcia-Sancho Tellez, P. Mato, J. Heras Aznar (Infanta Sofia University Hospital, Madrid, Spain); J. Jimeno Fraile*, D. Morales-Garcia, M. Carrillo-Rivas, E. Toledo Martínez, À. Pascual (Marqués De Valdecilla University Hospital, Santander, Spain); A. Senent-Boza*, A. Sánchez-Arteaga, I. Benítez-Linero, F. Manresa-Manresa, L. Tallón-Aguilar, L. Melero-Cortés, M. R. Fernández-Marín, V. M. Durán-Muñoz-Cruzado, I. Ramallo-Solís, P. Beltrán-Miranda, F. Pareja-Ciuró, B. T. Antón-Eguía (Hospital Universitario Virgen Del Rocío, Seville, Spain); F. Oliva Mompean, J. Gomez-Rosado*, J. Reguera-Rosal, J. Valdes-Hernandez, L. Capitan-Morales, M. D. del Toro Lopez (Hospital Universitario Virgen Macarena, Seville, Spain); M. Achalandabaso Boira*, R. Memba Ikuga, M. Abellán, R. Sales, C. Olona, R. Jorba (Hospital Universitari De Tarragona Joan XXIII, Tarragona, Spain); J. Hernandez Gutierrez, A. Tébar Zamora* (Complejo Hospitalario De Toledo, Toledo, Spain); J. Sancho-Muriel*, H. Cholewa, M. Frasson (Hospital Universitario Y Politécnico La Fe, Valencia, Spain); J. Domenech, A. Roselló Añón*, M. J. Sangüesa (Hospital Arnau De Vilanova, Valencia, Spain); D. Moro-Valdezate*, M. Garcés-Albir*, F. Lopez* (Hospital Clínico Universitario de Valencia, Valencia, Spain); J. C. Bernal-Sprekelsen*, J. C. Catalá Bauset, P. Renovell Ferrer, C. Martínez Pérez, O. Gil-Albarova, J. Gilabert Estellés, K. Aghababyan (Consorcio Hospital General Universitario, Valencia, Spain); B. De Andrés-Asenjo*, J. Beltrán de Heredia, A. Vázquez-Fernández, F. J. Ortiz de Solorzano-Aurusa, J. Trujillo-Díaz, M. Ruiz-Soriano, C. Jezieniecki, T. Gómez-Sanz, H. Núñez-Del Barrio, A. Romero-De Diego, V. García-Virto, H. J. Aguado (Hospital Clínico Universitario De Valladolid, Valladolid, Spain); M. T. Fernández Martín* (Hospital Medina Del Campo, Valladolid, Spain); F. J. Tejero-Pintor*, B. Pérez-Saborido, E. Choolani Bhojwani, F. Acebes García, P. Marcos-Santos, A.D. Bueno Cañones, J. Sanchez Gonzalez, M. Toledano, M. Bailón, D. Pacheco Sánchez (Hospital Universitario Río Hortega, Valladolid, Spain); M. Paniagua Garcia Senorans*, R. Sanchez-Santos (Álvaro Cunqueiro Hospital, Vigo, Spain); A. Vazquez Melero*, D. Garcia, E. Díez, I. Herrero, I. M. Soeda, M. Camuera, M. Balluerca, M. Sánchez-Rubio, P. Paunero Vazquez (Hospital Universitario Araba, Araba, Spain); A. Martinez-German, C. Gracia-Roche, I. Gascon-Ferrer, V. Duque-Mallen*, M. D. C. De Miguel-Ardevines, N. Sanchez-Fuentes, M. S. Santero-Ramirez, M. Matute-Najarro, M. Herrero-Lopez, M. Sanchez-Rubio, M. Cantalejo-Diaz, M. T. Gonzalez-Nicolas-Trebol, S. Saudi-Moro, U. M. Jariod-Ferrer (Hospital Universitario Miguel Servet, Zaragoza, Spain); R. Rivas, F. Rivas (Hospital Clinico Universitario Zaragoza, Zaragoza, Spain); J. Escartin*, J. L. Blas Laina, A. Nogués, B. Cros, I. Talal El-Abur, J. Garcia Egea, C. Yanez (Hospital Royo Villanova, Zaragoza, Spain); U. Jayarajah*, S. Ravindrakumar, V. S. D. Rodrigo, A. Arulanantham, G. B. K. D. Bandara (District General Hospital Chilaw, Chilaw, Sri Lanka); H. K. S. Hamid, E. E. Ali, A. B. H. Widatalla, I. Bakheit*, M. Awadelkarim (Ibrahim Malik Teaching Hospital, Khartoum, Sudan); A. A. Ali Karar (Al-Rajhi, Sudan); M. Saleh (University of Gezira Hospital, Wad Madani, Sudan); H. Taflin* (Sahlgrenska University Hospital, Sahlgrenska, Sweden); P. Myrelid*, L. Amorim Braz (Linköping University Hospital, Linköping, Sweden); L. Hagander*, M. Hambraeus, E. Omling, M. Salö (Lund University, Skåne University Hospital, Lund, Sweden); S. Arkani*, J. Freedman* (Danderyds Hospital, Danderyd, Sweden); C. Montan, E. K. Lindqvist*, P. Elbe, R. Hultgren (Karolinska University Hospital, Solna, Sweden); A, Älgå*, M, Nordberg, G, Sandblom (South General Hospital, Stockholm, Sweden); M. Rutegård*, F. Holmner, M. Sund*, N. Löfgren (Umeå University Hospital, Umeå, Sweden); A. Tampakis, O. Kollmar, M. von Fluee (University Hospital Basel, Basel, Switzerland); A. Balaphas*, C. Toso, N. Colucci, S. G. Popeskou (Geneva University Hospitals, Geneva, Switzerland); M, Gass*, A, Scheiwiller, J, Metzger (Luzerner Kantonsspital, Lucerne, Switzerland); E. Gialamas, M. Chevallay, M. Sauvain*, O. Dwidar (Pourtales Neuchâtel Hospital, Neuchâtel, Switzerland); S. Y. Kiessling, S. J. Stoeckli* (Kantonsspital St. Gallen, St. Gallen, Switzerland); F. Mongelli, M. Bernasconi, M. Di Giuseppe, D. Christoforidis*, D. La Regina, M. Arigoni (Ente Ospedaliero Cantonale, Bellinzona, Switzerland); M. Adamina*, G. Peros, L. Guglielmetti, F. Solimene, M. Giardini, T. Bächler, A. S. Crugnale (Kantonsspital Winterthur, Winterthur, Switzerland); C. A. Gutschow*, M. Turina (Universitätsspital Zürich, Zürich, Switzerland); O. Ersen (Ankara University Medical School, Ankara, Turkey); M. A. Onan*, R. Kozan (Gazi University Medical Faculty Hospital, Ankara, Turkey); T. Erol*, H. A. Dincer (Hacettepe University Hospital, Ankara, Turkey); A. Yildiz* (Yildirim Beyazit University Yenimahalle Training and Research Hospital, Ankara, Turkey); N. Iflazoglu, O. Yalkın (Bursa City Hospital, Bursa, Turkey); A. Isik* (Erzincan University Hospital, Erzincan, Turkey); V. Ozben*, E. Aytac, Z. Aliyeva, E. Akaydin, B. B. Ozmen, B. Baca (Acibadem Atakent Hospital, Istanbul, Turkey); Y. Altinel*, F. Calikoglu, M. Tokocin, N. A. Hacim, A. Akbas, S. Meric, T. Vartanoglu, H. Yigitbas, C. Ercetin, G. Ercan (Bagcilar Research and Training Hospital, Istanbul, Turkey); I. Ozgur*, M. Keskin (Istanbul University Faculty of Medicine, Istanbul, Turkey); K. T. Saracoglu*, B. Cimenoglu, R. Demirhan, A. Kale, T. Simsek, E. C. Gundogdu (Kartal Dr Lutfi Kirdar Training and Research Hospital, Istanbul, Turkey); A. Abbasov* (Liv Hospital Ulus, Ulus, Turkey); M. Tanal*, B. Citgez, E. Bozkurt, S. G. Yetkin, M. Mihmanli (University of Health Sciences Sisli Hamidiye Etfal Training and Research Hospital, Sisli, Turkey); A. Alhamed, S. Ergun*, A. N. Sanli, M. Velidedeoglu, M. F. OZcelik, S. S. Uludag*, A. K. Zengin, S. Cebi, F. Demirkiran, T. Bese, A. S. Acikgoz, B. Kayan, Y. Aykanat, D. Mutlu (Cerrahpasa Medical Faculty, Istanbul. Turkey); B. Göksoy* (Sehit Prof. Dr İlhan Varank Training and Research Hospital, Sancaktepe, Turkey); Y. Kara*, M. A. Bozkurt, A. Kocatas (Kanuni Sultan Suleyman Training and Research Hospital, Istanbul, Turkey); H. Öğücü, G. Uslu, C. Arican, C. Tugmen, C. Aydin, D. Yesilyurt, E. K. Avci, E. Kebapçı, G. Kilinc, İ. Sert, K. Tuncer, M. Akalin, M. Emiroglu, S. Demirli Atici*, S. Salimoğlu, T. Kaya*, Y. Kirmizi (University of Health Sciences Tepecik Training and Research Hospital, Istanbul, Turkey); O. C. Tatar*, E. Yüksel, S. A. Güler, A. Yildirim, N. Z. Utkan, K. Gözal, H. Köken, A. Yabas (Kocaeli University Teaching Hospital, Kocaeli, Turkey); E. Gonullu, F. Altintoprak, E. Akin, B. Kamburoglu, R. Capoglu, F. Kucuk, H. Demir, G. Cakmak, N. Firat, F. Celebi, B. Kocer, B. Mantoglu, Z. Bayhan, E. Dikicier (Sakarya University Faculty of Medicine, Sakarya, Turkey); E. Colak*, G. O. Kucuk (Samsun Training and Research Hospital, Sakarya, Turkey); E. Karaman*, A. Kolusab, O. Karaaslan (Van Yuzuncu Yil University, Medical Faculty, Van, Turkey); I. Majid, S. Alshryda* (Al Jalila Children's Speciality Hospital, Dubai, United Arab Emirates); F. Abbas*, F. M. A. Abbas (Dubai Hospital, Dubai, United Arab Emirates); D. Mohammed*, M. A. Tahlak* (Latifa Women and Children Hospital, Dubai, United Arab Emirates); A. Yammahi, A. A. Albaroudi, B. Elyafawi, A. Saber, H. Khansaheb, H. Alsaadi*, N. Alzarooni* (Rashid Hospital, Dubai, United Arab Emirates); M. Bekheit*, B. S. Kamera, M. Elhusseini, P. Sharma, A. Ahmeidat, G. Gradinariu, W. Cymes, A. Hannah, G. Mignot, S. Shaikh*, J. Agilinko (Aberdeen Royal Infirmary, Aberdeen, UK); D. Angelou, D. Neely*, A. McCanny, B. McAree (Antrim Area Hospital, Northern Health and Social Care Trust, Antrim, UK); A. J. Baldwin, R. West*, E. Gammeri, A. Catton, Marinos S. Kouris (Stoke Mandeville, Wycombe General, High Wycombe, UK); J. Pereca*, J. Singh (University Hospital Ayr, Ayr, UK); Z. Seymour*, R. Jones*, S. Leeson, R. Peevor, A. K. Lala, C. Houlden (Ysbyty Gwynedd, Bangor, UK); J. Kahiu*, N. Hossain, S. Hosny (Barnet General Hospital, Barnet, UK); P. Patel*, S. Handa, M. Kaushal, A. Kler, V. Reghuram, S. Tezas (Furness General Hospital, Barrow-in-Furness, UK); K. Fairhurst*, C. Yates, S. Mitchell, J. Bunni, S. Richards, R. George, S. M. Lee, J. Phull, J. Frost*, S. Burnard, R. Crowley, A. Airey (Royal United Hospitals Bath, Bath,UK); K. Bevan*, R. Makin-Taylor, C. S. Ong, R. Callan, O. Bloom (Bedford Hospital, Bedford, UK); F. Aljanadi, N. Moawad, M. Jones*, A. Gregg, R. Jeganathan* (Royal Victoria Hospital, Belfast, UK); M. Pachl*, B. Martin (Birmingham Children's Hospital, Birmingham, UK); J. E. Archer*, A. Odeh, N. Siddaiah (Royal Orthopaedic Hospital, Bristol, UK); R. Singhal*, D. N. Naumann, S. Karandikar*, A. Syed*, O. N. Tucker, R. Alam, M. Kalkat* (Heartlands Hospital, Birmingham, UK); J. K. C. Mak, R. Kulkarni, N. Sharma, P. Nankivell, F. Tirotta, A. Parente, O. Breik, A. Kisiel, L. D. Cato, S. Saeed, A. Bhangu*, E. Griffiths*, A. M. Pathanki, S. Ford*, A. Desai*, M. Almond*, M. Kamal (Queen Elizabeth Hospital, Birmingham, UK); S. Sundar*, E. Y. L. Leung*, R. Kaur, C. Brett-Miller, F. E. Buruiana (Pan-Birmingham Gynaecological Cancer Centre, Birmingham, UK); G. Markose, A. De Gea Rico, A. Taib*, D. Myatt, A. Sulaiman Khaled, F. Younis* (East Lancashire Hospitals NHS Trust, Blackburn, UK); A. Sultana*, M. Taggarsi, L. Vitone*, J. Lambert, O. P. Vaz, I. Sarantitis, D. Shrestha, S. Timbrell, A. Shugaba (Royal Blackburn Hospital, Blackburn, UK); B. Quddus, J. Law, M. N. Bittar*, M. Creanga, M. Elniel, M. Youssef*, S. Ali, S. T. Qadri (Blackpool Victoria Hospital, Blackpool, UK); G. Brixton, L. Findlay, T. Klatte, A. Majkowska, J. Manson*, R. Potter (Royal Bournemouth Hospital, Bournemouth, UK); V. Oktseloglou*, F. Mosley*, M. F. I. De La Cruz Monroy, P. Bobak, I. Omar, S. Ahad, F. Langlands, V. Brown, M. Hashem (Bradford Royal Infirmary, Bradford, UK); L. Kennedy*, S. Jaunoo, E. Coomber, O. Williams (Royal Sussex County Hospital, Brighton, UK); M. Shalaby*, H. L. Rhodes (Bristol Royal Hospital for Children, Bristol, UK); A. Williams, A. Ridgway, D. Pournaras, E. Britton, E. Lostis, G. K. Ambler, H. Chu, J. Hopkins*, J. Manara, M. Chan, M. Doe, R. D. C. Moon, S. Lawday, T. Jichi, W. Singleton (Southmead Hospital, Bristol, UK); B. Main, T. Maccabe, C. Newton*, N. S. Blencowe, D. P. Fudulu, D. Bhojwani, M. Baquedano, M. Caputo*, F. Rapetto, O. Flannery, A. Hassan (University Hospitals Bristol and Weston NHS Foundation Trust, Bristol, UK); A. Coonar*, G. Aresu, C. Smith, D. Gearon, J. Hogan, I. S. Pradeep, H. Durio Yates, A. Peryt, Z. M. Barrett-Brown, M. King, N. Ahmadi, D. Jenkins*, N. Moorjani, F. Taghavi, F. Wells (Royal Papworth Hospital, Cambridge, UK); J. Hardie, S. Page, F. Anazor, S. D. King, J. Luck, S. Kazzaz* (Frimley Park, Frimley Health NHS FT, Camberley, UK); R. Mannion, G. D. Stewart*, J. Ramzi, M. Mohan, A. A. Singh, J. Ashcroft, O. J. Baker, P. Coughlin*, R. J. Davies*, A. Z. E. D. Durst, A. Abood, A. Habeeb, V. E. Hudson, A. Kolias, B. Lamb, L. Luke, S. Mitrasinovic, S. Murphy, A. W. T. Ngu, J. R. O'Neill*, S. Waseem, K. Wong, F. Georgiades, P. J. Hutchinson*, X. S. Tan, J. Pushpa-rajah, A. Colquhoun, L. Masterson, I. Abu-Nayla, C. Walker, A. Balakrishnan*, S. Rooney, E. Irune, M. H. V. Byrne, A. Durrani (Addenbrooke's Hospital, Cambridge, UK); A. Simoes, B. Eddy, E. Streeter, I. Ahmed, M. Yao*, W. Wang, A. Djouani, J. Tait-Bailey, M. Thomas, F. Hassan, S. Kommu (Kent and Canterbury Hospital, Canterbury, UK); S. Chopra (University Hospital Llandough, Penarth, UK); T. Richards*, A. Sethuraman Venkatesan, T. Combellack*, J. Williams, G. Tahhan, M. Mohammed, M. Kornaszewska, V. Valtzoglou, I. Deglurkar*, M. Rahman, U. Von Oppell, D. Mehta, M. Koutentakis, S. A. H. Syed Nong Chek, G. Hill, C. Morris*, M. Shinkwin, J. Torkington, J. Cornish (University Hospital of Wales, Cardiff, UK); R. Houston, S. Mannan*, F. Ayeni, H. Tustin, M. Bordenave, A. Robson (Cumberland Infirmary, Carlisle, UK); G. Dovell, R. Preece, P. Rolland* (Cheltenham General Hospital, Cheltenham, UK); B. H. Miranda* (St Andrew’s Centre for Plastic Surgery and Burns, Chelmsford, UK); A. Sobti, A. Khaleel*, A. Unnithan, K. Memon, R. R. Pala Bhaskar, F. Maqboul, F. Kamel, A. Al-Samaraee, R. Madani*, L. Kumar, P. Nisar, S. Agrawal* (Ashford and St Peter's Hospital, Chertsey, UK); D. Vimalachandran*, N. Manu, N. Eardley, E. Krishnan, O. L. Serevina, E. Martin, C. Smith, A. Jones, S. Roy Mahapatra, R. Clifford (Countess of Chester Hospital, Chester, UK); G. P. Jones, A. Gardner, S. S. Tripathi*, M. S. Greenhalgh (Lancashire Teaching Hospitals NHS Foundation Trust, Preston, UK); W. Matthews, K. Mohankumar, I. Khawaja, A. Palepa, T. Doulias (Colchester University Hospital, Colchester, UK); C. Gill, N. Dunne, D. R. Sarma, C. Godbole, W. Carlos, N. Tewari*, D. Jeevan, P. Naredla, A. Khajuria, H. Connolly, S. Robertson, C. Sweeney, G. Di Taranto, S. Shanbhag, K. Dickson, K. McEvoy, J. Skillman, M. Sait, H. Al-Omishy, M. Baig, B. Heer (University Hospitals Coventry and Warwickshire NHS Trusts, Coventry, UK); A. Brown* (Darlington Memorial Hospital, Darlington, UK); A. Ebrahim, A. Alwadiya, A. Goyal*, A. Phillips, A. Bhalla*, C. Demetriou, E. Grimley, E. Theophilidou, E. Ogden, F. L. Malcolm, G. Davies-Jones, J. C. K. Ng, M. Mirza, M. Hassan, N. Elmaleh, P. Daliya, S. Williams, A. Bateman*, Z. Chia (Royal Derby Hospital, Derby, UK); Y. Premakumar, Y. Jauhari, Z. Koshnow, D. Bowen, A. Uberai, F. Hirri, B. M. Stubbs* (Dorset County Hospital, Dorchester, UK); R. Crichton*, J. Sonksen, K. Aldridge (Russell's Hall Hospital, Dudley, UK); C. McDonald*, J. Manickavasagam*, K. Ragupathy*, S. Davison, S. Dalgleish*, N. McGrath, R. Kanitkar, C. J. Payne, L. Ramsay (Ninewells Hospital, Dundee, UK); C. E. Ng*, T. Collier, K. Khan*, R. Evans (University Hospital North Durham, Durham, UK); C. Brennan, D. E. Henshall, T. Drake*, E. M. Harrison*, V. Zamvar, A. Tambyraja, R. J. E. Skipworth*, G. Linder, R. McGregor, P. Brennan, J. Mayes, L. Ross, S. Smith, T. White, A. A. B. Jamjoom, R. Pasricha (Royal Infirmary of Edinburgh, Edinburgh, UK); K. Gallagher, R. Swan, H. Paterson*, Y. Maeda, A. M. F. Kwok, A. Tsiaousidou, P. G. Vaughan-Shaw, C. Boyle, D. Fernando, D. Tham, S. Leung, A. Laird* (Western General Hospital, Edinburgh, UK); T. Holme, S. Abbott, A. Razik, S. Thrumurthy, J. Steinke, M. Baker*, D. Howden, Z. Baxter, L. Osagie, M. Bence (Epsom & St Helier University Hospitals NHS Trust, Epsom, UK); G. E. Fowler, L. Massey, N. Rajaretnam, J. Evans, J. John, A. Goubran, N. Campain, F. D. McDermott*, J. S. McGrath*, M. Ng, J. Pascoe, J. R. A. Phillips, I. R. Daniels (Royal Devon and Exeter Hospital, Exeter, UK); J. A'Court, A. Konarski, G. Faulkner* (Royal Bolton Hospital, Bolton, UK); H. Emerson, K. Vejsbjerg, L. Pearce, W. McCormick, A. Fisher*, K. Singisetti, Y. Aawsaj, C. Barry (Gateshead Health NHS Foundation Trust, Gateshead, UK); O. Bajomo, S. Rizvi, C. Grimes*, K. Dusu, P. Y. Tint (Medway Hospital, Gillingham, UK); A. Kirk*, V. Irvine (Golden Jubilee National Hospital, Glasgow, UK); S. Lammy*, R. O'Kane (Royal Hospital for Sick Children, Glasgow, UK); L. Elliott, G. McCabe, D. Holroyd*, N. B. Jamieson (Glasgow Royal Infirmary, Glasgow, UK); A. Geddes, J. McMahon, J. McCaul*, M. Al-Azzawi, E. Aitken*, P. Glen*, L. O. H. Sinan, S. Lammy*, A. Grivas, E. J. Tilling (Queen Elizabeth University Hospital, Glasgow, UK); O. Brown, M. Boal, H. Dean, S. Higgs*, S. Stanger, H. Abdalaziz, J. Constable, H. Ishii, R. Preece, G. Dovell, R. Gopi Reddy (Gloucestershire Royal Hospital, Gloucester, UK); T. K. Madhuri*, A. Tailor, M. Flavin, D. Walker*, S. Humphries, H. Assalaarachchi (Royal Surrey NHS Foundation Trust, Guildford, UK); T. Curl-Roper, E. Westwood, C. Delimpalta, C. C. L. Liao, V. Velchuru* (James Paget University NHS Foundation Trust Hospital, Great Yarmouth, UK); D. A. Raptis, J. M. Pollok*, N. Machairas, B. Davidson, G. Fusai, F. Soggiu, S. Xyda, C. Hidalgo Salinas, H. Tzerbinis, T. Pissanou, J. Gilliland, S. Chowdhury, M. Varcada*, C. Hart, R. Mirnezami, J. Knowles, N. Angamuthu (Royal Free Hospital, London, UK); V. Vijay*, T. Shakir, R. Hasan, R. Tansey (Princess Alexandra Hospital, Harlow, UK); C. Hardie, E. Powell-Smith* (Harrogate District Hospital, Harrogate, UK); F. Kashora*, M. H. Siddique, A. Singh, C. Barmpagianni, A. Basgaran, A. Basha, V. Okechukwu, A. Bartsch, P. Gallagher, A. Maqsood, K. Sahnan, C. A. Leo, S. E. Lewis, H. K. Ubhi, R. Exley, U. Khan, P. Shah, S. Saxena, N. Zafar, H. Abdul-Jabar (Northwick Park Hospital, Harrow, UK); M. Patel, A. Shabana, A. Alanbuki*, O. Usman (Princess Royal Hospital, Haywards Heath, UK); C. T. Ong, W. Butterworth*, O. Budha Magar, M. El Hadi, S. Abas, J. Annett (Hereford County Hospital, Hereford, UK); E. Ross, M. Loubani*, A. Wilkins, H. Cao, H. Capitelli-McMahon, L. Hitchman, H. Ikram, A. Andronic, A. Aboelkassem Ibrahim, J. Totty (Hull University Teaching Hospitals NHS Trust, Hull, UK); J. Blanco*, R. Vanker, M. Ghobrial, G. Jones, S. Kanthasamy, H. Fawi, M. Awadallah, F. Chen, J. Cheung* (Hinchingbrooke Hospital, Huntingdon, UK); A. Moscalu*, T. Bhuvanakrishna, L. Bibby, M. Sinclair (Ipswich Hospital, Ipswich, UK); M. A. K. Nahid*, L. Williams, P. S. Basnyat, A. K. Shrestha* (William Harvey Hospital, Ashford, UK); N. K. Kumaran, S. Sambhwani, N. A. Sheikh, O. M. Taylor* (Kettering General Hospital, Kettering, UK); I. Liew*, A. Al-Sukaini, S. Mediratta, D. Saxena (Queen Elizabeth Hospital, King's Lynn, UK); A. Sgrò, M. M. Rashid, K. Milne, J. McIntyre, M. A. Akhtar*, A. Turnbull, A. Brunt, K. E. Stewart (Victoria Hospital, Kirkcaldy, UK); M. S. J. Wilson*, D. Rutherford, K. McGivern, E. Massie (Forth Valley Royal Hospital, Larbert, UK); M. Ho*, R. G. Wade*, J. Johnstone, G. Bourke, A. Brunelli, H. Elkadi, M. Otify*, C. Pompili, J. R. Burke, E. Bagouri, M. Chowdhury, Z. Abual-Rub, A. Kaufmann, S. Munot, T. Lo*, A. Young, M. Kowal, J. Wall, A. Peckham-Cooper (The Leeds Teaching Hospitals NHS Trust, Leeds, UK); G. R. Layton*, B. Karki, H. Jeong, S. Pankhania, S. Asher, A. Folorunso, S. Mistry, B. Singh, J. Winyard, J. Mangwani (Leicester General Hospital, Leicester, UK); E. J. Caruana*, A. Mohammad, M. Acharya, K. Chandarana, K. Ang, M. F. Chowdhry, S. Rathinam, A. Nakas (Glenfield Hospital, Leicester, UK); A. Boddy*, T. Hossain, C. Ashmore, S. Annamalai, A. Kourdouli, E. Irvine, A. Al-Harbawi, K. Kassam*, A. Al-Harbawee, A. Miller, M. Mair (Leicester Royal Infirmary, Leicester, UK); R. Lunevicius*, A. R. G. Sheel, M. Sundhu, A. J. A. Santini, M. S. A. T. Fathelbab, K. M. A. Hussein, Q. M. Nunes, R. P. Jones, K. Shahzad, I. Haq, M. M. A. S. Baig, J. L. Hughes, A. Kattakayam, K. Rajput, N. Misra, S. B. Shah, A. L. Clynch, N. Georgopoulou, H. M. Sharples, A. A. Apampa, I. C. Nzenwa, A. Sud (Aintree Hospital, Liverpool, UK); A. Harky, B. H. Kirmani*, M. Shackcloth (Liverpool Heart and Chest Hospital, Liverpool, UK); M. D. Jenkinson*, R. Zakaria, T. Elmoslemany, C. P. Millward (The Walton Centre NHS Foundation Trust, Liverpool, UK); R. Baron*, D. Dunne, P. Szatmary, A. Thomas, F. McNicol, S. Gahunia, D. Sochorova, G. E. Nita, R. McKinney, J. Russ, J. R. Tan (Royal Liverpool University Hospital, Liverpool, UK); R. Harwood, H. J. Corbett (Alder Hey In The Park, Liverpool, UK); C. Rossborough, B. L. Skelly (Altnagelvin Area Hospital, Londonderry, UK); N. A. Che Bakri, S. Nazarian, R. Vashisht*, L. Jiao, Z. Jawad (BUPA Cromwell Hospital, London, UK); A. Y. Allan, C. Kontovounisios, T. Grove, O. Warren*, M. G. Fadel (Chelsea and Westminster Hospital, London, UK); M. Chatzikonstantinou, P. Sorelli*, S. Rahman, M. Hadjipavlou (Queen Elizabeth Hospital, Woolwich, UK); C. Holbrook, C. Chong, D. Kufeji (Evelina London Children's Hospital, London, UK); S. R. Rufai, I. C. Lloyd, G. James*, A. Chari, A. H. D. Silva (Great Ormond Street Hospital for Children, London, UK); L. Stroman, B. Challacombe*, A. Sayasneh*, M. Najdy, A. Billè, S. Fraser, P. Agoston, V. Rizzo, J. King, R. Nath, S. McCrindle , G. Mehra, K. Harrison-Phipps, J. Pilling, L. Okiror, T. Routledge, L. Mills, A. Wali, K. El-Boghdadly* (Guy's Hospital, Lopndon, UK); C. Fotopoulou*, S. Saso, M. Fehervari*, J. Ploski, S. Ghaem-Maghami, D. Spalding, P. Rajagopal, M. Pai, N. Habib, Z. Jawad, S. Hamrang-Yousefi, L. Jiao (Hammersmith Hospital, Lndon, UK); S. Tayeh*, T. Chase, L. Humphreys, J. Ayorinde, A. Ghanbari, T. Cuming (Homerton University Hospital, London, UK); N. Anscomb*, R. Baldwin-Smith, M. Rizk, C. Grainger, M. Davies, A. Surendran, J. W. Nunoo-Mensah (King's College Hospital, London, UK); M. Dunstan*, P. Beak, I. Gerogiannis* (Kingston Ηospital NHS Foundation Trust, London, UK); A. Jain, A. Menon, B. Pramodana, D. Choi*, H. J. Marcus, L. Webber, R. May, R. Hutchison, V. Luoma (The National Hospital for Neurology and Neurosurgery, London, UK); S. Ranjit, J. Parakh, V. Sarodaya, A. Daadipour, M. Khalifa (Newham University Hospital, London, UK); K. D. Bosch*, V. Bashkirova, L. S. Dvorkin, V. K. Kalidindi (North Middlesex University Hospital, London, UK); J. Dudek, T. Singhal*, S. El-Hasani (Princess Royal University Hospital, London, UK); A. De Souza, M. Cannoletta, M. Rochon, S. Bhudia (Royal Bromtpon Hospital, London, UK); S. Bennett, L. Navaratne, M. Venn, V. Yip*, B. Kayani, C. Sohrabi, H. M. Kocher, A. Minicozzi, A. Banerjee, T. Sullivan, R. Sivaprakasam*, A. Anzak, K. Ghufoor*, M. A. Thaha*, C. Knowles, F. S. Ledesma, P. Patki*, D. Popova, P. Sadigh*, R. Ramamoorthy, C. Uff*, L. Attwell, C. Tanabalan, M. A. Goh, J. D. Jayasinghe, I. Leal Silva, B. Thakur, M. Lebe, M. S. Thet, F. Hughes*, R. Rahman, O. Fuwa (Royal London Hospital, London, UK); J. Sanders*, A. Oo, T. Bueser, M. Curtis, S. A. Stamenkovic (Cardiac Unit, St Bartholomew's Hospital, London, UK); T. Abbott*, S. Anwar, C. Sohrabi (St Bartholomew's Hospital, London, UK); K. Williams*, E. Chung, R. Hagger, A. Karim, A. Hainsworth, M. Flatman, A. Trompeter, C. Hing, O. Brown, P. Tsinaslanidis, M. W. Benjamin, A. Leyte, C. Tan, J. Smelt, P. Vaughan, G. Santhirakumaran, I. Hunt, M. Raza, A. Labib (St George's Hospital, London, UK); X. Luo, A. Sudarsanam, A. Rolls, O. Lyons, S. Onida, J. Shalhoub, K. Sugand, C. Park, K. M. Sarraf*, S. Erridge, J. Kinross*, M. Denning, S. Yalamanchili, A. Abuown, M. Ibrahim, G. Martin (St Mary's Hospital, London, UK); D. Davenport, S. Wheatstone* (St Thomas' Hospital, London, UK); V. Kasivisvanathan*, K. Kapriniotis, A. Elhamshary, S. M. B. Imam (University College London Hospital At Westmoreland Street, London, UK); N. Kalavrezos*, D. Sinha, M. Chand, L. Green, N. Beech, R. McEwen, H. Kiconco (University College London Hospital, London, UK); S. M. Andreani*, M. F. Bath, A. Sahni, N. Judkins, L. Rigueros Springford, C. Sohrabi, J. Bacarese-Hamilton, F. G. Taylor, P. Patki*, C. Tanabalan (Whipps Cross University Hospital, London, UK); C. Parmar*, S. McCluney, S. Shah (The Whittington Hospital, London, UK); R. Talwar*, K. Patel, A. Askari, P. S. Jambulingam, S. Shaw, A. Maity, C. Hatzantonis, J. Sagar, S. Kudchadkar, N. Cirocchi, C. H. Chan (Luton and Dunstable University Hospital, Luton, UK); J. Reynolds, M. E. Alexander, C. J. Smart* (Macclesfield District General Hospital, Macclesfield, UK); B. Jayasankar, D. Balasubramaniam*, K. Abdelsaid, N. Mundkur, B. Gallagher* (Tunbridge Wells Hospital, Tunbridge Wells, UK); J. Shah*, J. Anthoney, O. Emmerson (North Manchester General Hospital, Manchester, UK); N. Stylianides*, M. Abdalla, K. Newton, K. Bhatia*, R. Edmondson, L. Abdeh, D. Jones, M. Zeiton, O. Ismail, H. Naseem, R. Advani (Manchester Royal Infirmary, Manchester, UK); S. Duff*, F. Moura, B.C. Brown, A. Khan, P. Asaad, B. Wadham, I. A. Aneke, J. Collis, H. Warburton (Wythenshawe Hospital, Manchester, UK); A. Fell*, A. Smith, C. Halkias, J. Evans, S. Nikolaou, C. English*, S. Kristinsson, T. Oni, N. Ilahi, K. Ballantyne, Z. Woodward, R. Merh (Queen Elizabeth The Queen Mother Hospital, Margate, UK); J. Dunning, Y. Viswanath, K. Freystaetter, J. Dixon*, J. N. Hadfield, A. Hilley, A. Egglestone, B. Smith (James Cook University Hospital, Middlesborough, UK); T. Hine, B. Keeler*, R. E. Soulsby, A. Taylor* (Milton Keynes University Hospital, Milton Keynes, UK); E. Davies*, O. Ryska, T. Raymond, S. Rogers*, A. Tong, P. Hawkin (Royal Lancaster Infirmary, Lancaster UK); S. Tingle, F. Abbadessa, A. Sachdeva, B. Rai*, C. D. Chan, I. McPherson, K. Booth, F. Mahmoud Ali, S. Pandanaboyana, T. Grainger, S. Nandhra, A. Patience, A. Rogers*, C. Roy, T. Williams, N. Dawe, C. McCaffer, J. Riches, S. Bhattacharya, J. Moir, N. S. Kalson, H. Elamin Ahmed, C. Mellor, C. Saleh, R. M. Koshy, J. Hammond*, L. Sanderson, S. Wahed, A. W. Phillips, K. Ghosh* (Newcastle Upon Tyne Hospitals NHS Foundation Trust, Newcastle Upon Tyne UK); A. Tang, A. J. Beamish, C. Price, D. Bosanquet*, D. Magowan, F. Solari, G. Williams, H. Nassa, L. Smith (Royal Gwent Hospital, Newport, UK); B. Robertson-Smith, A. Mahmoud, P. Ameerally, J. G. Finch*, C. Gnanachandran, I. Pop, M. Rogers, Y. Yousef, I. Mohamed, R. Woods, H. Zahid, G. Mundy* (Northampton General Hospital, Northampton, UK); M. Youssef*, L. Sreedharan, D. Baskaran, I. Shaikh, K. Seebah, J. Reid, D. Watts, V. Kouritas, D. Chrastek, G. Maryan, D. F. Gill, F. Khatun (Norfolk and Norwich University Hospital, Norwich, UK); K. Gajjar, K. Williamson, D. Bratt, K. Konstantinidi, T. Walton*, N. Burnside*, H. Weaver, M. Hawari, E. Addae-Boateng, R. A. Rollett*, M. L. Collins, M. S. Tamimy, H. Riyat, J. Wen, J. Neil-Dwyer (Nottingham City Hospital, Nottingham, UK); H. Brewer, D. Humes*, D. Worku, A. Chowdhury*, O. Oyende, C. Lewis-Lloyd, A. Adiamah, A. Koh, J. Jackman, R. Vohra*, A. Navarro*, J. Reilly (Queens Medical Centre, Nottingham, UK); A. Aujayeb, D. Townshend*, N. McLarty, A. Shenfine, K. Jackson, C. Johnson (Northumbria NHS Hospital Trust, UK); D. Dass, D. Ford (Robert Jones and Agnes Hunt Orthopaedic Hospital, Oswestry, UK); S. C. Winter*, E. Belcher*, D. Stavroulias, F. Di Chiara, K. Wallwork, A. Qureishi, M. Lami, S. Sravanam, S. Mastoridis, K. Shah, S. Chidambaram, R. Smillie, A. V. Shaw, S. Bandyopadhyay, C. Cernei, C. Bretherton, D. Jeyaretna*, M. Ganau*, R. J. Piper*, E. Duck, S. Brown, C. Jelley, S. C. Tucker*, G. Bond-Smith*, X. L. Griffin*, G. D. Tebala, N. Neal*, M. Vatish*, T. M. Noton, H. Ghattaura, M. Maher, H. Fu, O. B. F. Risk, H. Soleymani Majd, S. Sinha*, S. Shankar, A. Aggarwal, H. Kharkar, K. Lakhoo*, C. Verberne, S. Mastoridis (Oxford University NHS Foundation Trust, Oxford, UK); B. Dean*, C. Luney, R. Myatt, M. A. Williams, J. McVeigh (Nuffield Orthopaedic Centre, Oxford, UK); L. J. Rogers*, P. L. Labib, D. Miller, G. Minto, N. Hope, A. Marchbank, K. Emslie, P. Panahi, B. Ho, C. Perkins, E. Clough, H. Roy, I. Enemosah, R. Campbell, J. Natale, K. Gohil, M. Rela, N. Raza, (Derriford Hospital, Plymouth, UK); I. Biliatis* (Poole Hospital, Poole, UK); J. Khan*, G. Thiruchandran, S. K. C. Toh*, Y. Ahmad*, A. Allana, C. Bellis, O. Babawale, Y. C. Phan, U. Lokman, M. Ismail, T. Koc, A. Witek, L. Duggleby, S. Shamoon, S. Stefan, H. Clancy (Queen Alexandra Hospital, Portsmouth, UK); R. Chadha*, S. B. Middleton, K. Wilmott, C. Hayden, C. Mclaren, J. Sutton, A. Whyte (Royal Berkshire Hospital, Reading, UK); A. Belgaumkar*, A. Day, C. Gilbert, B. Oyewole, P. Narayan, H. Dent, A. Sandhya, T. De Silva, S. Waheed (Surrey and Sussex Healthcare NHS Trust, Sussex, UK); A. Day, K. Kapoor, A. P. Belgaumkar*, P. Narayan, M. Fahim (East Surrey Hospital, Redhill, UK); T. Gala, R. Mithany, R. Morgan*, M. Abdelkarim, S. Ibrahim, A. Maw*, A. Asqalan, G. Sundaram Venkatesan (Glan Clwyd Hospital, Rhyl, UK); S. Singh, S. Mukherjee*, D. Ferguson, C. Smith, A. Mansuri, A. Thakrar, L. Wickramarachchi, R. Cuthbert, S. Sivayoganathan, K. Chui, E. Karam, C. Dott, S. Shankar (Queen's Hospital, Romford, UK); K. Madhvani, M. Hampton, A. P. Hormis* (Rotherham District General Hospital, Rotherham, UK); M. Thomas, L. Pearce*, D. M. Fountain, R. Laurente, K. V. Sigamoney, M. Dasa, K. George*, Z. Naqui*, M. Galhoum, C. Lipede, A. Gabr, A. Radhakrishnan, M. T. Hasan, R. Kalenderov, O. Pathmanaban, F. Colombo, R. Chelva (Salford Royal NHS Foundation Trust, Salford, UK); G. Branagan*, L. Longstaff, D. Ding, C. Barlow, J. Foster (Salisbury NHS Foundation Trust, Salisbury, UK); J. Edwards*, A. Ward, D. Tadross, L. Majkowski, C. Blundell, S. Forlani, R. Nair, S. Guha, S. R. Brown, C. Steele, C. J. Kelty*, T. Newman, M. Lee, G. Chetty*, G. Lye, S. P. Balasubramanian, N. Sureshkumar Shah, M. Sherif, A. Al-mukhtar, E. Whitehall, A. Giblin, F. Wells, A. Sharkey, A. Adamec, S. Madan (Sheffield Teaching Hospital NHS Foundation Trust, Sheffield, UK); B. Narice, M. Sterrenburg, A. Thompson, I. Varley, M. Stavrakas, O. Rominiyi, J. Ray, A. Adamec, M. Crank, A. Bacon, Y. Al-Tamimi, J. Catto, S. Saad, N. N. Abd Kahar, S. Sinha (Royal Hallamshire Hospital, Sheffield, UK); A. Sou, D. Simpson, E. Hamilton*, J. Blair (Shrewsbury and Telford Hospitals, Shrewsbury, UK); S. Jallad, J. Lord, C. Anderson, J. El Kafsi*, K. Logishetty*, A. Saadya, R. Midha, M. Ip, H. Subbiah Ponniah, T. Stockdale, T. Bacarese-Hamilton, L. Foster, A. James, N. Anjarwalla, D. Marujo Henriques, R. Hettige, C. Baban, A. Tenovici, G. Salerno (Wexham Park, Frimley Health NHS FT, UK); R. Singh*, J. Lane, H. V. Colvin, A. Badran*, A. Cadersa, S. Williams, A. Cumpstey, Z. Hamady, R. Aftab, F. Wensley, J. Byrne, V. Morrison-Jones, G. K. Sekhon, H. Shields, Z. Shakoor, A. Yener, T. Talbot, A. Khan, A. Alzetani*, R. Cresner (Southampton General Hospital, Southampton, UK); B. H. B. Babu*, A. S. D. Liyanage, S. Newman, I. Blake, C. Weerasinghe (Southport and Formby District General Hospital, Southport, UK); R. Baumber, J. Parry* (Royal National Orthopaedic Hospital, Stanmore, UK); C. Menakaya*, J. I. Webb, M. Antar, N. Modi, R. Sofat, J. Noel, R. Nunn, S. Adegbola, F. Eriberto, V. Sharma, R. Tanna, S. Lodhia (East and North Hertfordshire NHS Trust, Lister Hospital, Stevenage, UK); D. Johnson*, I. Hughes, J. Hall, J. Rooney, S. Chatterji, Y. Zhang, R. Owen, M. Rudic, J. Hunt (Stepping Hill Hospital, Stockport, UK); D. Zakai, M. Thomas*, A. Aladeojebi, M. Ali, A. Gaunt*, B. Barmayehvar, M. Kitchen, M. Gowda, F. Mansour, M. Jarvis, E. Halliday, R. Lefroy, P. Nanjaiah, S. Ali, M. Kitchen* (Royal Stoke University Hospital, Stoke, UK); D. J. Lin, A. D. Rajgor, R. J. Scurrah, C. Kang, L. J. Watson, G. Harris, T. Royle*, Y. Cunningham, G. James, B. Steel, A. C. O. Luk (Sunderland Royal Hospital, Sunderland, UK); A. J. Boulton, M. T. Khan, G. Bakolas, I. Ahmed* (Good Hope Hospital, Sutton Coldfield, UK); P. Herrod, E. Gemmill*, H. Boyd-Carson, M Jibreel, E. Lenzi, T. Saafan, D. Sapre (King's Mill Hospital, Sutton-in-Ashfield, UK); Z. Li*, K. Parkins, N. Spencer, R. Harries, R. J. Egan, D. Motter, C. Jenvey, R. Mahoney, N. Fine, T. Minto, A. Henry (Morriston Hospital, Swansea, UK); M. Hollyman*, C. Grieco, C. Gemmell (Musgrove Park Hospital, Taunton, UK); H. Whitmore, M. S. Babar, S. Goodrum, R. Scott, B. Collard, K. Lau, E. Thomas, A. Patel, J. Allison, J. Bowen (Torbay and South Devon NHS Trust, Torquay, UK); A. Dias, B. Mahendran, S. Gopalswamy* (Royal Cornwall Hospital, Truro, UK); S. Patil, L. Scott, J. Sarveswaran*, M. Michel, S. Ravindran (Pinderfields Hospital, Wakefield, UK); K. Subba, A. K. Abou-Foul, M. Khalefa, F. Hossain*, T. Moores, L. Pickering (Walsall Manor Hospital NHS Trust, Walsall, UK); G. Stables*, A. Doorgakant*, V. G. Thiruvasagam, J. Carter, S. Reid, R. Mohammed, W. Marlow (Warrington & Halton Teaching Hospitals NHS Trust, Warrington, UK); H. Ferguson*, R. Wilkin, C. Konstantinou, D. Yershov, J. Vatish, A. Denning (South Warwickshire NHS Foundation Trust, UK); H. B. Shah, G. W. V. Cross, P. Seyed-Safi*, Y. W. Smart, A. Kuc, M. Al-Yaseen (Watford General Hospital, Watford, UK); J. Olivier*, M. Hanna, P. Eskander, R. Duncan, S. Halaseh (Weston General Hospital, Weston-super-Mare, UK); R. Das* (Hampshire Hospitals NHS Trust, Basingstoke, UK); H. Wynn Jones, H. Divecha*, C. Whelton, T. Board (Wrightington, Wigan & Leigh NHS Foundation Trust, Wigan, UK); S. Powell*, C. Magee*, K. Agarwal*, E. Mangos, T. Nambirajan (Wirral University Teaching Hospital, Birkenhead, UK); R. Vidya*, G. Chauhan, J. Kaur, A. Burahee, S. Bleibleh, N. Pigadas*, D. Snee, S. Bhasin*, A. Crichton, A. Habeebullah, A. S. Bodla, N. Yassin*, M. Mondragon, V. Dewan (Royal Wolverhampton NHS Trust, Wolverhampton, UK); I. Flindall, V. Mahendran, A. Hanson (Worcestershire Royal Hospital, Worcester, UK); E. Jenner, J. Richards, K. Thomas-Fernandez, R. Wall (Worcestershire Acute Hospitals NHS Trust, Worcester, UK); A. Alqallaf, A. Ben-Sassi*, I. Mohamed, K. Mellor, P. Joshi, Y. Joshi (Wrexham Maelor Hospital, Wrexham, UK); R. Young*, V. Miu, K. Sheridan, L. MacDonald, S. Green, L. Onos (York Teaching Hospitals NHS Trust, York, UK); J. J. Wong, L. Napolitano*, M. Hemmila (Michigan Medicine, Ann Arbor, USA); D. Amin*, S. Abramowicz, S. M. Roser (Emory University, Atlanta, USA); K. A. Olson, C. Riley, C. Heron, T. Cardenas*, E. Leede, M. Thornhill, A. B. Haynes*, K. McElhinney, S. Roward, M. D. Trust*, C. E. Hill, P. G. Teixeira* (Dell Seton Medical Center at the University of Texas at Austin, Austin, USA); E. Etchill, K. Stevens*, M. R. Ladd, C. Long, J. Rose, A. Kent, P. Yesantharao, D. Vervoort, H. Jenny, A. Gabre-Kidan, A. Margalit, L. Tsai, H. Malapati, L. Yesantharao (Johns Hopkins Hospital, Baltimore, USA); H. Abdou, J. Diaz*, M. Richmond, J. Clark, L. O'Meara, N. Hanna (University of Maryland Medical Center, Baltimore, USA); Y. Ying*, J. Fleming, A. Ovaitt, J. Gigliotti, A. Fuson (University of Alabama, Birmingham, USA); Z. Cooper*, A. Salim*, S. A. Hirji, A. Brown, C. Chung, L. Hansen, B. U. Okafor, V. Roxo, C. P. Raut, J. S. Jolissaint, D. A. Mahvi (Brigham and Women's Hospital, Boston, USA); H. Kaafarani*, K. Breen, B. Bankhead-Kendall, O. Alser, H. Mashbari, G. Velmahos, L. R. Maurer, M. El Moheb, A. Gaitanidis, L. Naar, M. A. Christensen, C. Kapoen, K. Langeveld, M. El Hechi, A. Mokhtari (Massachusetts General Hospital, Boston, USA); M. H. Haqqani, F. T. Drake* (Boston Medical Center, Boston, USA); A. Goldenberg-Sandau*, B. Galbreath (Cooper University Hospital, Camden, USA); C. Reinke*, S. Ross, K. Thompson, D. Manning, R. Perkins (Atrium Health Carolinas Medical Center, Charlotte, USA); E. Eriksson*, H. Evans (Medical University of South Carolina, Charleston, USA); M. Masrur, P. Giulianotti, E. Benedetti (University of Illinois at Chicago, Chicago, USA); G. Chang*, J. Ourieff, D. Dehart (Mount Sinai Hospital, USA); A. Dorafshar, T. Price, A. R. Bhama, A. Torquati*, E. Cherullo, R. Kennedy, J. Myers (Rush University Medical Center, Chicago, USA); K. Rubin* (University Hospitals of Cleveland, Cleveland, USA); V. S. Ban*, S. G. Aoun, H. H. Batjer, J. Caruso (University of Texas Southwestern, Dallas, USA); H. Carmichael, C. G. Velopulos*, F. L. Wright, S. Urban, R. C. McIntyre Jr, T. J. Schroeppel*, E. A. Hennessy, J. Dunn*, L. Zier (University of Colorado Hospital/Memorial Hospital/Medical Center of the Rockies, Loveland, USA); C. Burlew*, J. Coleman* (Denver Health, Denver, USA); K. P. Colling* (Saint Mary's Medical Center-Essentia Health, Duluth, USA); B. Hall, H. E. Rice*, E. S. Hwang, S. A. Olson, D. Moris (Duke University Medical Center, Durham, USA); R. Verma*, R. Hassan (Nassau University Medical Center, Nassau, USA); A. Volpe, S. Merola (NewYork Presbyterian Queens, Flushing, USA); L. A. O'Banion*, J. Lilienstein, R. Dirks (University of California San Francisco-Fresno, Fresno, USA); H. Marwan*, M. Almasri*, G. Kulkarni, M. Mehdi, A. Abouassi, M. Abdallah, M. San Andrés, J. Eid, E. Aigbivbalu, J. Sundaresan, B. George (University of Texas Medical Branch, Galveston, USA); A. Ssentongo, P. Ssentongo, J. S. Oh, J. Hazelton*, J. Maines, N. Gusani, M. Garner, S. Horvath (Pennsylvania State University, State College, USA); F. Zheng* (Houston Methodist Hospital, Houston, USA); M. Ujiki (Northshore University Healthsystem, Chicago, USA); G. Kinnaman, A. Meagher*, I. Sharma, E. Holler (Iu Health Methodist Hospital, Indianapolis, USA); K. McKenzie*, J. Chan, K. Fretwell, W. Nugent III, A. Khalil, D. Chen, N. Post, T. Rostkowski, D. Brahmbhatt (Jamaica Hospital, Richmond Hill, USA); K. Huynh, M. L. Hibbard (Kaiser Permanente West Los Angeles, Los Angeles, USA); M. Schellenberg* (LAC+USC Medical Center, Los Angeles, USA); R. C. G. Martin*, N. Bhutiani (University of Louisville Hospital and Norton Hospital, Louisville, USA); E. Giorgakis*, J. Laryea, A. Bhavaraju, K. Sexton, M. Roberts, M. Kost, M. Kimbrough, L. Burdine, K. Kalkwarf, R. Robertson (University of Arkansas for Medical Sciences, Little Rock, USA); A. Gosain*, L. Camp, R. Lewit (Le Bonheur Children's Hospital, Memphis, USA); J. P. Kronenfeld, E. Urrechaga, N. Goel, R. Rattan, V. Hart*, M. Allen, G. Gilna (Jackson Memorial Hospital, Miami, USA); A. Cioci, G. Ruiz*, M. Allen, K. Rakoczy, W. Pavlis, R. Saberi (University of Miami Hospital, Miami, USA); R. Morris*, B. S. Karam (Froedtert Hospital, Milwaukee, USA); C. E. M. Brathwaite*, H. Liu, P. Petrone, H. Hakmi, A. H. Sohail, G. Baltazar, R. Heckburn (NYU Langone Health-NYU Winthrop Hospital, Mineola, USA); R. M. Nygaard*, E. T. Colonna, F. W. Endorf, M. J. Hill (Hennepin Healthcare, Minneapolis, USA); A. Maiga, B. Dennis*, J. H. Levin, M. Lallemand (Vanderbilt University Medical Center, Nashville, USA); R. Choron, G. Peck*, F. Soliman, S. Rehman (Robert Wood Johnson University Hospital, New Brunswick, USA); N. Glass*, B. Juthani, D. Deisher (The University Hospital, Newark, USA); N. M. Ruzgar, S. J. Ullrich*, M. Sion* (Yale New Haven Hospital, New Haven, USA); C. Paranjape*, M. El Moheb, A. R. Kar (Newton Wellesley Hospital, Newton, USA); C. Gillezeau, J. Rapp, E. Taioli, B. A. Miles*, N. Alpert (Mount Sinai Hospital, USA); D. Podolsky*, N. L. Coleman, M. P. Callahan (NewYork-Presbyterian/Columbia University Medical Center, New York, USA); I. Ganly*, L. Brown (Memorial Sloan Kettering Cancer Center, New York, USA); J. R. T. Monson (AdventHealth Orlando, Orlando, USA); A. Dehal* (Kaiser Permanente Panorama City Medical Center, Panorama City, USA); A. Abbas, A. Soliman, B. Kim, C. Jones, E. Dauer, E. Renza-Stingone, E. Hernandez, E. Gokcen, E. Kropf, H. Sufrin, H. Hirsch, H. Ross, J. Engel, J. Sewards, J. Diaz, J. Poggio, K. Sanserino, L. Rae, M. Philp*, M. Metro, P. McNelis, R. Petrov, S. Rehman, T. Pazionis (Temple University Hospital, Philadelphia, USA); B. Till, R. Lamm, A. J. Rios-Diaz, F. Palazzo* (Thomas Jefferson University Hospital, Philadelphia, USA); M. Rosengart*, K. Nicholson (University of Pittsburgh Medical Center, Pittsburgh, USA); M. M. Carrick*, K. Rodkey (Medical City Plano, Plano, USA); A. Suri, R. Callcut* (UC Davis Medical Center, Sacramento, USA); S. Nicholson*, N. Talathoti (UT Health San Antonio (UTHSA), University Hospital (UHS), San Antonio, USA); D. Klaristenfeld* (Kaiser Permanente San Diego Medical Center, USA); Biffl W*, Marsh C, Schaffer K (Scripps Memorial Hospital, San Diego, USA); A. E. Berndtson*, S. Averbach, T. Curry (University of California, San Diego, USA); R. Kwan-Feinberg*, E. Consorti, R. Gonzalez, R. Grolman, T. Liu, O. Merzlikin (Alta Bates Summit Medical Center (Sutter Health), Oakland, USA); M. K. Abel, D. Ozgediz, M. Boeck, L. Z. Kornblith*, B. Nunez-Garcia, B. Robinson, P. Park (University of California San Francisco (UCSF), San Francisco, USA); A. F. Utria, S. E. Rice-Townsend*, P. Javid, J. Hauptman, K. Kieran (Seattle Children's Hospital, Seattle, USA); D. Nehra*, A. Walters, J. Cuschieri, G. H. Davidson (Harborview Medical Center, Seattle, USA); J. Nunez*, R. Cosker (University of Utah Healthcare, Salt Lake City, USA); S. Eckhouse* (Washington University School of Medicine, St Louis, USA); A. Choudhry, W. Marx* (SUNY Upstate University Hospital, Syracuse, USA); T. Jamil*, S. Seegert, S. Al-Embideen (ProMedica Toledo Hospital, Toledo, USA); M. Quintana*, H. Jackson (The George Washington University Hospital, Washington, D.C., USA); S. D. Wexner, I. Kent (Cleveland Clinic Florida, Weston, USA); P. N. Martins* (University of Massachusetts, UMass Memorial Hospital, Worcester, USA); M. Alshehari*, H. Al-Naggar*, M. Alsayadi, M. Alyazidi, S. Shream, W. Alhaddad, A. Maqus, M. Abu Hamra'a, R. Alsayadi, R. Ghannam, S. Al-Maqtari, S. Masdoos, Y. Al-Harazi, H. Bajjah (Al-Thawra Modern General Hospital, Sana’a, Yemen); S. Al-Ameri, M. Aldawbali (Royal Hospital, Sana’a, Yemen); G. P. Gwini* (Mpilo Central Hospital, Bulawayo, Zimbabwe); D. Mazingi* (Parirenyatwa hospital, Harare, Zimbabwe).

## Funding

This report was funded by a National Institute for Health Research (NIHR) Global Health Research Unit Grant (NIHR 16.136.79), Association of Coloproctology of Great Britain and Ireland, Bowel & Cancer Research, Bowel Research UK, Association of Upper Gastrointestinal Surgeons, British Association of Surgical Oncology, British Gynaecological Cancer Society, European Society of Coloproctology, Medtronic, NIHR Academy, The Urology Foundation, Sarcoma UK, Vascular Society for Great Britain and Ireland, Yorkshire Cancer Research, and the MRC Health Data Research UK (HDRUK/CFC/01), an initiative funded by UK Research and Innovation, Department of Health and Social Care (England) and the devolved administrations, and leading medical research charities. L.B.M. and G.V.G. also acknowledge the Wellcome Trust 4-year studentship programme in mechanisms of inflammatory disease (MIDAS; 215182/Z/19/Z). The funders had no role in study design, data collection, analysis and interpretation, or writing of this report. The views expressed are those of the authors and not necessarily those of the National Health Service, the NIHR, or the UK Department of Health and Social Care.

## Supplementary Material

znab183_Supplementary_Data
