# Supplementary material for: Machine learning risk prediction of mortality for patients undergoing surgery with perioperative SARS-CoV-2: the COVIDSurg mortality score
Source: Br J Surg. 2021 Jul 6;108(11):1274–92. doi: 10.1093/bjs/znab183 (PMC8344569; doi:10.1093/bjs/znab183)
Supplement: znab183_Supplementary_Data [file znab183_supplementary_data.docx]

**Supplementary material:**

**Machine learning risk prediction of mortality for patients undergoing surgery with perioperative SARS-CoV-2: the COVIDSurg Mortality Score**

**Appendix 1 Methods**

**Appendix 2 Results**

**Appendix 3 Supplementary tables and figures**

**Appendix 1: Methods**

The study was registered as clinical audit in the United Kingdom (UK). In other countries, local or national approvals were sought through research ethics committees, institutional review board, or equivalent bodies, as appropriate.

*Candidate predictor variables for prognostic model*

We planned to include variables that are available globally, even in resource limited environments, without the need for additional tests. To enable this model to inform preoperative decision making, we only selected variables that are systematically available before surgery. To achieve this, 16 candidate predictors were selected *a priori* to be included and processed. These were selected from three domains:

- Patient: age, sex, haemoglobin (g/dL), white cell count (10^9/L), C-reactive protein (mg/L), America Society of Anaesthesiologists (ASA) grade (1,2,3,4/5), Revised Cardiac Risk Index (RCRI) score (0, 1, 2, 3, 4+), respiratory comorbidities (yes, no), smoking status (non-smoker, current smoker). See Appendix for full protocol definitions.
- Disease: preoperative respiratory support (none, supplemental oxygen, ventilation), timing of SARS-CoV-2 diagnosis (preoperative, postoperative).
- Operation factors: surgical specialty (abdominal, cardiothoracic, head and neck, obstetric, orthopaedic, other), indication for surgery (benign, cancer, trauma), urgency of surgery (elective, emergency), grade of surgery (minor, major), type of anaesthesia (general, regional).

*Data partition*

Our objective was to use all available data to maximise the generalisability and power of our results. Therefore, following TRIPOD guidelines (1), model reliability was assessed by using a temporally distinct validation cohort, with a validation set defined by those patients undergoing surgery from June 1st to July 31st . All patients undergoing surgery in the period of February 1st and May 31st 2020 were part of the derivation cohort, where all model development (i.e. feature selection and model building) were performed. This was estimated to allow optimal sample size requirements in validation sets, with at least 100 predicted events (deaths).

*Missing data*

We checked for possible patterns in missingness across features using the *UpSetR* package. From the 16 candidate predictors, a predictor was excluded if ≥20% of values were missing. Analyses were performed using both complete cases and an imputed dataset. Multiple imputation with chained equations, under the missing at random assumption was done through the *mice* package, generating three different datasets. We pre-planned to use a complete case analysis if there were no important differences in model validation to the imputed dataset.

*Feature selection*

Feature selection from the candidate predictors aimed to optimize the predictor group by finding the smallest independent set of variables with the greatest association to the outcome, to ensure both best performance and minimise overfitting. The methods used were Elastic Net (2), a regularized linear model through the package *glmnet* and Random Forest from the package *ranger*, with variable importance evaluated through the *vip* package and Gini index respectively. More specifically Elastic Net (2) generates sparse linear models whereas Random Forest (RF), an ensemble of decision trees, gives insight on non-linear interactions. These methods were applied on the derivation set, with hyperparameter tuning using 10- and 5-fold resamplings across a regular grid search using *tidymodels* and *recipes* capabilities. By applying these different variable selection methods, we have acknowledged different feature interaction criteria, which has helped to ensure that no important variables were missed out and increased confidence in our results.

The final set of features was determined by merging the feature selection importance ranking of both methods. The criteria used consisted of accounting for both the feature’s ranking and frequency of appearance. These features were then combined with each other, generating different models with which to find the most informative set of predictors. A maximum of 5 features were selected as threshold, given this number offered the best compromise of computing time required and possible information loss; the combination of these 5 features would yield 26 different multivariate sets of predictors to evaluate in three different modelling approaches (78 final models per bootstrap) for the subsequent stage.

*Model building and validation*

To perform model building, the derivation set was split into a 25% testing and 75% training partition. This process was bootstrapped (or randomly repeated) 100 times and for each of the bootstraps, all 26 feature combinations were fitted using three different algorithms (logistic regression, decision trees and random forest). The first two algorithms were chosen because of their relative simplicity for interpretation and well-known use in medical settings as well as their contrasting buildup given they focus on different types of interactions (linear vs non-linear) (3). The third model, random forest, was chosen given it can be considered a complex extension of decision trees and so acts as a useful reference for oversimplification in model performance assessment. Hyperparameters in each of these models were also tuned through grid search and 10-fold cross validation.

For the final decision on the best model combination, performance was evaluated through the mean area under the receiver operating curve (AUROC) value of the 100 bootstraps**.** The standard deviation of our results was also considered in order to assess the stability of our models, as well as sensitivity, specificity and accuracy. We decided a priori to choose the simplest model (less number of independent features) with the highest and most robust performance. Our final model was then built by averaging the coefficients of the 100 models produced for the combination of features selected (4, 5). The final model was then separately applied to both the whole derivation and validation sets, with perfromance assessed through both discrimination using the AUROC and calibration evaluated using Brier score, slope, and intercept (6-9).

Finally, in order to establish the different risk cut-off values, case fatality ratios at different model probability output’s thresholds were assessed in the validation set and checked in the derivation set (10). These thresholds were influenced by distribution of the data and the clinical importance of various cut-offs. The whole dataset, merging both validation and derivation sets was then used to report the final case fatality percentages.

This prediction model is reported in alignment with TRIPOD (1, 11) and PROBAST(12) guidelines. All analysis was performed in R Foundation for Statistical Computing V4.0 (Vienna, Austria) version 4.00 and RStudio through the University of Birmingham’s BlueBEAR High Performance Computing service using rslurm, drake and all packages mentioned above. To aid transparency in research, all scripts and code used have been uploaded to github (<https://github.com/InFlamUOB/CovidSurg>).

*Role of funding source*

The funders of the study had no role in study design, data collection, data analysis, data interpretation, or writing of the report. The corresponding author and analysis group had full access to all the data in the study. The corresponding author and the writing committee had final responsibility for the decision to submit for publication.

**Appendix 2: Results**

*Patient characteristics*

Overall patient demographics and operative factors have been described in Supplementary Table 1 to enable better understanding of the type of patients who received an operation and had peri-operative SARS-coV-2 infection in this cohort. This has also been split between the derivation set and the validation set (Supplementary Table 2), which desmonstates the differences between the two datasets, namely, less comorbid patients were receiving operations. This reflects the systematic change that occurred over time as patient selection and treatment options became available. However, despite this, the risk score continued to perform well between the derivation and validation sets demonstrating flexibility in utility throughout fluctuations in SARS-CoV-2 risk.

*Missing data for feature selection*

Missing data are described in Supplementary Table 1 and patterns of missingness shown in Supplementary Figure 4. Of the 16 preoperative variables selected to enter into feature selection, C-Reactive Protein had to be excluded due to exceeding our *a priori* missing data threshold (≥20%). Although its inclusion may have improved the score’s performance, its inclusion would have reduced the score's global applicability because C-Reactive Protein is not universally measured preoperatively in elective patients. Similarly, whilst including using a wider range of variables, such as imaging based markers or complex blood tests, may have resulted in higher discriminatory performance, this would have undermined our aim to produce a score that could be used globally across high, middle and low income countries.

Three imputed datasets were generated and were found to be consistent and performed well. No more imputed datasets were added as the degree of missingness was small (<5%) and bootstrapping and further cross validation strategies were acknowledged further down the pipeline of work. Results from combined analysis of the imputed and non-imputed datasets are shown in Supplementary Figure 5. Given the similarity of the results, the primary analysis is based on the non-imputed (complete case) dataset.

*Model Building and Validation*

Multivariable models were built using all possible combinations of the five selected features, resulting in 26 model runs (Supplementary Table 8). Correlation structure of preprocessed features are shown in Supplementary Figure 6 and hyperparameter optimisation of the 100 bootstraps are shown in Supplementary Figure 7.

A visual summary of all the operational algorithms performed can be seen in Supplementary Figure 8, with the evaluation of all feature combinations (runs 1-26) of the three different modeling methods at both training and testing partitions. To guide model choice, Figure 2a and Supplementary Figure 9 provide a visual overview of the performance associated with particular features. Figure 2a shows model performance for logistic regression models, which were found to be those with consistent best performance (Supplementary Table 4 and Supplementary Table 5). The guide for feature performance in decision trees and random forest is shown in Supplementary Figure 9.

The smallest, higher performing, and most robust model was a logistic regression model with four features (age, ASA grade, RCRI score, and preoperative respiratory support).

The addition of specialty (logistic regression run 26, mean AUC 0.72, Supplementary Table 4) provided no important improvement in performance to the same model without specialty (logistic regression run 21). Procedure specialty, grade, and urgency in the derivation and validation cohorts is shown in Supplementary Table 9. Complete modelling results are shown in Supplementary Table 10. Logistic regression coefficients of all the 100 models for the selected model (run/model 21) were then averaged (4, 5) and are shown in Supplementary Table 6.

The AUC for the final score was 0.73 (95% confidence interval 0.71-0.74) in the derivation set and 0.80 (0.77-0.83) in the validation set (Figure 4). Calibration in the validation set is shown in Figure 5 (Brier score 0.084, intercept 0.078, slope 1.08).

Mortality rates were studied for different probability risk cut-offs (Supplementary Figure 3), with a balance between distribution and clinical importance leading to cut-off values at ~ 2%, ~10%, ~30%, and ~45% mortality. These showed high concordance between derivation and validation sets (Table 7) with the final mortality percentages in the combined dataset being very similar.

*Post operative mortality*

There was a decrease in overall postoperative mortality from 18.9% in the derivation set (February to May 2020) to 10.7% in the validation set (June to July 2020). This was associated with a change in the characteristics of patients undergoing surgery between the two periods, with fewer older and higher ASA grades patients operated in during the validation set period. Whilst this indicates that surgeons have started to select lower-risk patients for surgery, the validation set mortality rate of 10.7% is still very high, indicating that a more structured and reproducible approach to risk-assessment would improve patient selection.

**Appendix 3: Study definitions**

**American Society of Anaesthesiologists physical status classification (ASA grade)**

ASA grade is a validated system used to assess and communicate a patient’s pre-anaesthesia medical comorbidities (13). The classification system can be used to stratify risks of postoperative complications. The following grades were defined in the study protocol:

- Grade 1: Healthy person
- Grade 2: Mild systemic disease
- Grade 3: Severe systemic disease
- Grade 4: Severe systemic disease that is a constant threat to life
- Grade 5: A moribund person who is not expected to survive without the operation.

**Revised Cardiac Risk Index (RCRI)**

The RCRI represents a multifactorial approach to assessing perioperative cardiac risk. It is calculated as a composite of:

- Procedural risk (intraperitoneal/intrathoracic versus other): in this study all patients undergoing cardiac surgery, gastrointestinal surgery, general surgery, gynaecology, hepatobiliary surgery, thoracic surgery, urology, or vascular surgery procedures scored this point.
- History of ischemic heart disease.
- History of myocardial infarction.
- History of congestive heart failure.
- History of cerebrovascular disease (stroke or transient ischaemic attack).
- History of diabetes mellitus requiring insulin use: in this study all diabetic patients scored this point.
- History of chronic kidney disease (creatinine > 176 μmol/L): in this study all patients reported to have moderate or severe chronic kidney disease scored this point.

Each item adds 1 point to the score. The total score gives an attributable risk score from 0 (lowest risk) to 6 (highest risk) (14).

**Grade of surgery**

Grade of surgery was categorised on the basis of the Bupa schedule of procedures as either minor (minor or intermediate according to the Bupa schedule) or major (major or complex major according to the Bupa schedule) (15).

**Urgency of surgery**

Emergency surgery was defined as procedures classified by the National Confidential Enquiry into Patient Outcome and Death as immediate, urgent, or expedited. Elective surgery was defined as procedures classified by the National Confidential Enquiry into Patient Outcome and Death as scheduled (16).

**References:**

1. Moons KG, Altman DG, Reitsma JB, Ioannidis JP, Macaskill P, Steyerberg EW, et al. Transparent Reporting of a multivariable prediction model for Individual Prognosis or Diagnosis (TRIPOD): explanation and elaboration. Annals of internal medicine. 2015;162(1):W1-W73.

2. Zou H, Hastie T. Regularization and variable selection via the elastic net. Journal of the royal statistical society: series B (statistical methodology). 2005;67(2):301-20.

3. Rosenfeld A, Graham DG, Jevons S, Ariza J, Hagan D, Wilson A, et al. Development and validation of a risk prediction model to diagnose Barrett's oesophagus (MARK-BE): a case-control machine learning approach. The Lancet Digital Health. 2020;2(1):e37-e48.

4. Teschendorff AE. Avoiding common pitfalls in machine learning omic data science. Nature Materials. 2019;18(5):422-7.

5. Chen X, Gole J, Gore A, He Q, Lu M, Min J, et al. Non-invasive early detection of cancer four years before conventional diagnosis using a blood test. Nature communications. 2020;11(1):1-10.

6. Huang Y, Li W, Macheret F, Gabriel RA, Ohno-Machado L. A tutorial on calibration measurements and calibration models for clinical prediction models. Journal of the American Medical Informatics Association. 2020;27(4):621-33.

7. van Walraven C. The Hospital-patient One-year Mortality Risk score accurately predicted long-term death risk in hospitalized patients. Journal of clinical epidemiology. 2014;67(9):1025-34.

8. Steyerberg EW, Vergouwe Y. Towards better clinical prediction models: seven steps for development and an ABCD for validation. European heart journal. 2014;35(29):1925-31.

9. Gupta RK, Harrison EM, Ho A, Docherty AB, Knight SR, van Smeden M, et al. Development and validation of the ISARIC 4C Deterioration model for adults hospitalised with COVID-19: a prospective cohort study. Lancet Respir Med. 2021;11(S2213-2600(20)30559-2).

10. Knight SR, Ho A, Pius R, Buchan I, Carson G, Drake TM, et al. Risk stratification of patients admitted to hospital with covid-19 using the ISARIC WHO Clinical Characterisation Protocol: development and validation of the 4C Mortality Score. BMJ. 2020;371(m4334).

11. Jalali MS, DiGennaro C, Sridhar D. Transparency assessment of COVID-19 models. The Lancet Global Health. 2020;8(12):e1459-e60.

12. Moons KG, Wolff RF, Riley RD, Whiting PF, Westwood M, Collins GS, et al. PROBAST: a tool to assess risk of bias and applicability of prediction model studies: explanation and elaboration. Annals of internal medicine. 2019;170(1):W1-W33.

13. Anesthesiologists. ASo. ASA Physical Status Classification System 2019 [10.08.2020] [Available from: <https://www.asahq.org/standards-and-guidelines/asa-physical-status-classification-system>.

14. Lee TH, Marcantonio ER, Mangione CM, Thomas EJ, Polanczyk CA, Cook EF, et al. Derivation and prospective validation of a simple index for prediction of cardiac risk of major noncardiac surgery. Circulation. 1999;100(10):1043-9.

15. BUPA. Schedule of Procedures 2020 [Available from: <https://codes.bupa.co.uk/procedures>.

16. IC Martin MC, AJG , CMK Hargraves, M Lansdown, M Schubert. National Confidential Enquiry into Perioperative Deaths

Who Operates When? II. A report by the National Confidential Enquiry into Perioperative Deaths 2001/02: NCEPOD; 2004 [Available from: <https://www.ncepod.org.uk/2003report/Downloads/03full.pdf>.

**Appendix 4: Supplementary tables and figures**

**Supplementary Table 1: Patient, disease and operation factors of patients included in the cohort study**

| **Factor** | **Levels** | **Alive**  **N=7030** | **Dead**  **N=1462** | **Missing** | **P-value** |
| --- | --- | --- | --- | --- | --- |
| **Age** | 18-39 years | 1842 (26.2%) | 74 (5.06%) | 0 | <0.001 |
|  | 40-49 years | 778 (11.1%) | 77 (5.27%) |  |  |
|  | 50-59 years | 967 (13.8%) | 155 (10.6%) |  |  |
|  | 60-69 years | 1091 (15.5%) | 257 (17.6%) |  |  |
|  | 70-79 years | 1135 (16.1%) | 378 (25.9%) |  |  |
|  | 80-89 years | 927 (13.2%) | 362 (24.8%) |  |  |
|  | ≥90 years | 290 (4.13%) | 159 (10.9%) |  |  |
| **Sex** | Female | 3558 (50.6%) | 603 (41.3%) | 3 | <0.001 |
|  | Male | 3471 (49.4%) | 857 (58.7%) |  |  |
| **C-reactive protein** | mg/L | 31.0 [6.00;98.0] | 52.0 [13.0;140] | 3011 | <0.001 |
| **Grade of surgery** | Major | 5100 (73.0%) | 1155 (79.6%) | 57 | <0.001 |
|  | Minor | 1884 (27.0%) | 296 (20.4%) |  |  |
| **Anaesthesia** | General | 5159 (73.4%) | 1142 (78.1%) | 0 | <0.001 |
|  | Regional only | 1871 (26.6%) | 320 (21.9%) |  |  |
| **Specialty** | Abdominal surgery | 2920 (41.8%) | 526 (36.3%) | 57 | <0.001 |
|  | Cardiothoracic surgery | 265 (3.79%) | 78 (5.38%) |  |  |
|  | Head and neck surgery | 662 (9.48%) | 173 (11.9%) |  |  |
|  | Obstetric | 772 (11.1%) | 18 (1.24%) |  |  |
|  | Orthopaedic surgery | 2222 (31.8%) | 645 (44.5%) |  |  |
|  | Other surgery | 143 (2.05%) | 11 (0.76%) |  |  |
| **Haemoglobin** | g/dl (IQR) | 120 [102;136] | 111 [95.0;129] | 140 | <0.001 |
| **White cell count** | 10^9/L (IQR) | 9.60 [6.92;13.3] | 10.0 [7.20;14.7] | 173 | 0.001 |
| **Preoperative respiratory support** | None | 4783 (68.9%) | 737 (50.8%) | 100 | <0.001 |
|  | Supplemental oxygen | 1500 (21.6%) | 448 (30.9%) |  |  |
|  | Ventilation | 657 (9.47%) | 267 (18.4%) |  |  |
| **Indication for surgery** | Benign | 4114 (58.5%) | 754 (51.6%) | 3 | <0.001 |
|  | Cancer | 1096 (15.6%) | 231 (15.8%) |  |  |
|  | Trauma | 1818 (25.9%) | 476 (32.6%) |  |  |
| **Urgency of surgery** | Elective | 1436 (20.4%) | 193 (13.2%) | 1 | <0.001 |
|  | Emergency | 5593 (79.6%) | 1269 (86.8%) |  |  |
| **Timing of SARS-CoV-2 diagnosis** | Postoperative | 3015 (42.9%) | 836 (57.2%) | 1 | <0.001 |
|  | Preoperative | 4014 (57.1%) | 626 (42.8%) |  |  |
| **Smoking status** | Non-smoker | 6326 (90.0%) | 1340 (91.8%) | 4 | 0.042 |
|  | Smoker | 702 (9.99%) | 120 (8.22%) |  |  |
| **Respiratory comorbidity** | No respiratory disease | 6136 (87.3%) | 1189 (81.4%) | 4 | <0.001 |
|  | Respiratory disease | 892 (12.7%) | 271 (18.6%) |  |  |
| **Revised Cardiac Risk** **Index** | 0 | 2334 (33.2%) | 332 (22.7%) | 4 | <0.001 |
|  | 1 | 3161 (45.0%) | 557 (38.2%) |  |  |
|  | 2 | 1085 (15.4%) | 370 (25.3%) |  |  |
|  | 3 | 334 (4.75%) | 136 (9.32%) |  |  |
|  | ≥4 | 114 (1.62%) | 65 (4.45%) |  |  |
| **ASA grade** | Grade 1 | 1033 (14.8%) | 33 (2.27%) | 43 | <0.001 |
|  | Grade 2 | 2415 (34.5%) | 248 (17.1%) |  |  |
|  | Grade 3 | 2591 (37.0%) | 695 (47.9%) |  |  |
|  | Grades 4-5 | 958 (13.7%) | 476 (32.8%) |  |  |

Univariate analysis and p-values obtained through the function *compareGroups* package, all continuous variables being non-normal as assessed by the Shapiro-Wilk test and described through the 25^th^ and 75^th^ percentiles. Categorical variables are shown with percentages and P values were calculated depending on the type of variable with Kruskall-Wallis for continuous and chi-squared permutation or fisher test for categorical, depending on the number of classes. ASA: American Society of Anaesthesiologists. Percentages given as proportion of column total. Differences between grouped data tested with Chi-squared test.

**Supplementary Table 2: Patient, disease and operation factors of patients included in the derivation and validation sets.**

|  |  | **Derivation cohort** | | | **Validation cohort** | | |
| --- | --- | --- | --- | --- | --- | --- | --- |
| **Factor** | **Level** | **Alive**  **N=5498** | **Dead**  **N=1279** | **P-value** | **Alive**  **N=1532** | **Dead**  **N=183** | **P-value** |
| **Age** | 18-39 years | 1124 (20.4%) | 45 (3.52%) | <0.001 | 718 (46.9%) | 29 (15.8%) | <0.001 |
|  | 40-49 years | 563 (10.2%) | 62 (4.85%) |  | 215 (14.0%) | 15 (8.20%) |  |
|  | 50-59 years | 756 (13.8%) | 120 (9.38%) |  | 211 (13.8%) | 35 (19.1%) |  |
|  | 60-69 years | 921 (16.8%) | 221 (17.3%) |  | 170 (11.1%) | 36 (19.7%) |  |
|  | 70-79 years | 1025 (18.6%) | 342 (26.7%) |  | 110 (7.18%) | 36 (19.7%) |  |
|  | 80-89 years | 832 (15.1%) | 337 (26.3%) |  | 95 (6.20%) | 25 (13.7%) |  |
|  | ≥90 years | 277 (5.04%) | 152 (11.9%) |  | 13 (0.85%) | 7 (3.83%) |  |
| **Sex** | Female | 2794 (50.8%) | 528 (41.3%) | <0.001 | 764 (49.9%) | 75 (41.0%) | 0.028 |
|  | Male | 2703 (49.2%) | 749 (58.7%) |  | 768 (50.1%) | 108 (59.0%) |  |
| **C-reactive protein** | mg/L | 33.0 [7.00;102] | 49.0 [12.0;137] | <0.001 | 20.0 [5.00;71.2] | 94.5 [24.0;162] | <0.001 |
| **Grade of surgery** | Major | 4023 (73.7%) | 1020 (80.3%) | <0.001 | 1077 (70.6%) | 135 (75.0%) | 0.250 |
|  | Minor | 1435 (26.3%) | 251 (19.7%) |  | 449 (29.4%) | 45 (25.0%) |  |
| **Anaesthesia** | General | 4123 (75.0%) | 995 (77.8%) | 0.039 | 1036 (67.6%) | 147 (80.3%) | 0.001 |
|  | Regional only | 1375 (25.0%) | 284 (22.2%) |  | 496 (32.4%) | 36 (19.7%) |  |
| **Specialty** | Abdominal surgery | 2236 (41.0%) | 430 (33.8%) | <0.001 | 684 (44.8%) | 96 (53.3%) | <0.001 |
|  | Cardiothoracic surgery | 217 (3.98%) | 72 (5.66%) |  | 48 (3.15%) | 6 (3.33%) |  |
|  | Head and neck surgery | 579 (10.6%) | 152 (12.0%) |  | 83 (5.44%) | 21 (11.7%) |  |
|  | Obstetric | 448 (8.21%) | 15 (1.18%) |  | 324 (21.2%) | 3 (1.67%) |  |
|  | Orthopaedic surgery | 1870 (34.3%) | 591 (46.5%) |  | 352 (23.1%) | 54 (30.0%) |  |
|  | Other surgery | 108 (1.98%) | 11 (0.87%) |  | 35 (2.29%) | 0 (0.00%) |  |
| **Haemoglobin (g/dl)** | g/dl | 119 [101;135] | 111 [96.0;130] | <0.001 | 123 [108;139] | 106 [90.0;125] | <0.001 |
| **White cell count (10^9/L)** | 10^9/L | 9.41 [6.90;13.1] | 9.70 [7.00;13.9] | 0.087 | 10.0 [7.00;14.0] | 13.2 [8.67;18.3] | <0.001 |
| **Preoperative respiratory support** | None | 3595 (66.4%) | 662 (52.2%) | <0.001 | 1188 (78.0%) | 75 (41.0%) | <0.001 |
|  | Supplemental oxygen | 1244 (23.0%) | 383 (30.2%) |  | 256 (16.8%) | 65 (35.5%) |  |
|  | Ventilation | 578 (10.7%) | 224 (17.7%) |  | 79 (5.19%) | 43 (23.5%) |  |
| **Indication for surgery** | Benign | 3065 (55.8%) | 623 (48.7%) | <0.001 | 1049 (68.5%) | 131 (71.6%) | 0.624 |
|  | Cancer | 917 (16.7%) | 210 (16.4%) |  | 179 (11.7%) | 21 (11.5%) |  |
|  | Trauma | 1514 (27.5%) | 445 (34.8%) |  | 304 (19.8%) | 31 (16.9%) |  |
| **Urgency of surgery** | Elective | 1132 (20.6%) | 176 (13.8%) | <0.001 | 304 (19.8%) | 17 (9.29%) | 0.001 |
|  | Emergency | 4365 (79.4%) | 1103 (86.2%) |  | 1228 (80.2%) | 166 (90.7%) |  |
| **Timing of SARS-CoV-2 diagnosis** | Postoperative | 2645 (48.1%) | 769 (60.1%) | <0.001 | 370 (24.2%) | 67 (36.6%) | <0.001 |
|  | Preoperative | 2852 (51.9%) | 510 (39.9%) |  | 1162 (75.8%) | 116 (63.4%) |  |
| **Current smoker** | Non-smoker | 4922 (89.5%) | 1180 (92.4%) | 0.002 | 1404 (91.7%) | 160 (87.4%) | 0.073 |
|  | Smoker | 575 (10.5%) | 97 (7.60%) |  | 127 (8.30%) | 23 (12.6%) |  |
| **Respiratory comorbidity** | No respiratory disease | 4710 (85.7%) | 1033 (80.9%) | <0.001 | 1426 (93.1%) | 156 (85.2%) | <0.001 |
|  | Respiratory disease | 787 (14.3%) | 244 (19.1%) |  | 105 (6.86%) | 27 (14.8%) |  |
| **Revised Cardiac Risk** **Index** | 0 | 1734 (31.5%) | 305 (23.9%) | <0.001 | 600 (39.2%) | 27 (14.8%) | <0.001 |
|  | 1 | 2481 (45.1%) | 471 (36.9%) |  | 680 (44.4%) | 86 (47.0%) |  |
|  | 2 | 889 (16.2%) | 320 (25.1%) |  | 196 (12.8%) | 50 (27.3%) |  |
|  | 3 | 294 (5.35%) | 121 (9.48%) |  | 40 (2.61%) | 15 (8.20%) |  |
|  | ≥4 | 99 (1.80%) | 60 (4.70%) |  | 15 (0.98%) | 5 (2.73%) |  |
| **ASA grade** | Grade 1 | 624 (11.4%) | 26 (2.05%) | <0.001 | 409 (26.7%) | 7 (3.83%) | <0.001 |
|  | Grade 2 | 1801 (33.0%) | 210 (16.5%) |  | 614 (40.1%) | 38 (20.8%) |  |
|  | Grade 3 | 2219 (40.6%) | 616 (48.5%) |  | 372 (24.3%) | 79 (43.2%) |  |
|  | Grades 4-5 | 821 (15.0%) | 417 (32.9%) |  | 137 (8.94%) | 59 (32.2%) |  |

Univariate analysis and p-values obtained through the function *compareGroups* package, all continuous variables being non-normal as assessed by the Shapiro-Wilk test and described through the 25^th^ and 75^th^ percentiles. Categorical variables are shown with percentages and P values were calculated depending on the type of variable with Kruskall-Wallis for continuous and chi-squared permutation or fisher test for categorical, depending on the number of classes. ASA: American Society of Anaesthesiologists. Percentages given as proportion of column total. Differences between grouped data tested with Chi-squared test.

**Supplementary Table 3: Feature ranking**

| **Variable Name** | **Top Rank in model** | **Frequency of appearance** |
| --- | --- | --- |
| Age | 1-EN | 8 |
| ASA grade | 3-RF | 3 |
| Revised Cardiac Risk Index | 4-EN | 3 |
| Preoperative respiratory support | 3-EN | 2 |
| Specialty | 7-RF | 2 |
| Haemoglobin (g/dL) | 2-RF | 1 |
| White Cell Count (10^9^/L) | 4-RF | 1 |

Top 7 important features as ranked by Elastic Net (EN) and Random Forest algorithms seen in Figure 2, whereby position in ranking of importance is considered alongside the frequency of appearance of the predictors. From here the top 5 features (Age, ASA grade, Revised Cardiac Risk Index, Preoperative respiratory support and Specialty) were selected and continued to model building.

ASA: American Society of Anaesthesiologists

**Supplementary Table 4: Summary of model selection, ranked by mean area under the curve value**

| **Model** | **Run** | **Mean  AUROC** | **Standard  Deviation** | **Variable names** | **Number of  Variables** |
| --- | --- | --- | --- | --- | --- |
| Logistic Regression | 21 | 0.722 | 0.011 | Age, ASA grade, RCRI score, Preoperative respiratory support | 4 |
| Logistic Regression | 26 | 0.72 | 0.011 | Age, ASA grade, RCRI score, Preoperative respiratory support, Specialty | 5 |
| Logistic Regression | 23 | 0.716 | 0.012 | Age, ASA grade, Preoperative respiratory support, Specialty | 4 |
| Logistic Regression | 12 | 0.715 | 0.012 | Age, ASA grade, Preoperative respiratory support | 3 |
| Logistic Regression | 11 | 0.714 | 0.013 | Age, ASA grade, RCRI score | 3 |
| Logistic Regression | 22 | 0.712 | 0.012 | Age, ASA grade, RCRI score, Specialty | 4 |
| Logistic Regression | 14 | 0.711 | 0.01 | Age, RCRI score, Preoperative respiratory support | 3 |
| Logistic Regression | 1 | 0.711 | 0.013 | Age, ASA grade | 2 |
| Logistic Regression | 24 | 0.71 | 0.01 | Age, RCRI score, Preoperative respiratory support, Specialty | 4 |
| Logistic Regression | 13 | 0.709 | 0.013 | Age, ASA grade, Specialty | 3 |
| Decision Trees | 21 | 0.699 | 0.014 | Age, ASA grade, RCRI score, Preoperative respiratory support | 4 |
| Logistic Regression | 16 | 0.698 | 0.011 | Age, Preoperative respiratory support, Specialty | 3 |
| Decision Trees | 22 | 0.696 | 0.013 | Age, ASA grade, RCRI score, Specialty | 4 |
| Logistic Regression | 3 | 0.696 | 0.012 | Age, Preoperative respiratory support | 2 |
| Decision Trees | 24 | 0.695 | 0.013 | Age, RCRI score, Preoperative respiratory support, Specialty | 4 |
| Decision Trees | 23 | 0.692 | 0.014 | Age, ASA grade, Preoperative respiratory support, Specialty | 4 |
| Decision Trees | 26 | 0.692 | 0.014 | Age, ASA grade, RCRI score, Preoperative respiratory support, Specialty | 5 |
| Logistic Regression | 15 | 0.69 | 0.012 | Age, RCRI score, Specialty | 3 |
| Logistic Regression | 2 | 0.689 | 0.013 | Age, RCRI score | 2 |
| Decision Trees | 13 | 0.687 | 0.026 | Age, ASA grade, Specialty | 3 |

The 20 top performing runs (combination of features and machine learning method) evaluated in the test partition of the derivation. The best performing model as evaluated by the highest AUROC was then selected as final model (Run 21, made up of logistic regression of age ASA grade, RCRI score and preoperative respiratory support). AUROC: Area under the receiving operator characteristic curve; ASA: American Society of Anaesthesiologists

**Supplementary Table 5: Summary of models with highest area under the curve value across included model types**

| **Model** | **Run** | **Mean**  **AUROC** | **Standard  Deviation** | **Variable names** | **Number of  Variables** |
| --- | --- | --- | --- | --- | --- |
| Decision Trees | 21 | 0.699 | 0.014 | Age, ASA grade, RCRI score, Preoperative respiratory support | 4 |
| Decision Trees | 22 | 0.696 | 0.013 | Age, ASA grade, RCRI score, Specialty | 4 |
| Decision Trees | 24 | 0.695 | 0.013 | Age, RCRI score, Preoperative respiratory support, Specialty | 4 |
| Decision Trees | 23 | 0.692 | 0.014 | Age, ASA grade, Preoperative respiratory support, Specialty | 4 |
| Decision Trees | 26 | 0.692 | 0.014 | Age, ASA grade, RCRI score, Preoperative respiratory support, Specialty | 5 |
| Logistic Regression | 21 | 0.722 | 0.011 | Age, ASA grade, RCRI score, Preoperative respiratory support | 4 |
| Logistic Regression | 26 | 0.720 | 0.011 | Age, ASA grade, RCRI score, Preoperative respiratory support, Specialty | 5 |
| Logistic Regression | 23 | 0.716 | 0.012 | Age, ASA grade, Preoperative respiratory support, Specialty | 4 |
| Logistic Regression | 12 | 0.715 | 0.012 | Age, ASA grade, Preoperative respiratory support | 3 |
| Logistic Regression | 11 | 0.714 | 0.013 | Age, ASA grade, RCRI | 3 |
| Random Forest | 26 | 0.654 | 0.014 | Age, ASA grade, RCRI score, Preoperative respiratory support, Specialty | 5 |
| Random Forest | 21 | 0.642 | 0.014 | Age, ASA grade, RCRI score, Preoperative respiratory support | 4 |
| Random Forest | 24 | 0.630 | 0.013 | Age, RCRI score, Preoperative respiratory support, Specialty | 4 |
| Random Forest | 22 | 0.630 | 0.014 | Age, ASA grade, RCRI score, Specialty | 4 |
| Random Forest | 23 | 0.613 | 0.013 | Age, ASA grade, Preoperative respiratory support, Specialty | 4 |

Best performing combination of features for each of the three machine learning methods used (decision trees, logistic regression and random forest). AUROC: Area under the receiving operator characteristic curve; ASA: American Society of Anaesthesiologists; RCRI: Revised Cardiac Risk Index

**Supplementary Table 6: Logistic regression coefficients of final model**

| **Variables** | **Mean** | **Standard Deviation** | **Odds** |
| --- | --- | --- | --- |
| (Intercept) | -3.905692335 | 0.158016766 | 0.020127015 |
| Age: 40-49 years | 0.676864016 | 0.13768028 | 1.967697378 |
| Age: 50-59 years | 0.875244522 | 0.110975064 | 2.399461944 |
| Age: 60-69 years | 1.313345464 | 0.105959876 | 3.718593345 |
| Age: 70-79 years | 1.622864021 | 0.106067253 | 5.067583217 |
| Age: 80-89 years | 1.868802925 | 0.105702546 | 6.480534067 |
| Age: ≥90 years | 2.215434947 | 0.117880167 | 9.165394704 |
| ASA grade: Grade 2 | 0.319931615 | 0.131520891 | 1.377033593 |
| ASA grade: Grade 3 | 0.705422094 | 0.132624263 | 2.024701118 |
| ASA grade: Grades 4-5 | 1.235581852 | 0.139084125 | 3.440379731 |
| RCRI score: 1 | 0.154318345 | 0.058124435 | 1.166862293 |
| RCRI score: 2 | 0.456189957 | 0.062779551 | 1.578050077 |
| RCRI score: 3 | 0.508920987 | 0.078963076 | 1.663495293 |
| RCRI score: ≥4 | 0.73768561 | 0.099174282 | 2.09109031 |
| Preoperative respiratory support: oxygen | 0.27267317 | 0.043161647 | 1.313470892 |
| Preoperative respiratory support: ventilated | 0.727455318 | 0.054730085 | 2.0698069 |

Final logistic model coefficients, obtained by averaging the coefficients generated in each bootstrap (100) for the top performing model (run 21, i.e., logistic regression of features age, ASA grade, RCRI score and preoperative respiratory support ) ASA: American Society of Anaesthesiologists; RCRI: Revised Cardiac Risk Index

**Supplementary Table 7: Performance metrics of model probability output at different cut-off values in both derivation and validation cohorts.**

| **Derivation** | | | | | | | | | | |
| --- | --- | --- | --- | --- | --- | --- | --- | --- | --- | --- |
| **Cut-off Value** | **Patients  (%)** | **TP** | **TN** | **FP** | **FN** | **Sensitivity  (%)** | **Specificity  (%)** | **PPV  (%)** | **NPV  (%)** | **Mortality  (%)** |
| ≤0.05 | 1031 (15.93) | 1202 | 1008 | 4241 | 23 | 98.1 | 19.2 | 22.1 | 97.8 | 2.2 |
| ≤0.2 | 3683 (56.89) | 861 | 3319 | 1930 | 364 | 70.3 | 63.2 | 30.8 | 90.1 | 9.9 |
| ≥0.2 | 2791 (43.11) | 861 | 3319 | 1930 | 364 | 70.3 | 63.2 | 30.8 | 90.1 | 30.8 |
| ≥0.5 | 85 (1.31) | 40 | 5204 | 45 | 1185 | 3.3 | 99.1 | 47.1 | 81.4 | 47.1 |
| **Validation** | | | | | | | | | | |
| ≤0.05 | 723 (43.35) | 166 | 711 | 779 | 12 | 93.3 | 47.7 | 17.6 | 98.3 | 1.7 |
| ≤0.2 | 1366 (81.89) | 88 | 1276 | 214 | 90 | 49.4 | 85.6 | 29.1 | 93.4 | 6.6 |
| ≥0.2 | 302 (18.11) | 88 | 1276 | 214 | 90 | 49.4 | 85.6 | 29.1 | 93.4 | 29.1 |
| ≥0.5 | 8 (0.48) | 4 | 1486 | 4 | 174 | 2.2 | 99.7 | 50.0 | 89.5 | 50.0 |

TP: True positive; TN: True negative; FP: False positive; FN: False negative; PPV: Positive predictive value; NPV: Negative predictive value. Mortality = estimated absolute mortality risk for all patients in group.

**Supplementary Table 8: Summary of feature combinations in model runs**

| **Run** | **Variable names** | **Number of  Variables** |
| --- | --- | --- |
| 1 | Age, ASA grade | 2 |
| 2 | Age, Revised Cardiac Risk Index | 2 |
| 3 | Age, Preoperative respiratory support | 2 |
| 4 | Age, Speciality | 2 |
| 5 | ASA grade, Revised Cardiac Risk Index | 2 |
| 6 | ASA grade, Preoperative respiratory support | 2 |
| 7 | ASA grade, Speciality | 2 |
| 8 | Revised Cardiac Risk Index, Preoperative respiratory support | 2 |
| 9 | Revised Cardiac Risk Index, Speciality | 2 |
| 10 | Preoperative respiratory support, Speciality | 2 |
| 11 | Age, ASA grade, Revised Cardiac Risk Index | 3 |
| 12 | Age, ASA grade, Preoperative respiratory support | 3 |
| 13 | Age, ASA grade, Speciality | 3 |
| 14 | Age, Revised Cardiac Risk Index, Preoperative respiratory support | 3 |
| 15 | Age, Revised Cardiac Risk Index, Speciality | 3 |
| 16 | Age, Preoperative respiratory support, Speciality | 3 |
| 17 | ASA grade, Revised Cardiac Risk Index, Preoperative respiratory support | 3 |
| 18 | ASA grade, Revised Cardiac Risk Index, Speciality | 3 |
| 19 | ASA grade, Preoperative respiratory support, Speciality | 3 |
| 20 | Revised Cardiac Risk Index, Preoperative respiratory support, Speciality | 3 |
| 21 | Age, ASA grade, Revised Cardiac Risk Index, Preoperative respiratory support | 4 |
| 22 | Age, ASA grade, Revised Cardiac Risk Index, Speciality | 4 |
| 23 | Age, ASA grade, Preoperative respiratory support, Speciality | 4 |
| 24 | Age, Revised Cardiac Risk Index, Preoperative respiratory support, Speciality | 4 |
| 25 | ASA grade, Revised Cardiac Risk Index, Preoperative respiratory support, Speciality | 4 |
| 26 | Age, ASA grade, Revised Cardiac Risk Index, Preoperative respiratory support, Speciality | 5 |

ASA: American Society of Anaesthesiologists.

**Supplementary Table 9: Procedure specialty, grade, and urgency in the derivation and validation cohorts**

| Specialty | Elective surgery | | Emergency surgery | |
| --- | --- | --- | --- | --- |
|  | Minor surgery | Major surgery | Minor surgery | Major surgery |
| Derivation cohort* | | | | |
| Breast surgery | 17 | 33 | 2 | 4 |
| Cardiac surgery | 0 | 60 | 0 | 92 |
| Gastrointestinal & general surgery | 38 | 369 | 776 | 794 |
| Gynaecology | 11 | 66 | 19 | 52 |
| Head & Neck surgery | 93 | 41 | 333 | 30 |
| Hepatobiliary surgery | 0 | 134 | 0 | 190 |
| Neurosurgery | 3 | 31 | 10 | 190 |
| Obstetrics | 9 | 93 | 28 | 333 |
| Ophthalmology | 6 | 0 | 8 | 0 |
| Orthopaedics | 9 | 86 | 122 | 1,975 |
| Other surgery | 14 | 10 | 7 | 1 |
| Plastic surgery | 3 | 0 | 3 | 10 |
| Thoracic surgery | 4 | 57 | 30 | 46 |
| Urology | 26 | 57 | 96 | 38 |
| Vascular surgery | 1 | 35 | 18 | 215 |
| Validation cohort** | | | | |
| Breast surgery | 2 | 8 | 1 | 0 |
| Cardiac surgery | 0 | 9 | 0 | 17 |
| Gastrointestinal & general surgery | 25 | 67 | 313 | 204 |
| Gynaecology | 4 | 15 | 10 | 12 |
| Head & Neck surgery | 18 | 17 | 16 | 12 |
| Hepatobiliary surgery | 0 | 28 | 0 | 62 |
| Neurosurgery | 0 | 5 | 1 | 35 |
| Obstetrics | 5 | 49 | 23 | 250 |
| Ophthalmology | 1 | 0 | 7 | 0 |
| Orthopaedics | 4 | 28 | 25 | 301 |
| Other surgery | 2 | 0 | 5 | 1 |
| Plastic surgery | 0 | 2 | 0 | 6 |
| Thoracic surgery | 0 | 6 | 7 | 15 |
| Urology | 10 | 5 | 13 | 12 |
| Vascular surgery | 0 | 9 | 2 | 37 |

*Procedure data missing for 2 elective and 46 emergency patients

**Procedure data missing for 2 elective and 7 emergency patients

**Supplementary Table 10: Summary of all models and runs, ordered by area under the curve value**

| **Model** | **Run** | **Mean  AUC** | **Standard  Deviation** | **Variable names** | **Number of  Variables** |
| --- | --- | --- | --- | --- | --- |
| Logistic Regression | 21 | 0.722 | 0.011 | Age, ASA grade, RCRI score, Preoperative respiratory support | 4 |
| Logistic Regression | 26 | 0.72 | 0.011 | Age, ASA grade, RCRI score, Preoperative respiratory support, Speciality | 5 |
| Logistic Regression | 23 | 0.716 | 0.012 | Age, ASA grade, Preoperative respiratory support, Speciality | 4 |
| Logistic Regression | 12 | 0.715 | 0.012 | Age, ASA grade, Preoperative respiratory support | 3 |
| Logistic Regression | 11 | 0.714 | 0.013 | Age, ASA grade, RCRI score | 3 |
| Logistic Regression | 22 | 0.712 | 0.012 | Age, ASA grade, RCRI score, Speciality | 4 |
| Logistic Regression | 14 | 0.711 | 0.01 | Age, RCRI score, Preoperative respiratory support | 3 |
| Logistic Regression | 1 | 0.711 | 0.013 | Age, ASA grade | 2 |
| Logistic Regression | 24 | 0.71 | 0.01 | Age, RCRI score, Preoperative respiratory support, Speciality | 4 |
| Logistic Regression | 13 | 0.709 | 0.013 | Age, ASA grade, Speciality | 3 |
| Decision Trees | 21 | 0.699 | 0.014 | Age, ASA grade, RCRI score, Preoperative respiratory support | 4 |
| Logistic Regression | 16 | 0.698 | 0.011 | Age, Preoperative respiratory support, Speciality | 3 |
| Decision Trees | 22 | 0.696 | 0.013 | Age, ASA grade, RCRI score, Speciality | 4 |
| Logistic Regression | 3 | 0.696 | 0.012 | Age, Preoperative respiratory support | 2 |
| Decision Trees | 24 | 0.695 | 0.013 | Age, RCRI score, Preoperative respiratory support, Speciality | 4 |
| Decision Trees | 23 | 0.692 | 0.014 | Age, ASA grade, Preoperative respiratory support, Speciality | 4 |
| Decision Trees | 26 | 0.692 | 0.014 | Age, ASA grade, RCRI score, Preoperative respiratory support, Speciality | 5 |
| Logistic Regression | 15 | 0.69 | 0.012 | Age, RCRI score, Speciality | 3 |
| Logistic Regression | 2 | 0.689 | 0.013 | Age, RCRI score | 2 |
| Decision Trees | 13 | 0.687 | 0.026 | Age, ASA grade, Speciality | 3 |
| Logistic Regression | 25 | 0.687 | 0.013 | ASA grade, RCRI score, Preoperative respiratory support, Speciality | 4 |
| Logistic Regression | 18 | 0.684 | 0.014 | ASA grade, RCRI score, Speciality | 3 |
| Logistic Regression | 19 | 0.679 | 0.012 | ASA grade, Preoperative respiratory support, Speciality | 3 |
| Logistic Regression | 17 | 0.679 | 0.013 | ASA grade, RCRI score, Preoperative respiratory support | 3 |
| Logistic Regression | 5 | 0.675 | 0.013 | ASA grade, RCRI score | 2 |
| Logistic Regression | 7 | 0.675 | 0.013 | ASA grade, Speciality | 2 |
| Logistic Regression | 4 | 0.674 | 0.013 | Age, Speciality | 2 |
| Decision Trees | 11 | 0.67 | 0.017 | Age, ASA grade, RCRI score | 3 |
| Decision Trees | 15 | 0.669 | 0.016 | Age, RCRI score, Speciality | 3 |
| Logistic Regression | 6 | 0.669 | 0.013 | ASA grade, Preoperative respiratory support | 2 |
| Decision Trees | 25 | 0.666 | 0.015 | ASA grade, RCRI score, Preoperative respiratory support, Speciality | 4 |
| Decision Trees | 14 | 0.666 | 0.017 | Age, RCRI score, Preoperative respiratory support | 3 |
| Decision Trees | 18 | 0.665 | 0.014 | ASA grade, RCRI score, Speciality | 3 |
| Logistic Regression | 20 | 0.66 | 0.012 | RCRI score, Preoperative respiratory support, Speciality | 3 |
| Random Forest | 26 | 0.654 | 0.014 | Age, ASA grade, RCRI score, Preoperative respiratory support, Speciality | 5 |
| Decision Trees | 17 | 0.645 | 0.045 | ASA grade, RCRI score, Preoperative respiratory support | 3 |
| Random Forest | 21 | 0.642 | 0.014 | Age, ASA grade, RCRI score, Preoperative respiratory support | 4 |
| Logistic Regression | 9 | 0.641 | 0.015 | RCRI score, Speciality | 2 |
| Decision Trees | 16 | 0.641 | 0.047 | Age, Preoperative respiratory support, Speciality | 3 |
| Random Forest | 24 | 0.63 | 0.013 | Age, RCRI score, Preoperative respiratory support, Speciality | 4 |
| Random Forest | 22 | 0.63 | 0.014 | Age, ASA grade, RCRI score, Speciality | 4 |
| Logistic Regression | 10 | 0.627 | 0.012 | Preoperative respiratory support, Speciality | 2 |
| Logistic Regression | 8 | 0.625 | 0.015 | RCRI score, Preoperative respiratory support | 2 |
| Decision Trees | 19 | 0.617 | 0.065 | ASA grade, Preoperative respiratory support, Speciality | 3 |
| Decision Trees | 20 | 0.615 | 0.03 | RCRI score, Preoperative respiratory support, Speciality | 3 |
| Decision Trees | 4 | 0.613 | 0.068 | Age, Speciality | 2 |
| Random Forest | 23 | 0.613 | 0.013 | Age, ASA grade, Preoperative respiratory support, Speciality | 4 |
| Decision Trees | 2 | 0.605 | 0.073 | Age, RCRI score | 2 |
| Decision Trees | 12 | 0.604 | 0.077 | Age, ASA grade, Preoperative respiratory support | 3 |
| Random Forest | 11 | 0.598 | 0.013 | Age, ASA grade, RCRI score | 3 |
| Random Forest | 14 | 0.591 | 0.013 | Age, RCRI score, Preoperative respiratory support | 3 |
| Random Forest | 25 | 0.591 | 0.013 | ASA grade, RCRI score, Preoperative respiratory support, Speciality | 4 |
| Random Forest | 12 | 0.582 | 0.011 | Age, ASA grade, Preoperative respiratory support | 3 |
| Random Forest | 15 | 0.577 | 0.012 | Age, RCRI score, Speciality | 3 |
| Random Forest | 13 | 0.576 | 0.015 | Age, ASA grade, Speciality | 3 |
| Decision Trees | 9 | 0.573 | 0.026 | RCRI score, Speciality | 2 |
| Random Forest | 18 | 0.569 | 0.011 | ASA grade, RCRI score, Speciality | 3 |
| Random Forest | 17 | 0.562 | 0.01 | ASA grade, RCRI score, Preoperative respiratory support | 3 |
| Random Forest | 16 | 0.555 | 0.011 | Age, Preoperative respiratory support, Speciality | 3 |
| Random Forest | 19 | 0.546 | 0.014 | ASA grade, Preoperative respiratory support, Speciality | 3 |
| Decision Trees | 5 | 0.544 | 0.068 | ASA grade, RCRI score | 2 |
| Random Forest | 1 | 0.544 | 0.016 | Age, ASA grade | 2 |
| Random Forest | 5 | 0.541 | 0.014 | ASA grade, RCRI score | 2 |
| Random Forest | 2 | 0.54 | 0.01 | Age, RCRI score | 2 |
| Random Forest | 20 | 0.536 | 0.009 | RCRI score, Preoperative respiratory support, Speciality | 3 |
| Random Forest | 8 | 0.518 | 0.008 | RCRI score, Preoperative respiratory support | 2 |
| Decision Trees | 7 | 0.517 | 0.044 | ASA grade, Speciality | 2 |
| Random Forest | 3 | 0.513 | 0.009 | Age, Preoperative respiratory support | 2 |
| Random Forest | 9 | 0.512 | 0.006 | RCRI score, Speciality | 2 |
| Random Forest | 4 | 0.51 | 0.006 | Age, Speciality | 2 |
| Decision Trees | 10 | 0.506 | 0.024 | Preoperative respiratory support, Speciality | 2 |
| Decision Trees | 8 | 0.506 | 0.019 | RCRI score, Preoperative respiratory support | 2 |
| Random Forest | 7 | 0.501 | 0.004 | ASA grade, Speciality | 2 |
| Random Forest | 6 | 0.501 | 0.005 | ASA grade, Preoperative respiratory support | 2 |
| Random Forest | 10 | 0.501 | 0.003 | Preoperative respiratory support, Speciality | 2 |
| Decision Trees | 1 | 0.5 | 0 | Age, ASA grade | 2 |
| Decision Trees | 3 | 0.5 | 0 | Age, Preoperative respiratory support | 2 |
| Decision Trees | 6 | 0.5 | 0 | ASA grade, Preoperative respiratory support | 2 |

AUC: Area under the receiving operator characteristic curve; ASA: American Society of Anaesthesiologists; RCRI: Revised Cardiac Risk Index.

**Supplementary Figure 1: Feature Selection according to impurity importance ranking of Random Forest ranger model and importance ranking of EN models.**

**Importance was calculated through the *vip* and r*anger* gini index parameters for each model respectively and ranked. For all 7 top ranking features of each model, frequency of appearance was assessed and results shown in Table 3. For EN,** the reported coefficients are the highest non-sparse coefficients generated by the linear model and the relationship with the outcome was included and coded here as low risk for negatively associated with mortality and high risk for highly associated with mortality. ASA Grade (America Society of Anaesthesiologists Grade).

**Supplementary Figure 2: Model calibration in the validation set**

A. Calibration plot for the predicted probabilities in the validation set. In red a loess smoothed line linking calibration at bins of 10 with confidence intervals calculated. 45 degree black angle line shows the perfect calibration plot (slope 1 , intercept 0). Below we have a histogram of probability estimates.

B. Boxplot and beeswarm plot of predicted probabilities in validation set, stratified by outcome.

C. Assessment of calibration in both validation and derivation sets (Brier score, slope and intercept evaluated through val.prob function from *rms* package)

**Supplementary Figure 3: Graphic representation of mortality/case fatality, according to probability risk groups**

Tile plot of Table 7 cut-off values for the complete cases dataset (n=8492), whereby case fatality rates are associated to a range of probability outcomes, grouping patients into a color coded description of risk. Probability outcome range from 0 to 0.8 and are split into 4 groups corresponding to case fatality ratios of approximately 2%, 10 %, 30% and 50% as seen in all three derivation, validation and complete datasets .

ASA: America Society of Anaesthesiologists; RCRI: Revised Cardiac Risk Index.

**Supplementary Figure 4: Missing value exploration during exploratory data analysis (package naniar)**

**Supplementary Figure 5: Combined analysis of the 3 imputed and non-imputed datasets.**

I

mputation was performed using *mice* and the default methods of predictive mean matching, logistic regression and polytomous regression imputation. The main analysis results: final model coefficients, and performance as evaluated by AUROC and calibration paramters are here depicted.

**Supplementary Figure 6:** **Correlation structure of preprocessed features**


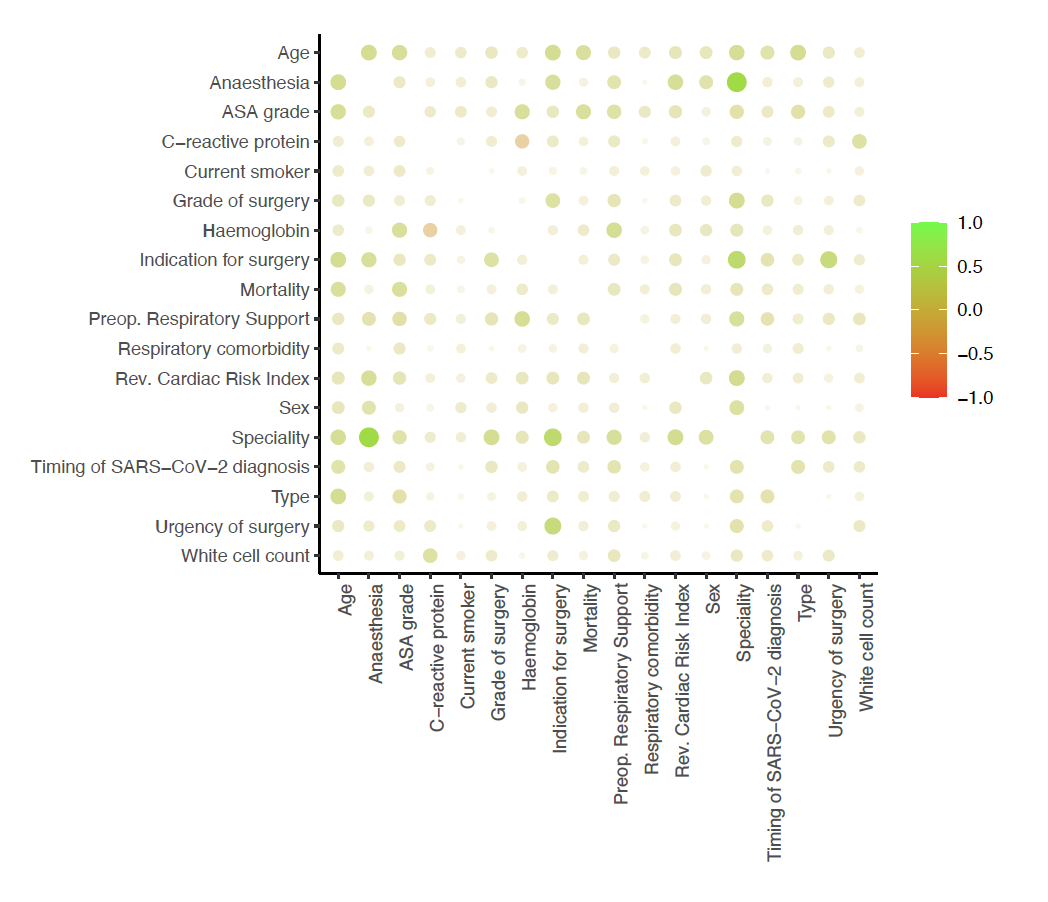


Pairwise association between all variables in a data-frame. In particular nominal vs nominal with Chi-square, numeric vs numeric with Pearson correlation, and nominal vs numeric with ANOVA [ANalysis Of VAriance] (Adopted from https://stackoverflow.com/a/52557631/590437).

**Supplementary Figure 7:** **Hyperparameter optimization**

F

or each bootstrap, 10 fold cross validation and hyperparamter tuning was performed, with cost complexity, min_n and tree depth optimized in decision tree algorithm and min_n and trees in random forest.

In each bootstrap the following hyperparamters were optimized through a 10 fold cross validation (Cost complexity, min n and tree depth for decision trees and min n and trees for random forest. Glm has no hyperparamters.

**Supplementary Figure 8: Prediction model building and performance metrics**

All selected features were merged, yielding 26 different feature combinations (or runs) as seen in Supplementary Table 2. These runs were then modeled through three different algorithms (RandomForest, DecisionTrees and Logistic Regression). Performance was assessed through the metrics Accuracy, AUROC, Sensitivity and Specificity and both training (triangles) and testing (circles) is reported.

**Supplementary Figure 9:** **Model selection.**

A. Decision Tree

B. Random Forest

Performance of all different feature combinations/runs divided by each of the features. Colour coded according to performance values (Mean AUC [Area Under the Curve] of 100 Bootstraps).
